# The influence of white matter lesions on the electric field in transcranial electric stimulation

**DOI:** 10.1016/j.nicl.2022.103071

**Published:** 2022-06-02

**Authors:** Benjamin Kalloch, Konstantin Weise, Leonie Lampe, Pierre-Louis Bazin, Arno Villringer, Mario Hlawitschka, Bernhard Sehm

**Affiliations:** a Max Planck Institute for Human Cognitive and Brain Sciences, Department of Neurology, Leipzig, Germany; b Leipzig University of Applied Science, Faculty of Computer Science and Media, Leipzig, Germany; c Max Planck Institute for Human Cognitive and Brain Sciences, Methods and Development Group “Brain Networks”, Leipzig, Germany; d Technische Universität Ilmenau, Instiute of Biomedical Engineering and Informatics, Ilmenau, Germany; e Technische Universität Ilmenau, Advanced Electromagnetics Group, Ilmenau, Germany; f University of Amsterdam, Faculty of Social and Behavioural Sciences, Amsterdam, The Netherlands; g Department of Neurology, Martin Luther University of Halle-Wittenberg, Germany

**Keywords:** $∥E∥$, The magnitude of the electric field, mean($∥E∥$), The mean electric field magnitude given the uncertain electric conductivity, var($∥E∥$), The variance of the electric field magnitude given the uncertain electric conductivity, HPC, Hippocampus, TH, Thalamus, GM, gray matter, WM, white matter, WML, white matter lesion, CSF, cerebrospinal fluid, Transcranial electrical stimulation, Transcranial direct current stimulation, White matter lesions, White matter hyperintensities, Uncertainty analysis, Aging effects

## Abstract

**Background:**

Transcranial direct current stimulation (tDCS) is a promising tool to enhance therapeutic efforts, for instance, after a stroke. The achieved stimulation effects exhibit high inter-subject variability, primarily driven by perturbations of the induced electric field (EF). Differences are further elevated in the aging brain due to anatomical changes such as atrophy or lesions. Informing tDCS protocols by computer-based, individualized EF simulations is a suggested measure to mitigate this variability.

**Objective:**

While brain anatomy in general and specifically atrophy as well as stroke lesions are deemed influential on the EF in simulation studies, the influence of the uncertainty in the change of the electrical properties of the white matter due to white matter lesions (WMLs) has not been quantified yet.

**Methods:**

A group simulation study with 88 subjects assigned into four groups of increasing lesion load was conducted. Due to the lack of information about the electrical conductivity of WMLs, an uncertainty analysis was employed to quantify the variability in the simulation when choosing an arbitrary conductivity value for the lesioned tissue.

**Results:**

The contribution of WMLs to the EF variance was on average only one tenth to one thousandth of the contribution of the other modeled tissues. While the contribution of the WMLs significantly increased (p≪.01) in subjects exhibiting a high lesion load compared to low lesion load subjects, typically by a factor of 10 and above, the total variance of the EF didnot change with the lesion load.

**Conclusion:**

Our results suggest that WMLs do not perturb the EF globally and can thus be omitted when modeling subjects with low to medium lesion load. However, for high lesion load subjects, the omission of WMLs may yield less robust local EF estimations in the vicinity of the lesioned tissue. Our results contribute to the efforts of accurate modeling of tDCS for treatment planning.

## Highlights

• Sensitivity analysis allows the simulation of tDCS with uncertain conductivities.
• White matter lesions (WML) have no global influence on the electric field in tDCS.
• In subjects with a high lesion load, a local influence can be observed.
• In low to medium lesion load subjects, explicit modeling of WML is not required.

## 1 Introduction

Transcranial direct current stimulation (tDCS) is currently researched as a therapeutic tool, for example, for relieving pain (David et al., 2018), promoting rehabilitation (Awosika and Cohen, 2019), or attenuating cognitive decline (Summers et al., 2016). Older adults represent an important target group for tDCS applications as most neurological diseases like Alzheimer’s disease, Parkinson’s disease, or stroke predominantly manifest in the aging brain. However, tDCS studies report a high inter-subject variability in the stimulation effects (van Asseldonk and Boonstra, 2016, Ammann et al., 2017). A relationship between the electric field in tES simulations and physiological modulations assessed by functional MRI or motor evoked potential measurements was recently revealed (Jamil et al., 2020, Laakso et al., 2019). This, in turn, attributes the electric field a role in the individual modulation of cortical function induced by tDCS. Moreover, modeling studies identified an immediate impact of subject-specific anatomical differences on the distribution of the electric field within the subject’s head (Laakso et al., 2015, Kim et al., 2013, Im et al., 2018, Filmer et al., 2019). These anatomical differences become more pronounced in the aging brain when considering large-scale structural brain changes such as atrophy (Indahlastari et al., 2020, Woods et al., 2019, Mahdavi et al., 2018) and brain lesions (Wagner et al., 2007, Datta et al., 2011, Minjoli et al., 2017). An individualized electrode montage (Laakso et al., 2017, Parazzini et al., 2017) and current dosage (Evans et al., 2020) informed by numerical computer simulations of tDCS, taking into account such anatomical variations, are suggested measures to mitigate the response variability of tDCS, particularly for elderly subjects as a recent review on tDCS for the aging brain concluded (Habich et al., 2020).

Electrostatic simulations as the underlying technology of individualized tDCS therapy crucially depend on an accurate representation of the electrically relevant structures of the head of individual subjects (Shahid et al., 2011, Opitz et al., 2015, Sadleir et al., 2010). During the aging process, the gray matter structure (Salat et al., 2004) but also the white matter is subject to major changes (Liu et al., 2017). Microstructural alterations like the disruption of white matter tracts, vessel impairments such as cerebral microangiopathy in the presence of vascular risk factors, inflammation, or the loss of myelination may cause atrophy and lesions of the white matter tissue. Despite their frequent manifestation in the aging brain (De Leeuw et al., 2001), white matter lesions (WMLs), or leukoaraiosis, have only very recently gained attention in the context of tDCS simulations (Indahlastari et al., 2021).

To investigate the influence of WMLs on the electric field by means of a simulation study, they must be representable geometrically and physically in a head model. A segmentation of WMLs from MR images can be performed automatically (Shiee et al., 2010, Lampe et al., 2019), allowing their geometric representation. However, the change in the electrical properties of the lesioned white matter tissue is not quantified in the literature. Even the conductivity of healthy tissue varies among subjects (Katoch et al., 2018), rendering the conductivity of all tissues in the human head, but especially that of lesioned white matter, an uncertain input to the simulation. Fixed default conductivities, like the usage of cerebrospinal fluid for the physical modeling of lesions (Wagner et al., 2007, Datta et al., 2011, Minjoli et al., 2017, Indahlastari et al., 2021), may yield inaccuracies in the simulation results, which an uncertainty analysis can quantify.

An uncertainty analysis is a promising tool to model tDCS simulations with uncertain inputs. The physical properties of the head model are represented by a multi-dimensional input space instead of a set of fixed conductivity values. A relation between this input space and the output quantities (i.e. the electric field) is established and statistics such as the mean electric field magnitude, its variance, and the contribution of each tissue to that variance can be derived.

This process is a computationally expensive task. The input space must be sampled sufficiently enough, performing a complete, standard tDCS simulation on each sampling iteration to determine the relation to the output quantity reliably. One technique to mitigate this computational effort is the so-called generalized polynomial chaos expansion, which efficiently determines a surrogate model of the output quantity based on fewer samples, requiring much less computational resources (Saturnino et al., 2019).

This technique was previously introduced in tDCS and TMS case studies in a single young, healthy adult (Saturnino et al., 2019, Schmidt et al., 2015). McCann et al. recently employed this approach to assess the influence of age-related conductivity changes of the skull bone on the electric field distribution during a tDCS application (McCann and Beltrachini, 2021). Investigating a wider range of phenotypes in a group uncertainty analysis and considering abnormal brain tissue remain open tasks.

Here, the influence of white matter lesions on the electric field distribution during tDCS with two different electrode setups is investigated by means of a computational uncertainty analysis using the generalized polynomial chaos expansion. We expected an elevated total variance of the electric field induced by an increasing contribution of the WMLs to the variance proportional to the lesion load. To systematically assess the influence of the lesion load on the electric field and to account for the spatial variability of the lesions, simulations were performed using the imaging data of in total 88 subjects. They were assigned to one of four groups according to their Fazekas score (Fazekas et al., 1987) with a parametrically increasing lesion load ranging from an absence of lesions (Fazekas 0) to a high lesion load (Fazekas 3). All tissue classes were modeled uncertain, with WMLs exhibiting the highest uncertainty. The contribution of the WMLs to the total variance of the computed electric field is assessed, representing the robustness of the simulation results given incorrectly modeled electrical conductivity of the lesioned tissue. Our results inform whether white matter lesion tissue must be considered as a separate structure for accurate modeling of tDCS in the elderly population, contributing to the efforts of individualized tDCS therapy guided by computer simulations in elderly subjects and patients.

## 2 Methods

### 2.1 Imaging data

T1-weighted magnetization prepared rapid gradient echo (MPRAGE) and T2-weighted fluid-attenuated inversion recovery (FLAIR) head images of each subject were selected from a pool of 2029 datasets. These imaging data were collected previously on a MAGNETOM Verio scanner (Siemens, Erlangen, Germany) using a 32-channel head receiver coil and a body transmitter coil as part of the large cross-sectional imaging study of the Leipzig Research Centre for Civilization Diseases (LIFE study) (Loeffler et al., 2015). Detailed MR acquisition parameters can be found in Supplementary Section S1.

### 2.2 Subject sample

**Fig. 1:**
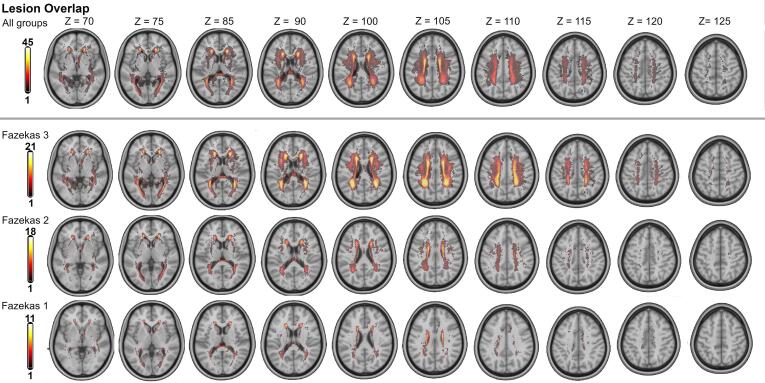
Lesion overlays of the analyzed groups. *Overlays of the spatially normalized white matter lesions of all subjects (top row) and within the individual groups at various slices of the volume. The color of a voxel represents the number of subjects that exhibit lesioned white matter tissue at that location.*

Imaging data of 88 subjects, gender (45 female) and age-matched (70.83 yrs., sd. 4.15 yrs.), were selected from the database of the LIFE study. Subjects were assigned to four groups according to their Fazekas score (Fazekas et al., 1987), which quantified the amount of lesioned tissue in the periventricular and the deep white matter. To exclude differences in brain athropy between groups as a possible source of variation, the normalized total brain volume for each subject was assessed in SIENAX (Smith et al., 2002) from FSL v6.0 (Smith et al., 2004) (see Supplementary Section S3). See Table 1 for a detailed overview of the groups. Fig. 1 displays a summarized overlay of the white matter lesions in all groups and across all subjects. Refer to Supplementary Fig. S1 for exemplary MR images of single subjects from each group.

**Table 1:** Characteristics of the subject sample. *In total, 88 subjects evenly distributed across four groups with increasing lesion load (assessed on the Fazekas scale) with matching age were randomly selected. The age range was determined by the age of the Fazekas 3 subjects, which were exclusively aged 59 or older.*

**Group** *Lesion load*	**Fazekas 0** *Absence*	**Fazekas 1** *Low*	**Fazekas 2** *Medium*	**Fazekas 3** *High*
**# subjects**	22 *(11 ♂)*	22 *(11♂)*	22 *(11♂)*	22 *(12♂)*
**Age (yrs)**	70.67 (sd: 1.51)	71.02 (sd.: 1.86)	70.99 (sd.: 1.62)	70.63 (sd.: 6.85)
**Percentage of affected white matter**	0.0 (sd: 0.0)	0.72 (sd.: 0.7)	2.11 (sd.: 1.81)	8.94 (sd.: 3.48)

### 2.3 TDCS simulations

The tDCS simulations were conducted using our workflow (Kalloch et al., 2020), relying on the finite volume method (FVM) toolkit OpenFOAM (v.7) (The OpenFOAM Foundation, 2020) for the electromagnetic simulations. Electrodes were modeled and positioned in the 3D-modeling software Blender (v2.79) (Blender Foundation, 2020). The head mesh was generated using our custom tool, which combines surface-based and image-based meshing using a Delaunay refinement-based implementation from the Computational Geometry Algorithms Library (CGAL v.4.13.1) (Fabri et al., 2009). The head models of the subjects in this simulation study comprised the structures skin, skull and enclosed air cavities, the subarachnoid cerebrospinal fluid (CSF), the CSF in the ventricles, gray matter (GM), white matter (WM), and white matter lesion (WMLs). The image-based meshing was applied to the lesioned tissue, the internal air, and the ventricles. These tissues would disrupt a nested arrangement, a topological requirement of the surface-based meshing, which was applied for the remaining parts. The resulting meshes contained between $3.5\cdot 10^{6}$ and $4\cdot 10^{6}$ tetrahedral elements. The meshes were optimized by reducing the global mesh energy using a CGAL-implementation of Lloyd’s algorithm (Chen, 2004, Du et al., 1999), and Delaunay slivers, i.e. flat tetrahedra, were removed. The meshes were inspected visually. Their quality was validated using the OpenFOAM tool *checkMesh,* which acknowledged the generated meshes to be suitable for the subsequent FVM-simulations in terms of mesh non-orthogonality, element skewness, element aspect ratio, element volume, and face area in all cases. The T1-weighted head images were segmented using our segmentation pipeline (Kalloch et al., 2018), which relies on robust, atlas-based segmentation approaches that are implemented in the Java Image Science Toolkit (JIST v.3.2) (Lucas et al., 2010), an extension to the Medical Image Processing, Analysis, and Visualization (MIPAV v7.8) (Mcauliffe et al., 2001) software. This entailed a three-step, semi-automatic segmentation procedure. First, the skin tissue and skull were segmented by registering 20 individual template segmentation images from the BrainWeb database to the T1 image of each subject. Voxels with the highest probability of being skin or skull were identified in a majority-voting process specified by the STAPLE algorithm (Warfield et al., 2004). Second, the multi‐object geometric deformable model (MGDM) algorithm (Bogovic et al., 2013) was employed to segment the intracranial compartments of the head, including deep, subcortical structures, in a topology-preserving manner. This ensured a continuous boundary of the structures cerebrospinal-fluid, gray matter, and white matter. Third, the air-filled cavities of the skull were extracted using a pseudo-CT template from (Rorden et al., 2012) that was co-registered to each T1 image. These intermediate segmentation images were merged into one image, which was post-processed by a series of image morphological operations to ensure a suitable quality for the head mesh generation. The WMLs did not interfere with these procedures, but they were also not segmented. Instead, they were segmented automatically from the T2-FLAIR head images, where WMLs are expressed as hyperintensities. The involved segmentation process of the white matter hyperintensities was based on an adapted version of the lesion-TOADS algorithm (Shiee et al., 2010) and is detailed in (Lampe et al., 2019).

To compute the electric field, the quasi-static form of Maxwell’s equations was solved by our custom solver application employing the OpenFOAM API. OpenFOAM implements the cell-centered finite-volume method. As such, it operates on cell centers instead of nodes and result fields are cell-based instead of point-based. The outer boundaries of the electrodes were assigned Dirichlet boundary conditions. The electric potential φ at the center of each tetrahedral mesh element was computed solving $\nabla \cdot \left(\sigma \nabla \varphi \right)=0$ in the head volume conductor models with piecewise constant electrical conductivity σ. The involved Laplace operator was discretized using a Gauss discretization scheme with linear interpolation at a residual of $10^{-6}$. The solution was iterated until the residual of the entire system of equations fell below $10^{-5}$. The electric field E as the gradient of the potential field φ, E=-∇φ, at the center of each tetrahedral element was discretized using a least-squares gradient scheme. Its solution was derived by our solver application as well.

The magnitude of the electric field at the cortical mid-layer of each head model was of particular interest for the subsequent analyses. The laminar package of the neuroimaging processing library Nighres (Huntenburg et al., 2018) was employed to compute the cortical laminae in an anatomically informed manner (Waehnert et al., 2014) and the mid-layer was extracted. The Marching Cubes (Lorensen and Cline, 1987) implementation of ParaView 5.6.3 (Ahrens et al., 2005) was used to create the surface representation of the boundary of the mid-layer segmentation image. The surface was smoothed in Meshlab (Cignoni et al., 2008) using the Taubin smoothing algorithm (λ=0.5,μ=-0.53) (Taubin, 1995) and remeshed using the isotropic remeshing capability of CGAL 4.13.1. The mid-layer surfaces were created independently of the respective head volume meshes and were, thus, not embedded in their structure. Instead, after each completed simulation pass, the resulting electric field magnitude was interpolated onto the mid-layer nodes from the cell data of the head volume mesh using an interpolation scheme based on weighted linear interpolation. Supplementary Section S4 elaborates more on that interpolation.

#### 2.3.1 Simulation case setup

**Fig. 2:**
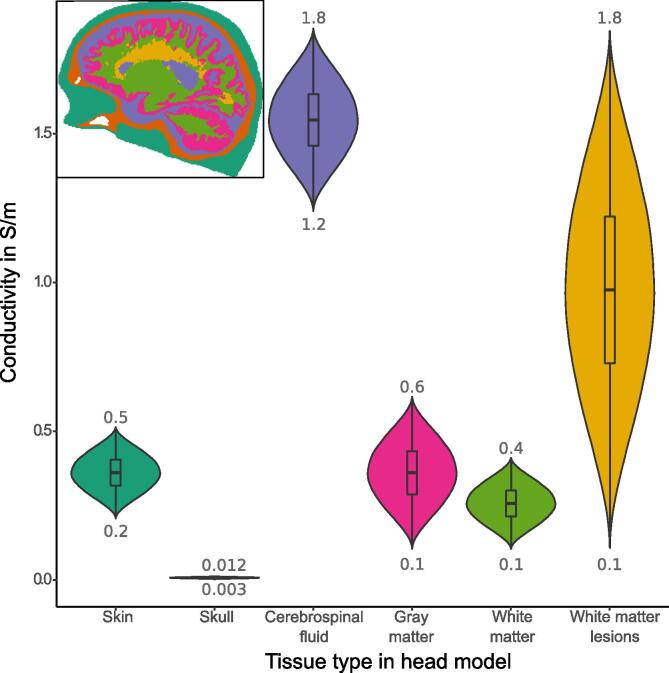
Illustration of the conductivity ranges and their probability density function of the head model compartments. *Different colors represent corresponding tissue types in the exemplary segmentation image in the top left corner. The electrical conductivity values of the structures of the head model were distributed according to a beta-distribution function as shown by violin plots* (shape parameters: α=3,β=3*, for illustrative purposes the y-axis of the distributions was normalized in this plot*)*.*

**Fig. 3:**
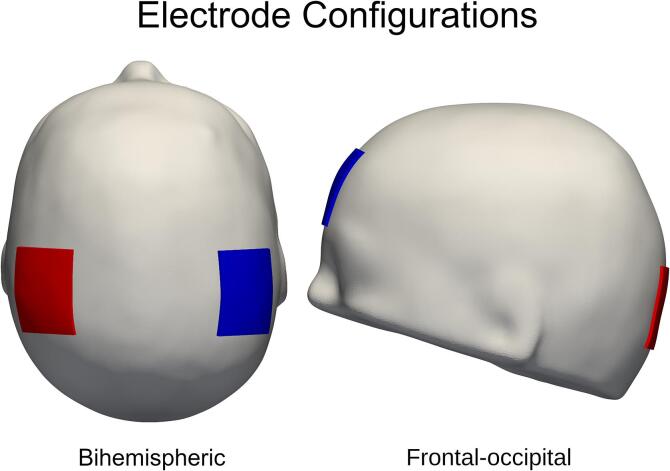
Illustration of the electrode setup. *All simulations were conducted using a bihemispheric electrode setup over the 10*–*20 coordinates C3 & C4 and a frontal-occipital setup over the coordinates FPZ & OZ.*

Each head model exhibited the same seven structures, namely skin, skull, the air cavities of the skull, cerebrospinal fluid (CSF), gray matter (GM), white matter (WM), and the WMLs. As part of the sensitivity analysis, their homogenous, isotropic electrical conductivity values were not fixed but modeled as random variables $\sigma_{i}.$ Each variable was characterized by a beta distribution (shape parameters α=3,β=3) and bound within a specified range of conductivity values (Fig. 2) based on a previous study (Saturnino et al., 2019). A beta distribution was chosen for its boundedness and resemblance to a normal distribution given the mentioned shape parameters. WMLs exhibited the highest conductivity range, representing the increased uncertainty in their true conductivity value due to insufficient evidence from the literature. The range was selected to cover the entire spread of conductivity values in the human brain. The conductivity of air was fixed to 10^-15^ S/m, acting as an insulator. Two electrode setups were simulated, each using quadratic 25 cm^2^ pad electrodes (Fig. 3). In setup 1, the bihemispheric setup, the electrodes were positioned on both hemispheres over the 10–20 electrode coordinates C3 and C4 aimed at motor cortex stimulation. The second setup, the frontal-occipital setup, was chosen to maximize the distance between the electrodes to yield a more extensive subcortical distribution of the electric field (Gomez-Tames et al., 2020). This setup was used before to stimulate arousal (Mauri et al., 2015). The electrodes were positioned over the 10–20 coordinates Oz and Fpz. A current strength of 2 mA was applied in both setups.

### 2.4 Uncertainty analysis

In general, an uncertainty analysis aims to determine the variation of the output quantity q of a system due to the uncertainties of its n input quantities $\xi =[\xi_{1},\cdots ,\xi_{n}]$ spanning a multi-dimensional input space Ξ. The uncertainty of a quantity is described by its probability density function (PDF), representing the likelihood of any arbitrary interval of the quantity. The PDFs of the input quantities are defined by their probability distribution within a finite range. The PDF of the output quantity can be estimated, in a naïve fashion, by computing a sufficiently high number of samples of the output quantity with varying input values. Depending on the modeled problem, computing a single sample of the output quantity may require up to several minutes of computation time rendering the naïve Monte Carlo sampling of the input space not feasible.

The generalized polynomial chaos expansion (gPC) is an established approach to drastically reduce the number of simulations by constructing a surrogate of the computationally expensive original model of the output quantity. The surrogate model is represented by joint polynomial basis functions $\Psi_{a_{k}}\left(\xi \right)$ assembled from orthonormal (in space Ξ) polynomials $\psi_{a_{k}^{\left(i\right)}}^{\left(i\right)}\left(\xi_{i}\right)$, which are defined for each input variable $\xi_{i}$

(1) $$\Psi_{a_{k}}\left(\xi \right)=\prod_{i=1}^{n}\psi_{a_{k}^{\left(i\right)}}^{\left(i\right)}\left(\xi_{i}\right).$$

The degree of the polynomials is denominated by the multi-index $a_{k}\in A,$ k∈{1,⋯,K}, where K denotes the total number of joint basis functions.

The polynomial series with its coefficients $u_{k}$ replaces the actual system under investigation by approximating the true mapping from the input to the output space (Crestaux et al., 2009) at the location r given the input quantities ξ

(2) $$q\left(\xi ,r\right)\approx \overset{\sim }{q}\left(\xi ,r\right)=\underset{a_{k}\in A}{\sum }u_{k}\left(r\right)\Psi_{a_{k}}\left(\xi \right).$$

Consequently, the input space can be sampled more efficiently since it merely involves evaluating a polynomial function instead of evaluating the modeled problem. Finally, to establish the surrogate model, far fewer samples are required than for the setup of the actual output PDF by the Monte Carlo method.

#### 2.4.1 Application of the generalized polynomial chaos expansion for the sensitivity analysis of tDCS simulations

For our tDCS simulations as the system under investigation, the electric field magnitude $|\left|E\right||$ represents the output quantity and the homogenous, isotropic electrical conductivity values of each structure in the head model constitute the input quantities,$\sigma =[\sigma_{1},\cdots ,\sigma_{n}]$. In a previous study (Saturnino et al., 2019), the electric field magnitude was found to be the most sensitive to the uncertainty in tissue conductivity compared to the direction of the electric field and the general electric field pattern.

Calculating one sample of the output quantity for a tDCS simulation-based uncertainty analysis implies performing a full simulation with a randomly (according to the input PDFs) selected fixed conductivity for each tissue compartment. Every simulation pass, thus, represents a mapping from the input space (conductivities) onto the output space ($||E||$). For the sensitivity analysis of the tDCS simulations, the computational effort was mitigated by approximating the electric field magnitude locally by a gPC-series based on the approach described in (Saturnino et al., 2019) and implemented in the Python package pygpc (Weise et al., 2020).

The gPC of the electric field magnitude was determined only at the cortical mid-layer and the regions of interest. The mid-layer as the main site of the cortical neurons is considered the central area of interest for the stimulation. The gPC of the electric field magnitude was not computed for the entire head model because the electric field at the interface between two tissues, for example, at the cortical surface, tends to be less smooth between local samples, resulting in larger approximation errors.

With the gPC of the electric field magnitude, the mean electric field magnitude and its variance, given the uncertain input, can be derived directly from the (gPC) coefficients (Saturnino et al., 2019) for each mid-layer node and each mesh element within the regions of interest. To investigate the contribution of the uncertainty of the electrical conductivity of each tissue to the variance of the electric field magnitude, variance-based importance measures, so-called Sobol indices, were computed directly from the coefficients of the gPC series. The Sobol index indicates the sensitivity of the output quantity, that is, the electric field magnitude, to a particular input variable $\sigma_{i}$, i.e. the electrical conductivity of tissue *i*, or to a combination of multiple input variables. In addition, it is influenced by the uncertainty of the input variables meaning the shape and range of their probability density function. The sum of the Sobol indices of all input variables and their interactions equals the total variance of the electric field magnitude.

#### 2.4.2 Technical realization of the sensitivity analysis

On an Intel Core i7 6700 workstation equipped with an SSD drive and 32 GB RAM, the simulation time for one simulation pass per subject was approximately 8 min. Between 51 and 96 simulations were necessary per subject to expand the gPC series with a residual of $10^{-3}$. This resulted in third-order polynomial series with 34 to 64 coefficients. Up to 4 simulation passes were performed in parallel, which typically resulted in a total computation time of 2–4 h for the uncertainty analysis of one subject.

The average relative error across all test cases between the electric field magnitude computed by the polynomial series and the actual simulated value was 0.56% (sd: 0.2%).

### 2.5 Statistical analysis

The uncertainty analyses focused on determining the mean electric field magnitude, the associated variance, and the Sobol indices as result quantities. We distinguish between two levels of means and variances of the electric field magnitude. The first level is the mean electric field magnitude mean(‖E‖)) and its associated variance var(‖E‖)) due to the uncertainty in the electrical conductivity of the structures in the head model. These result quantities of the uncertainty analysis are defined at each node of the cortical mid-layer and each mesh element within the volumetric regions of interest. The second level is obtained by spatially averaging these result quantities on the cortical mid-layer in subject space (in the following denoted as “whole-brain”) and within eight regions of interest (ROIs, Supplementary Section S5), creating a spatial average of the mean electric field magnitude as well as a spatial average of the variance of the electric field magnitude within the analyzed regions. In the following, we report these spatial averages of the mentioned result quantities at the cortical mid-layer (and within the ROIs in Supplementary Section S5). To gain an additional estimate of the result quantities outside the regions of interest and particularly in the vicinity of the WMLs, we further present individual subject data from a sampling line running from electrode to electrode through the entire intracranial volume in Supplementary Section S6.

#### 2.5.1 Regions of interest

Here, results from the entire cortical mid-layer, denoted as whole-brain results, are presented. Further regions of interest (ROIs) were defined at four locations on the cortical mid-layer and by four deep structures. The cortical mid-layer ROIs were chosen as the cortex represented the primary target site for tDCS. The selected deep brain regions were reported to likewise receive a pronounced electric field magnitude (Gomez-Tames et al., 2020). Furthermore, the electric field in those deep regions was expected to experience a more substantial influence of the white matter and white matter lesions. See Supplementary Section S5 for further details on the regions of interest, their creation, and ROI-specific results.

#### 2.5.2 Statistical methods

Statistics of the spatially averaged results of the uncertainty analysis were computed in R v.3.4.4 (R Core Team, 2020, Wickham, 2016) for the four Fazekas groups: Fazekas 0 (absence of lesions), Fazekas 1 (low lesion load), Fazekas 2 (medium lesion load), and Fazekas 3 (high lesion load). Normality was determined using the Shapiro-Wilk test. Significant deviations between group means for normally distributed samples were tested with a one-way ANOVA with the Fazekas groups as factors and corrected for multiple comparisons, taking into account the number of regions of interest. A Bonferroni corrected paired-samples *t*-test was used as a post-hoc test. Non-normally distributed samples were tested with the Kruskal-Wallis (KW) rank test and corrected for multiple comparisons. Subjects were again grouped according to their Fazekas score. A post-hoc test was conducted using the Bonferroni-corrected Dunn’s test. The effect size measure $\eta^{2}$ was calculated for significant KW tests based on their H-statistic (Tomczak and Tomczak, 2014).

## 3 Results

**Fig. 4:**
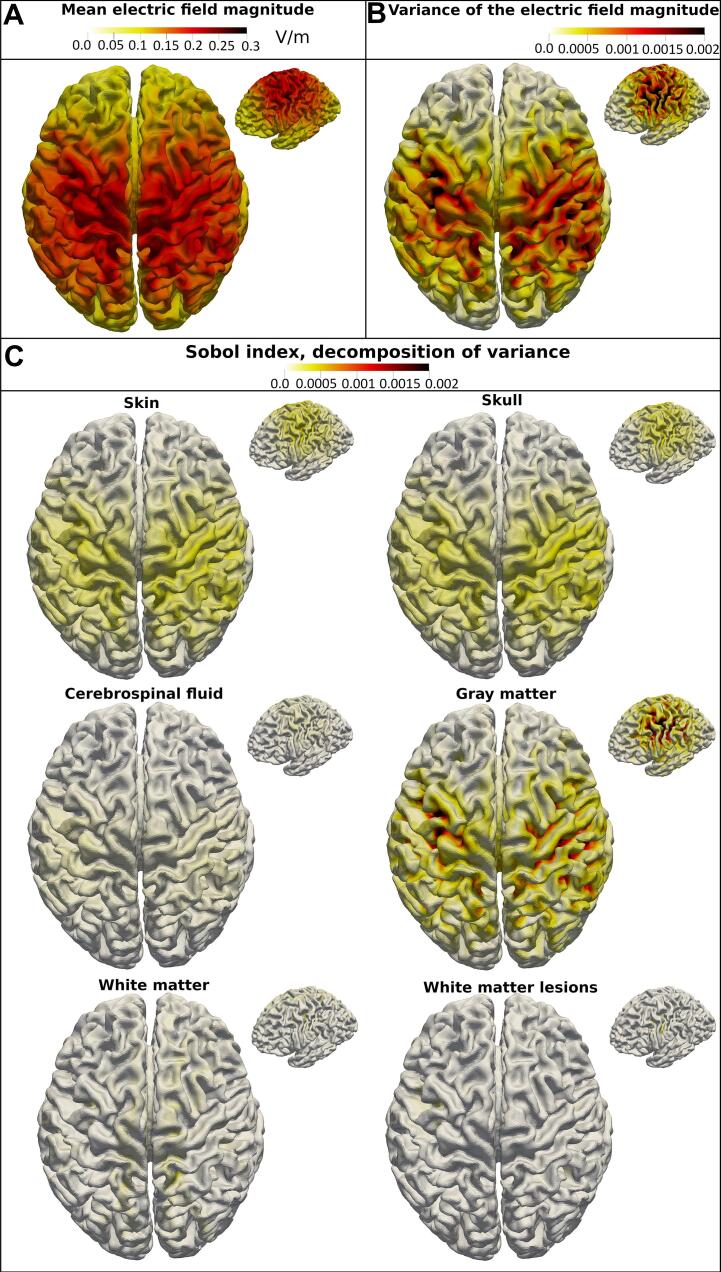
Illustration of the main outcome measures of the sensitivity analysis with the bihemispheric electrode montage. *(A) The mean electric field magnitude, (B) the associated total variance due to the uncertain input, and (C) the Sobol indices of all tissue types are displayed on the cortical mid-layer of a representative single subject from the high lesion load group (Fazekas 3).*

**Fig. 5:**
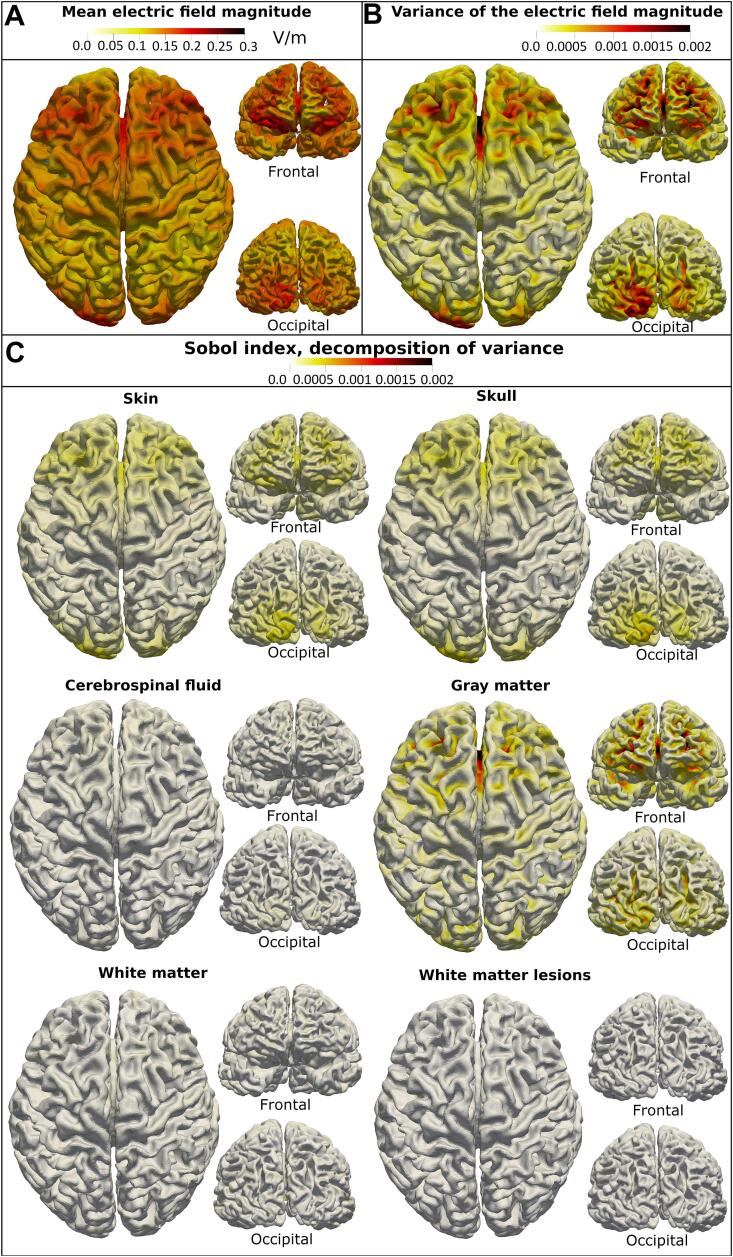
Illustration of the main outcome measures of the sensitivity analysis with the frontal (FPZ)-occipital (OZ) electrode montage. *(A) The mean electric field magnitude, (B) the associated total variance due to the uncertain input, and (C) the Sobol indices of all tissue types are displayed on the cortical mid-layer of a representative single subject from the high lesion load group (Fazekas 3).*

The electric field magnitude is analyzed in terms of its mean, mean(‖E‖), and its variance caused by the uncertainty in tissue conductivity as well as the Sobol indices representing the decomposition of the total variance into the contributions of each input variable. See Fig. 4, Fig. 5 for a visualization of the magnitudes of these metrics on the mid-layer of an exemplary subject from the Fazekas 3 group with both electrode montages. The electric field shows the typical diffuse pattern induced by tDCS. In both electrode setups, the variance of the electric field magnitude is the strongest in the sulci. The magnitude of the Sobol indices of skin and skull is highest in the area under the electrodes. The gray matter Sobol index peaks in the sulci underneath the electrodes. The pattern of the Sobol index of the cerebrospinal fluid is similar to the pattern of skin and skull but less pronounced. The Sobol indices of white matter and WMLs are small compared to the Sobol indices of the other tissue classes. The white matter Sobol index exhibits its highest magnitude medially. The Sobol index of the white matter lesions peaks in distinct areas where the lesions are close to the cortical sheath. In the frontal-occipital electrode montage, the pattern of the Sobol indices of white matter and WMLs are unidentifiable on the mid-layer, indicating that their influence might be further reduced in this electrode setup.

By further analyzing the Sobol index of the white matter lesions, their impact on the electric field can be inferred.

### 3.1 Analyses of the mean electric field magnitude and its variance

**Fig. 6:**
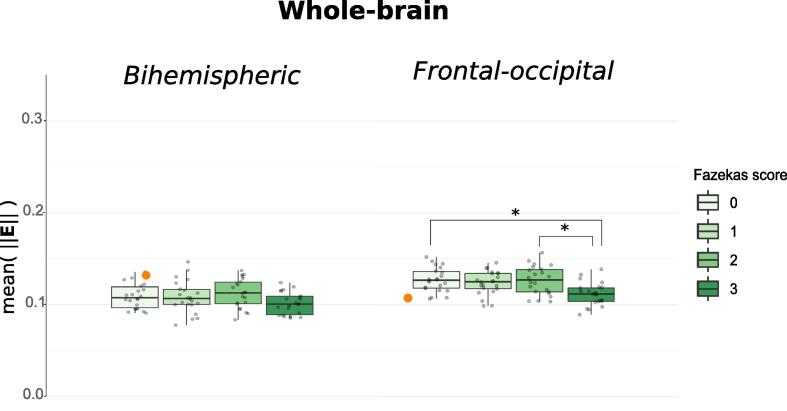
Group-wise boxplots of the mean electric field magnitude with both electrode montages. *Values were averaged on a whole-brain level for every subject (represented as individual dots). Boxplots provide a group comparison. For comparison, the average* mean *electric field magnitude on a whole-brain level from the uncertainty analysis of a young adult* (Saturnino et al., 2019) *was marked with an orange dot within the scatter-plot data of the Fazekas 0 group.*

The spatially averaged mean electric field magnitude, mean(‖E‖), on the whole-brain level is depicted in Fig. 6 and Supplementary Tables S1.1 and S1.2.

Generally, no significant difference in mean(‖E‖) between groups could be found on a whole-brain level and in all ROIs for the bihemispheric electrode setup (for detailed ROI results see Supplementary Section S5.2.1). However, for the frontal-occipital setup, group means of mean(‖E‖) differed significantly (Supplementary Table S3.2) on the whole-brain-level ($p=.001,\eta^{2}=.152$) and in the M1 ROIs (M1 left: $p\ll .001,\eta^{2}=.198$, M1 right $:p\ll .001,\eta^{2}=.193)$. A similar pattern could be observed for the variance of the electric field magnitude (Supplementary Section S5.2.2, Supplementary Table 3.2). Post-hoc analyses revealed that both differences were mediated by a reduced mean(‖E‖) in the Fazekas 3 group compared to all other groups except the Fazekas 1 group (Supplementary Table S4.2).

### 3.2 Analyses of the Sobol indices

**Fig. 7:**
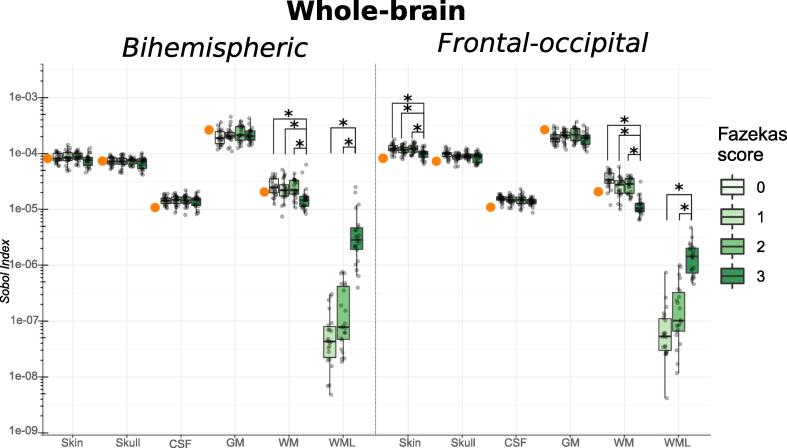
Group-wise boxplots of the Sobol indices of all tissue classes that were modeled uncertain on the whole brain level with both electrode montages. *Note that the results are shown in* log*-scale. Values were averaged on a whole brain level for every subject (represented as individual dots). Boxplots provide a group comparison. For comparison, the average Sobol index on a whole-brain level from the uncertainty analysis of our earlier study of a young adult* (Saturnino et al., 2019) *was marked with an orange dot within the scatter plot data of the Fazekas 0 group.*

The analyzed Sobol indices of order one on the whole-brain level are plotted in Fig. 7 (and Supplementary Tables S2.1 – S2.4) for both electrode setups.

Across all groups and conditions, the group averaged WML Sobol index was only a fraction of the Sobol indices of all other investigated structures (i.e. typically between a tenth to a thousandth, depending on the Fazekas group and tissue type) and, thus, on average the lowest contributor to the electric field variance. However, the contribution was modulated by the lesion load. As expected, the Sobol index of WML from all three groups was consistently the highest in the Fazekas 3 group across all conditions and decreased with decreasing lesion load. Overall, the difference in the Sobol index of WML tissue between the Fazekas 1 and Fazekas 3 group was at least one order of magnitude (i.e. a factor of ten) in both electrode setups. Consequently, the group average WML Sobol indices differed significantly between the high and medium lesion load group (Fazekas 2, Dunn’s test: p≪.001) as well as the high and low lesion group (Fazekas 1, Dunn’s test: p≪.001) (Supplementary Table 4.1 & 4.2). Similarly, there was a significant difference in the group average white matter Sobol index, which was primarily driven by a significantly decreased contribution of healthy white matter to the electric field variance in the Fazekas 3 group (bihemispheric setup; Fazekas 0: ρ = 0.0002, Fazekas 1: ρ = 0.0049, Fazekas 2: ρ = 0.0016. frontal-occipital setup; Fazekas 0: ρ ≪ 0.001, Fazekas 1: ρ = 0.0001, Fazekas 2: ρ ≪ 0.001). The mid-layer and deep ROIs generally confirm these observations with varying effect sizes (Supplementary Section S5.2.3, Supplementary Tables S2.1 – S2.4, 3.1 & 3.2, 4.1 – 4.3).

The strongest influence on a single-subject level on the mid-layer was found in a location within the right Electrode ROI in the bihemispheric setup in a Fazekas 3 subject with a contribution of 11.25% to the total variance. In the deep ROIs, the 99th-percentile highest, single-subject WML Sobol index reflected a contribution of 91.8% to the total electric field variance at a distinct location in the right thalamus in a Fazekas 3 subject when using the bihemispheric electrode montage. Notably, the white matter lesions of this subject were directly adjacent to the right thalamus.

## 4 Discussion

In this work, the impact of white matter lesion (WML) tissue on the electric field magnitude during the application of transcranial direct current stimulation was assessed using a group-level computational uncertainty analysis. Two electrode montages were simulated, a bihemispheric setup targeting the motor cortex and a frontal-occipital setup targeting deeper, subcortical structures. The mean electric field magnitude, its associated total variance, and decomposition of this variance into the contributions of each tissue by means of Sobol indices were analyzed at the cortical midlayer and in superficial cortical and deeper regions of interest in four groups with increasing lesion load. A consistent pattern for both electrode montages was a significant increase in the contribution of white matter lesion tissue to the variance of the electric field magnitude in the high lesion load group compared to the low lesion load group. This increase could be detected in all analyzed regions of interest but varied in strength. However, the contribution of the white matter lesion tissue to the electric field variance was on average the lowest compared to the other tissue classes within all ROIs. Data on a single-subject level from a sampling line through the intracranial compartment suggested that a major part of the electric field variance was accounted for by WMLs only directly within the lesioned tissue and its immediate surroundings with the highest radius in a Fazekas 3 subject. Concludingly, WMLs could be omitted in most head models. Still, additional modeling effort may be required for an accurate simulation of subjects with a high lesion load if the lesion location is close to the stimulation site or when deeper subcortical structures are targeted.

White matter lesions, also known as leukoaraiosis, are characterized by the absence of apparent clinical symptoms. However, they may co-occur with several neurological diseases that develop in the aging brain, such as Parkinson’s disease (PD), Alzheimer’s disease, or stroke. TDCS interventions for improving gait in PD (Lee et al., 2019), for slowing down cognitive decline in Alzheimer’s disease (Cruz Gonzalez et al., 2018), or facilitating the rehabilitation process after a stroke (Biou et al., 2019, Orrù et al., 2020) are currently being researched. A problem common to all these intervention studies is response variability, that is, the observation that the treatment shows an effect in some patients but not all, with no evident pattern (van Asseldonk and Boonstra, 2016, Ammann et al., 2017). One possible explanation for this variability in the stimulation effect is the subject-specific anatomy of the human head that irregularly perturbs the induced electric field (Laakso et al., 2015, Kim et al., 2013, Im et al., 2018, Filmer et al., 2019). Atrophy and brain lesions, consequences of the aging brain, further amplify anatomical differences. Individualizing the tDCS protocol (Parazzini et al., 2017, Evans et al., 2020, Habich et al., 2020) according to anatomical differences (Datta et al., 2011) may increase the stimulation effect and thereby the treatment success. A key to individualization is an accurate computer simulation of the electric field considering the individual anatomy, as studies show a linkage between electric field calculations and physiological responses (Jamil et al., 2020, Laakso et al., 2019, Antonenko et al., 2019). Modeling studies have investigated the influence of atrophy and stroke lesions on the electric field and deemed them a decisive factor in modeling the aging brain (Indahlastari et al., 2020, Mahdavi et al., 2018, Minjoli et al., 2017). Our results augment the knowledge on accurate modeling of tDCS in the aging brain by investigations of sub-cortical alteration of the white matter fiber structure, leukoaraiosis. In contrast to previous studies on stroke lesions (Wagner et al., 2007, Datta et al., 2011, Minjoli et al., 2017) and white matter lesions (Indahlastari et al., 2021), we did not model white matter lesions using the physical properties of existing structures, for example, cerebrospinal fluid. Instead, we introduced the lesions as a new tissue type, with a distinct uncertainty in tissue conductivity. This method mitigated approximation errors in the modeling due to a simplified conductivity profile of the lesioned tissue.

An entirely different approach to investigate the influence of WMLs on the electric field during tDCS was taken recently by Indahlastari et al. (2021). In this study, FEM simulations of subjects exhibiting WMLs were conducted with the presence and artificial absence of their WMLs. A difference measure between both modes of simulation was established and correlated to the individual total lesion volume. The lesioned tissue was physically modeled as CSF and the electrode setup was different from our two setups. Despite these fundamental differences, similar findings were reported: brain regions with the most changes due to the WML tissue were found primarily in the white matter compartment surrounding the lesioned tissue. Our uncertainty analysis provides robust support for the rather local influence of the WMLs. Besides, with the Sobol decomposition of the electric field variance, we contribute an estimate of the robustness of the simulated electric field magnitude in the presence of WMLs on the cortical level and in deeper brain regions.

Despite the significantly higher contribution of WMLs to the total variance of the electric field magnitude in the Fazekas 3 group in both electrode setups, this variance was not increased across all conditions and ROIs. Instead, the contribution of healthy white matter tissue decreased in the Fazekas 3 group in all ROIs of both electrode montages. This indicates 1) that the WMLs did not cause a global perturbation of the electric field and 2) that healthy and lesioned white matter share their contribution to the total variance. The shared volume of healthy and lesioned white matter in the brain may be a possible explanation for their shared contribution to the total variance — a higher lesion load results in a lower volume of healthy white matter.

The local influence of the WMLs on the electric field variance is further supported by three observations: 1) elevated levels of the WML Sobol index could only be found up to 4.3 mm distant from WML areas on the intracranial volume sampling line (Supplementary Section S6), 2) the exemplary visualizations of the Sobol indices on the mid-layer surface show only faint spots of minimally increased WML Sobol indices, 3) despite the closer location of deep regions of interest to the white matter lesions, the WML Sobol index was still the lowest in the deep ROIs except for one subject in the Fazekas 3 group where the WMLs were directly adjacent to the right thalamus yielding a peak contribution of the WMLs to the electric field variance of 91.8 % at distinct locations in the thalamus.

From both electrode setups, general conclusions concerning the influence of white matter lesions on the electric field could be drawn. However, a few differences between both setups were apparent. Whereas in the mid-layer ROIs, no significant difference in the spatially averaged mean electric field magnitude between the groups could be identified in the bihemispheric electrode montage, there was a significant difference between the Fazekas 3 group and both the Fazekas 2 and Fazekas 0 groups in the M1 ROIs and on the whole brain level in the frontal-occipital setup (Supplementary Fig. S6, Supplementary Table 4.2). As a result, the variance of the electric field magnitude was likewise significantly decreased (Supplementary Fig. S9), which further affected the Sobol indices of several structures (skin, CSF, GM, Supplementary Fig. S12). Brain atrophy was determined a factor for decreased cortical electric field magnitude in transcranial electric stimulation in previous studies (Indahlastari et al., 2020, Woods et al., 2019, Mahdavi et al., 2018). Our subjects were aged matched to mitigate differences in cortical atrophy. For verification, we assessed the normalized volume of gray and white matter and the normalized total brain volume for each subject SIENAX (Smith et al., 2002) from FSL v6.0 (Smith et al., 2004) (see Supplementary Section S3). Indeed, there was no systematic decrease in brain volume with increasing lesion load, but the Fazekas 3 group had a significantly lowered total brain volume compared to the Fazekas 0 group. This difference was mainly driven by a significant decrease in white matter volume, possibly due to the advanced stage of the lesioned white matter. Yet, no significant correlation between total brain volume and the electric field magnitude could be found in any of the ROIs or electrode setups. Finally, despite the reduction in variance, the Sobol index of WMLs is still significantly higher in the Fazekas 3 group than all other groups on the whole-brain level, in the M1 ROIs, and the occipital electrode ROI, underlining the robustness of the reported effects.

By quantifying the contribution of the distinct tissue classes to the variance of the electric field magnitude due to their uncertainty in electrical conductivity, our results provide an estimate of the robustness of the results derived from tES simulations when choosing an arbitrary conductivity value from the analyzed ranges. In this study, the influence of uncertainty in the electrical conductivity of WML tissue was of particular interest. We consider the influence of the lesioned tissue on the electric field in Fazekas 1 and 2 subjects virtually negligible in superficial, cortical targets for both electrode configurations. No significant difference between the two groups was found and the WML Sobol index was, on average, approximately only a thousandth of that of the skin tissue class (Supplementary Tables S2.1 – S2.2). We found a similarly low influence in deeper targets. Thus, the primary concern for accurate, individualized tDCS simulations in these cases is the correct electrical conductivity of the skin, skull, and gray matter tissue, rather than that of deeper subcortical structures. Only the Fazekas 3 group showed a significantly higher contribution of WMLs to the total variance, an increased radius of WML influence around the lesioned tissue on the exemplary line plots, and a major contribution to the variance of the electric field magnitude by the WMLs on a single-subject level in the deeper regions of interest when the lesions were adjacent. For these subjects with a high lesion load, we recommend examining the location of the lesions. The exclusion of subjects with lesions close to the target site from individualized simulation-informed tDCS intervention studies should be considered while the actual conductivity value of WMLs remains unknown. Otherwise, the estimations from the simulated electric field should be regarded as less reliable.

In this study, we selected an electrode montage that is commonly used for motor cortex stimulation (Lindenberg et al., 2010, Mordillo-Mateos et al., 2012, Morya et al., 2019, Waters-Metenier et al., 2014), and a montage that reaches deeper cortical targets (Gomez-Tames et al., 2020) and has been used before to stimulate arousal (Mauri et al., 2015). Indeed, the spatially averaged mean electric field magnitudes in the deep ROIs were similar to averaged mean electric field magnitude at the mid-layer ROIs for the frontal-occipital electrode montage confirming the observations in (Gomez-Tames et al., 2020) (Supplementary Section S5.2.1, Supplementary tables S1.1 & S1.2). Both setups show the same trend of an increased but generally low influence of WMLs with increasing lesion load on a group level in cortical targets. We cannot directly infer whether the presented results hold for other electrode configurations. The comparison to results from (Saturnino et al., 2019) showed a high correspondence in the Sobol indices at the mid-layer despite differences in electrode montage (bihemispheric vs. unihemispheric) and age range (young vs. old). This may suggest that the Sobol indices are robust against different electrode positions. In addition, Indahlastari et al. (2021) find similarly the strongest changes rather localized within and around the WMLs. This may indicate that the reported effect is stable across electrode configurations and is rather dependent on the lesion location than on the electrode position. Given that we found further reduced WML Sobol indices on a group-level in the frontal-occipital setup compared to the bihemispheric configuration, the intensity of this effect might be modulated mildly by the electrode configuration but within a generally low range.

One limitation of the presented work might be that white matter anisotropy was not included in the physical properties of the head models because of lacking suitable diffusion-weighted imaging data. The imaging data were obtained from the existing pool of data of a large cross-sectional study, the LIFE-Adult study (Loeffler et al., 2015). Modeling studies have shown that the consideration of white matter anisotropy changes the electric field in simulations of tES (Miranda et al., 2003, Suh et al., 2012, Wagner et al., 2013, Shahid et al., 2014). Therefore, its role in the interplay of the electric field and white matter lesions is an important subject for further future investigations. Despite this lacking level of detail in modeling the physical properties of the white matter tissue, we consider the reported findings plausible and reliable. Huang et al. (2017) provide first evidence from intracranial recordings of three subjects that simulated electric fields from tES simulations with pure scalar elecftrical conductivity can achieve the same accuracy as fields from simulations with anisotropic white matter conductivity. Individually adjusted electrical conductivity values, so-called calibrated electrical conductivity values, for each subject were deemed of greater importance than modeling anisotropic white matter conductivity. Within the scope of our uncertainty analysis, a wide range of electrical conductivity values for each tissue class, including white matter, was covered. These ranges were selected to include the set of optimal conductivity values, which would yield the most accurately simulated electric field for each subject. Moreover, our analysis provides an assessment of the variance of the electric field due to non-optimally selected conductivity values complementing the concept of calibrated electrical conductivity by an estimate of the robustness of the simulated electric field magnitude.

To verify our entire workflow, we compared the previous work of Saturnino et al. (2019) to our results on a whole-brain level (Fig. 6, Fig. 7, Supplementary Figs. S4, S8, S11 and S12, orange dot). While the same framework for sensitivity analysis was used, the head modeling and simulation pipeline was entirely different. Despite these differences, the variance of the electric field and the Sobol indices of the healthy tissue reported in Saturnino et al. (2019) integrate well into our group results. Therefore, we consider our results comparable to previous work.

The presented results were obtained in participants with subcortical lesions of vascular origin. The electrical conductivity of these lesions was modeled highly uncertain in the sensitivity analysis. For this reason, these results might also apply to other patient populations with other types of subcortical lesions caused by diseases such as multiple sclerosis, infectious encephalitis, and leukodystrophy.

Our study systematically examined the influence of pathological brain structures on a group level in an uncertainty analysis. With 88 individual head models of both sexes, a wide range of phenotypes was covered. By leveraging a sensitivity analysis, the limitation of unknown conductivity of the structure under investigation, namely white matter lesions, was overcome. Our results support that white matter lesions must only be considered on an individual level in the case of a high lesion load and if the lesions occur in the vicinity of the stimulation site when conducting a tDCS intervention.

## Funding sources

B. Kalloch was supported by FAZIT Stiftung and the International Max Planck Research School on Neuroscience of Communication.

### CRediT authorship contribution statement

**Benjamin Kalloch:** Conceptualization, Formal analysis, Investigation, Methodology, Software, Validation, Visualization, Writing – original draft. **Konstantin Weise:** Conceptualization, Methodology, Software, Validation, Writing – review & editing. **Leonie Lampe:** Resources, Data curation, Writing – review & editing. **Pierre-Louis Bazin:** Methodology, Software, Writing – review & editing. **Arno Villringer:** Funding acquisition, Resources, Supervision, Writing – review & editing. **Mario Hlawitschka:** Conceptualization, Project administration, Supervision, Writing – review & editing. **Bernhard Sehm:** Conceptualization, Project administration, Supervision, Writing – review & editing.

## Declaration of Competing Interest

The authors declare that they have no known competing financial interests or personal relationships that could have appeared to influence the work reported in this paper.

## References

[b0005] J. Ahrens, B. Geveci, C. Law, Paraview: An end-user tool for large data visualization, *The visualization handbook,* vol. 717, 2005.

[b0010] Ammann C., Lindquist M.A., Celnik P.A. (2017). Response variability of different anodal transcranial direct current stimulation intensities across multiple sessions. Brain Stimul..

[b0015] Antonenko D., Thielscher A., Saturnino G.B., Aydin S., Ittermann B., Grittner U., Flöel A. (2019). Towards precise brain stimulation: is electric field simulation related to neuromodulation?. Brain Stimul..

[b0020] O.O. Awosika, L.G. Cohen, Transcranial direct current stimulation in stroke rehabilitation: present and future, in: *Practical Guide to Transcranial Direct Current Stimulation*, Springer, 2019, p. 509–539.

[b0025] Biou E., Cassoudesalle H., Cogné M., Sibon I., De Gabory I., Dehail P., Aupy J., Glize B. (2019). Transcranial direct current stimulation in post-stroke aphasia rehabilitation: a systematic review. Ann. Phys. Rehabil. Med..

[b0030] Blender Foundation, Blender, 2020.

[b0035] Bogovic J.A., Prince J.L., Bazin P.-L. (2013). A multiple object geometric deformable model for image segmentation. Comput. Vis. Image Underst..

[b0040] Chen L. (2004). Mesh smoothing schemes based on optimal delaunay triangulations. IMR.

[b0045] Cignoni P., Callieri M., Corsini M., Dellepiane M., Ganovelli F., Ranzuglia G. (2008). Eurographics Italian Chapter Conference.

[b0050] Crestaux T., Le Maıtre O., Martinez J.-M. (2009). Polynomial chaos expansion for sensitivity analysis. Reliab. Eng. Syst. Saf..

[b0055] Cruz Gonzalez P., Fong K.N.K., Chung R.C.K., Ting K.-H., Law L.L.F., Brown T. (2018). Can transcranial direct-current stimulation alone or combined with cognitive training be used as a clinical intervention to improve cognitive functioning in persons with mild cognitive impairment and dementia? A systematic review and meta-analysis. *Front. Human Neurosci.*.

[b0060] Datta A., Baker J.M., Bikson M., Fridriksson J. (2011). Individualized model predicts brain current flow during transcranial direct-current stimulation treatment in responsive stroke patient. Brain Stimul..

[b0065] David M.C.M.M., Moraes A.A.d., Costa M.L.d., Franco C.I.F. (2018). Transcranial direct current stimulation in the modulation of neuropathic pain: a systematic review. Neurol. Res..

[b0070] De Leeuw F.E., de Groot J.C., Achten E., Oudkerk M., Ramos L.M.P., Heijboer R., Hofman A., Jolles J., Van Gijn J., Breteler M.M.B. (2001). Prevalence of cerebral white matter lesions in elderly people: a population based magnetic resonance imaging study. The Rotterdam Scan Study. J. Neurol. Neurosurg. Psychiatry.

[b0075] Du Q., Faber V., Gunzburger M. (1999). Centroidal voronoi tessellations: applications and algorithms. SIAM Rev..

[b0080] Evans C., Bachmann C., Lee J.S.A., Gregoriou E., Ward N., Bestmann S. (2020). Dose-controlled tDCS reduces electric field intensity variability at a cortical target site. Brain Stimul..

[b0085] Fabri A., Pion S. (2009). *Proceedings of the 17th ACM SIGSPATIAL international conference on advances in geographic information systems*.

[b0090] Fazekas F., Chawluk J.B., Alavi A., Hurtig H.I., Zimmerman R.A. (1987). MR signal abnormalities at 1.5 T in Alzheimer's dementia and normal aging. Am. J. Roentgenol..

[b0095] Filmer H.L., Ehrhardt S.E., Shaw T.B., Mattingley J.B., Dux P.E. (2019). The efficacy of transcranial direct current stimulation to prefrontal areas is related to underlying cortical morphology. Neuroimage.

[b0100] Gomez-Tames J., Asai A., Hirata A. (2020). Significant group-level hotspots found in deep brain regions during transcranial direct current stimulation (tDCS): a computational analysis of electric fields. Clin. Neurophysiol..

[b0105] A. Habich, K. D. Fehér, D. Antonenko, C.-J. Boraxbekk, A. Flöel, C. Nissen, H. R. Siebner, A. Thielscher, S. Klöppel, Stimulating aged brains with transcranial direct current stimulation: opportunities and challenges, *Psychiatry Res.: Neuroimaging,* 111179, 2020. 10.1016/j.pscychresns.2020.111179 32972813

[b0110] Huang Y., Liu A.A., Lafon B., Friedman D., Dayan M., Wang X., Bikson M., Doyle W.K., Devinsky O., Parra L.C. (2017). Measurements and models of electric fields in the in vivo human brain during transcranial electric stimulation. Elife.

[b0115] Huntenburg J.M., Steele C.J., Bazin P.-L. (2018). Nighres: processing tools for high-resolution neuroimaging. GigaScience.

[b0120] Im C., Seo H., Jun S.C. (2018). *2018 40th Annual International Conference of the IEEE Engineering in Medicine and Biology Society (EMBC)*.

[b0125] Indahlastari A., Albizu A., O’Shea A., Forbes M.A., Nissim N.R., Kraft J.N., Evangelista N.D., Hausman H.K., Woods A.J., Initiative A.d.N. (2020). Modeling transcranial electrical stimulation in the aging brain. Brain Stimul..

[b0130] Indahlastari A., Albizu A., Boutzoukas E.M., O’Shea A., Woods A.J. (2021). White matter hyperintensities affect transcranial electrical stimulation in the aging brain. Brain Stimul..

[b0135] Jamil A., Batsikadze G., Kuo H.-I., Meesen R.L.J., Dechent P., Paulus W., Nitsche M.A. (2020). Current intensity-and polarity-specific online and aftereffects of transcranial direct current stimulation: An fMRI study. Hum. Brain Mapp..

[b0140] B. Kalloch, J. Bode, M. Kozlov, A. Pampel, M. Hlawitschka, B. Sehm, A. Villringer, H. E. Möller and P.-L. Bazin, Semi-automated generation of individual computational models of the human head and torso from MR images, *Magnetic Reson. Med.,* 2018. 10.1002/mrm.27508 30230021

[b0145] B. Kalloch, P.-L. Bazin, A. Villringer, B. Sehm and M. Hlawitschka, A flexible workflow for simulating transcranial electric stimulation in healthy and lesioned brains, *PLOS One,* 5 2020. 10.1371/journal.pone.0228119 PMC7224502 32407389

[b0150] Katoch N., Choi B.K., Sajib S.Z.K., Lee E., Kim H.J., Kwon O.I., Woo E.J. (2018). Conductivity tensor imaging of in vivo human brain and experimental validation using giant vesicle suspension. IEEE Trans. Med. Imaging.

[b0155] Kim J.-H., Kim D.-W., Chang W.-H., Kim Y.-H., Im C.-H. (2013). *2013 35th Annual International Conference of the IEEE Engineering in Medicine and Biology Society (EMBC)*.

[b0160] I. Laakso, S. Tanaka, M. Mikkonen, S. Koyama, A. Hirata, Variability in TDCS electric fields: Effects of electrode size and configuration, in *2017 XXXIInd General Assembly and Scientific Symposium of the International Union of Radio Science (URSI GASS)*, 2017.

[b0165] Laakso I., Tanaka S., Koyama S., De Santis V., Hirata A. (2015). Inter-subject variability in electric fields of motor cortical tDCS. Brain Stimul..

[b0170] Laakso I., Mikkonen M., Koyama S., Hirata A., Tanaka S. (2019). Can electric fields explain inter-individual variability in transcranial direct current stimulation of the motor cortex?. Sci. Rep..

[b0175] Lampe L., Kharabian-Masouleh S., Kynast J., Arelin K., Steele C.J., Löffler M., Witte A.V., Schroeter M.L., Villringer A., Bazin P.-L. (2019). Lesion location matters: the relationships between white matter hyperintensities on cognition in the healthy elderly. J. Cereb. Blood Flow Metab..

[b0180] Lee H.K., Ahn S.J., Shin Y.M., Kang N., Cauraugh J.H. (2019). Does transcranial direct current stimulation improve functional locomotion in people with Parkinson’s disease? a systematic review and meta-analysis. J. NeuroEng. Rehabil..

[b0185] Lindenberg R., Renga V., Zhu L.L., Nair D., Schlaug G.M.D.P. (2010). Bihemispheric brain stimulation facilitates motor recovery in chronic stroke patients. Neurology.

[b0190] Liu H., Yang Y., Xia Y., Zhu W., Leak R.K., Wei Z., Wang J., Hu X. (2017). Aging of cerebral white matter. Ageing Res. Rev..

[b0195] Loeffler M., Engel C., Ahnert P., Alfermann D., Arelin K., Baber R., Beutner F., Binder H., Brähler E., Burkhardt R. (2015). The LIFE-Adult-Study: objectives and design of a population-based cohort study with 10,000 deeply phenotyped adults in Germany. *BMC Public Health*.

[b0200] Lorensen W.E., Cline H.E. (1987). *ACM siggraph computer graphics*.

[b0205] Lucas B.C., Bogovic J.A., Carass A., Bazin P.-L., Prince J.L., Pham D.L., Landman B.A. (2010). The Java Image Science Toolkit (JIST) for rapid prototyping and publishing of neuroimaging software. Neuroinformatics.

[b0210] Mahdavi S., Towhidkhah F., Initiative A.d.N. (2018). Computational human head models of tDCS: influence of brain atrophy on current density distribution. Brain Stimul..

[b0215] Mauri P., Miniussi C., Balconi M., Brignani D. (2015). Bursts of transcranial electrical stimulation increase arousal in a continuous performance test. Neuropsychologia.

[b0220] Mcauliffe M., Lalonde F., McGarry D.P., Gandler W., Csaky K., Trus B. (2001). *Proceedings of the 14th IEEE Symposium on Computer-Based Medical Systems*.

[b0225] H.M. McCann, L. Beltrachini, Does participant_s age impact on tDCS induced fields? Insights from computational simulations, *Biomed. Phys. Eng. Exp.,* 2021. 10.1088/2057-1976/ac0547 34038881

[b0230] Minjoli S., Saturnino G.B., Blicher J.U., Stagg C.J., Siebner H.R., Antunes A., Thielscher A. (2017). The impact of large structural brain changes in chronic stroke patients on the electric field caused by transcranial brain stimulation. NeuroImage: Clinical.

[b0235] Miranda P.C., Hallett M., Basser P.J. (2003). The electric field induced in the brain by magnetic stimulation: a 3-D finite-element analysis of the effect of tissue heterogeneity and anisotropy. IEEE Trans. Biomed. Eng..

[b0240] Mordillo-Mateos L., Turpin-Fenoll L., Millán-Pascual J., Núñez-Pérez N., Panyavin I., Gómez-Argüelles J.M., Botia-Paniagua E., Foffani G., Lang N., Oliviero A. (2012). Effects of simultaneous bilateral tDCS of the human motor cortex. Brain Stimul..

[b0245] Morya E., Monte-Silva K., Bikson M., Esmaeilpour Z., Biazoli C.E., Fonseca A., Bocci T., Farzan F., Chatterjee R., Hausdorff J.M. (2019). Beyond the target area: an integrative view of tDCS-induced motor cortex modulation in patients and athletes. J. NeuroEng. Rehabil..

[b0250] Opitz A., Paulus W., Will S., Antunes A., Thielscher A. (2015). Determinants of the electric field during transcranial direct current stimulation. Neuroimage.

[b0255] Orrù G., Conversano C., Hitchcott P.K., Gemignani A. (2020). Motor stroke recovery after tDCS: a systematic review. Rev. Neurosci..

[b0260] Parazzini M., Fiocchi S., Cancelli A., Cottone C., Liorni I., Ravazzani P., Tecchio F. (2017). A computational model of the electric field distribution due to regional personalized or nonpersonalized electrodes to select transcranial electric stimulation target. IEEE Trans. Biomed. Eng..

[b0265] R Core Team, R: A Language and Environment for Statistical Computing, Vienna, 2020.

[b0270] Rorden C., Bonilha L., Fridriksson J., Bender B., Karnath H.-O. (2012). Age-specific CT and MRI templates for spatial normalization. Neuroimage.

[b0275] Sadleir R.J., Vannorsdall T.D., Schretlen D.J., Gordon B. (2010). Transcranial direct current stimulation (tDCS) in a realistic head model. Neuroimage.

[b0280] Salat D.H., Buckner R.L., Snyder A.Z., Greve D.N., Desikan R.S.R., Busa E., Morris J.C., Dale A.M., Fischl B. (2004). Thinning of the cerebral cortex in aging. Cereb. Cortex.

[b0285] Saturnino G.B., Thielscher A., Madsen K.H., Knösche T.R., Weise K. (2019). A principled approach to conductivity uncertainty analysis in electric field calculations. Neuroimage.

[b0290] Schmidt C., Wagner S., Burger M., van Rienen U., Wolters C.H. (2015). Impact of uncertain head tissue conductivity in the optimization of transcranial direct current stimulation for an auditory target. J. Neural Eng..

[b0295] Shahid S., Wen P., Ahfock T. (2011). *2011 IEEE/ICME International Conference on Complex Medical Engineering (CME)*.

[b0300] Shahid S., Wen P., Ahfock T. (2014). Assessment of electric field distribution in anisotropic cortical and subcortical regions under the influence of tDCS. Bioelectromagnetics.

[b0305] Shiee N., Bazin P.-L., Ozturk A., Reich D.S., Calabresi P.A., Pham D.L. (2010). A topology-preserving approach to the segmentation of brain images with multiple sclerosis lesions. NeuroImage.

[b0310] S. M. Smith, M. Jenkinson, M. W. Woolrich, C. F. Beckmann, T. E. J. Behrens, H. Johansen-Berg, P. R. Bannister, M. De Luca, I. Drobnjak, D. E. Flitney et al., Advances in functional and structural MR image analysis and implementation as FSL, *Neuroimage,* 23, S208–S219, 2004. 10.1016/j.neuroimage.2004.07.051 15501092

[b0315] Smith S.M., Zhang Y., Jenkinson M., Chen J., Matthews P.M., Federico A., De Stefano N. (2002). Accurate, robust, and automated longitudinal and cross-sectional brain change analysis. Neuroimage.

[b0320] Suh H.S., Lee W.H., Kim T.-S. (2012). Influence of anisotropic conductivity in the skull and white matter on transcranial direct current stimulation via an anatomically realistic finite element head model. Phys. Med. Biol..

[b0325] Summers J.J., Kang N., Cauraugh J.H. (2016). Does transcranial direct current stimulation enhance cognitive and motor functions in the ageing brain? a systematic review and meta-analysis. Ageing Res. Rev..

[b0330] Taubin G. (1995). *Proceedings of the 22nd annual conference on Computer graphics and interactive techniques*.

[b0335] The OpenFOAM Foundation, OpenFOAM, 2020.

[b0340] M. Tomczak, E. Tomczak, The need to report effect size estimates revisited. An overview of some recommended measures of effect size, 2014.

[b0345] van Asseldonk E.H.F., Boonstra T.A. (2016). Transcranial direct current stimulation of the leg motor cortex enhances coordinated motor output during walking with a large inter-individual variability. Brain Stimul..

[b0350] Waehnert M.D., Dinse J., Weiss M., Streicher M.N., Waehnert P., Geyer S., Turner R., Bazin P.-L. (2014). Anatomically motivated modeling of cortical laminae. Neuroimage.

[b0355] Wagner T., Fregni F., Fecteau S., Grodzinsky A., Zahn M., Pascual-Leone A. (2007). Transcranial direct current stimulation: a computer-based human model study. Neuroimage.

[b0360] Wagner S., Rampersad S.M., Aydin Ü., Vorwerk J., Oostendorp T.F., Neuling T., Herrmann C.S., Stegeman D.F., Wolters C.H. (2013). Investigation of tDCS volume conduction effects in a highly realistic head model. J. Neural Eng..

[b0365] Warfield S.K., Zou K.H., Wells W.M. (2004). Simultaneous truth and performance level estimation (STAPLE): an algorithm for the validation of image segmentation. IEEE Trans. Med. Imaging.

[b0370] Waters-Metenier S., Husain M., Wiestler T., Diedrichsen J. (2014). Bihemispheric transcranial direct current stimulation enhances effector-independent representations of motor synergy and sequence learning. J. Neurosci..

[b0375] Weise K., Poßner L., Müller E., Gast R., Knösche T.R. (2020). Pygpc: a sensitivity and uncertainty analysis toolbox for Python. SoftwareX.

[b0380] Wickham H. (2016).

[b0385] A. J. Woods, D. Antonenko, A. Flöel, B. M. Hampstead, D. Clark, H. Knotkova, Transcranial direct current stimulation in aging research, in *Practical Guide to Transcranial Direct Current Stimulation*, Springer, 2019, p. 569–595.

