# Supplementary material for: The influence of white matter lesions on the electric field in transcranial electric stimulation
Source: Neuroimage Clin. 2022 Jun 2;35:103071. doi: 10.1016/j.nicl.2022.103071 (PMC9168230; doi:10.1016/j.nicl.2022.103071)
Supplement: Supplementary data 1 [file mmc1.docx]

The influence of white matter lesions on the electric field in transcranial electric stimulation

**Benjamin Kalloch^1,2,3,4^, Konstantin Weise^3,5^, Leonie Lampe^1^, Pierre-Louis Bazin^1,6^, Arno Villringer^1^, Mario Hlawitschka^2^, Bernhard Sehm^1,7^**

*1 Max Planck Institute for Human Cognitive and Brain Sciences, Department of Neurology, Leipzig, Germany
2 Leipzig University of Applied Science, Faculty of Computer Science and Media, Leipzig, Germany
3 Max Planck Institute for Human Cognitive and Brain Sciences, Methods and Development Group “Brain Networks”, Leipzig, Germany
4 Technische Universität Ilmenau, Instiute of Biomedical Engineering and Informatics, Ilmenau, Germany
5 Technische Universität Ilmenau, Advanced Electromagnetics Group, Ilmenau, Germany
6 University of Amsterdam, Faculty of Social and Behavioural Sciences, Amsterdam, The Netherlands
7 Department of Neurology, Martin Luther University of Halle-Wittenberg, Germany*

- Supplementary Material -

Content

[S1 MRI acquisition parameters 2](#_Toc102414160)

[S2 Visualization of the lesion load in single subjects 3](#_Toc102414161)

[S3 Excluding brain tissue atrophy as influencing factor 4](#_Toc102414162)

[S4 Mid-layer interpolation 6](#_Toc102414163)

[S5 Region of interest analyses 10](#_Toc102414164)

[S5.1 Definition and creation of the regions of interest 10](#_Toc102414165)

[S5.2 Region of interest-specific group results 13](#_Toc102414166)

[S5.2.1 Mean electric field magnitude 13](#_Toc102414167)

[S5.2.2 Variance of the mean electric field magnitude 21](#_Toc102414168)

[S5.2.3 Sobol indices 27](#_Toc102414169)

[S6 Development of the analyzed result quantities across the head model volume, single-subject 36](#_Toc102414170)

[S7 Supplementary Tables 41](#_Toc102414171)

# S1 MRI acquisition parameters

The MPRAGE acquisition parameters were adjusted according to a standard protocol: flip angle 9°, repetition time 2300 ms, inversion time 900 ms, echo time 2.98 ms, 1 mm isotropic resolution, acquisition time 5.1 min. The parameters of the FLAIR image were standardized as well: repetition time 5000 ms, inversion time 1800 ms, echo time 395 ms, 1 mm isotropic resolution, acquisition time 7.02 min.

# S2 Visualization of the lesion load in single subjects


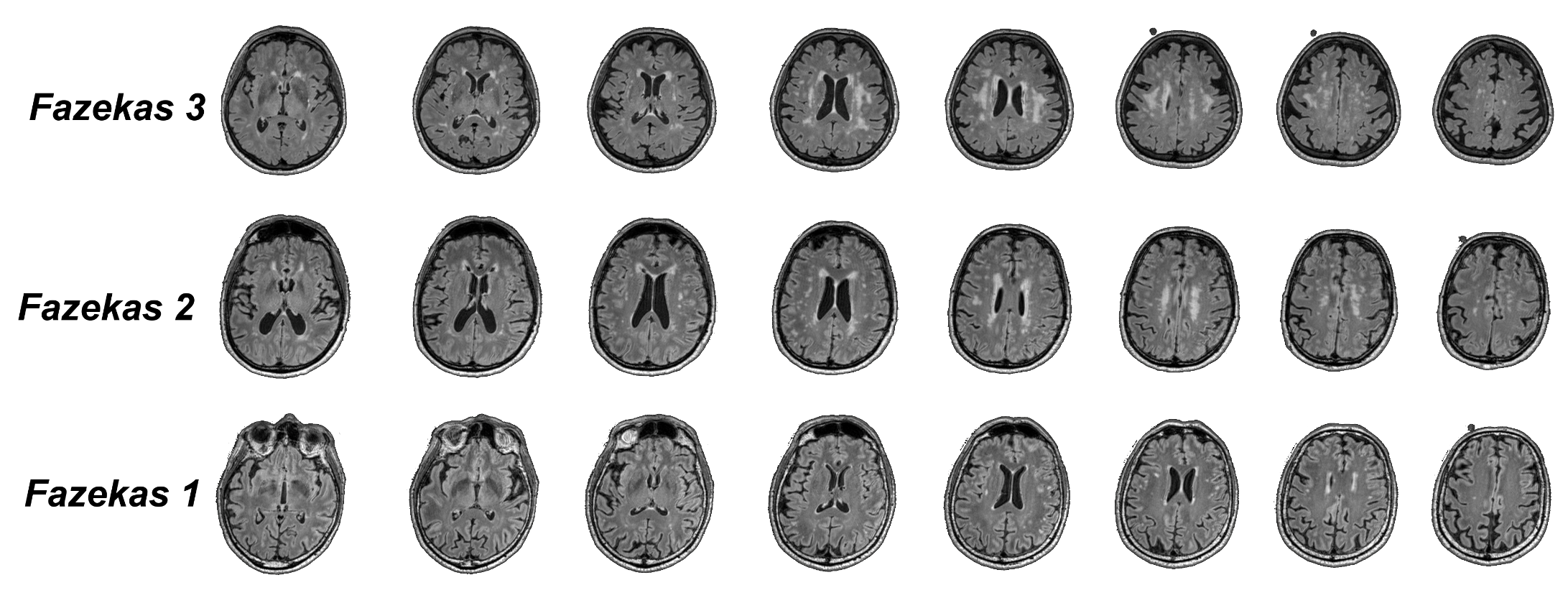


**Supplementary Figure S1** Example MR images of subjects from each analyzed group. *T2-FLAIR magnetic resonance image of single representative subjects of each group are displayed. White matter lesions appear as hyperintensities.*

# S3 Excluding brain tissue atrophy as influencing factor

The normalized cortical volume (i.e. gray matter volume), the volume of the white matter, and the entire brain was assessed from the T1-weighted head images of each subject using the tool SIENAX^[[1]](#footnote-1)^ from the FMRIB Software Library v6.0^[[2]](#footnote-2)^ (Supplementary Figure S2). The normally distributed samples were analyzed for significant differences at a significance level of $\alpha\leq0.05$using a one-way ANOVA with $\eta^{2}$ as the effect size measure and paired-samples t-tests with Bonferroni correction as post-hoc tests in R v. 3.4.4. The cortical volume (gmv), the white matter volume (wmv), and the total brain volume (bv) were significantly different between groups (gmv: $p=0.03m \eta^{2}=.088$, wmv: $p=0.002, \eta^{2}=.159$, bv: $p=0.005, \eta^{2}=.168$). Paired-samples t-tests indicated that the difference in cortical volume was mainly driven by a decrease in cortical thickness in the Fazkes 1 group (gmv F0-F1: $p=0.0$19). The difference in white matter volume can be explained by a significant reduction of white matter volume in the Fazekas 3 group compared to the Fazekas 0 group (wmv F0-F3: $p=0.0016$) and the Fazekas 1 and 0 groups (wmv F0-F1: $p=0.0388$). The difference in total brain volume is significant between the Fazekas 0 and Fazekas 1 group (bv F0-F1: $p=.0064$) as well as the Fazekas 0 and Fazekas 3 group (bv F0-F3: $p=.0192$). There is no systematic decrease in cortical or total brain volume with increasing Fazekas score.

In addition, the Kendall-tau rank correlation coefficient between the total brain volume and electric field magnitude in each region of interest and each electrode setup was determined. No significant correlation was found (Supplementary Table S1).


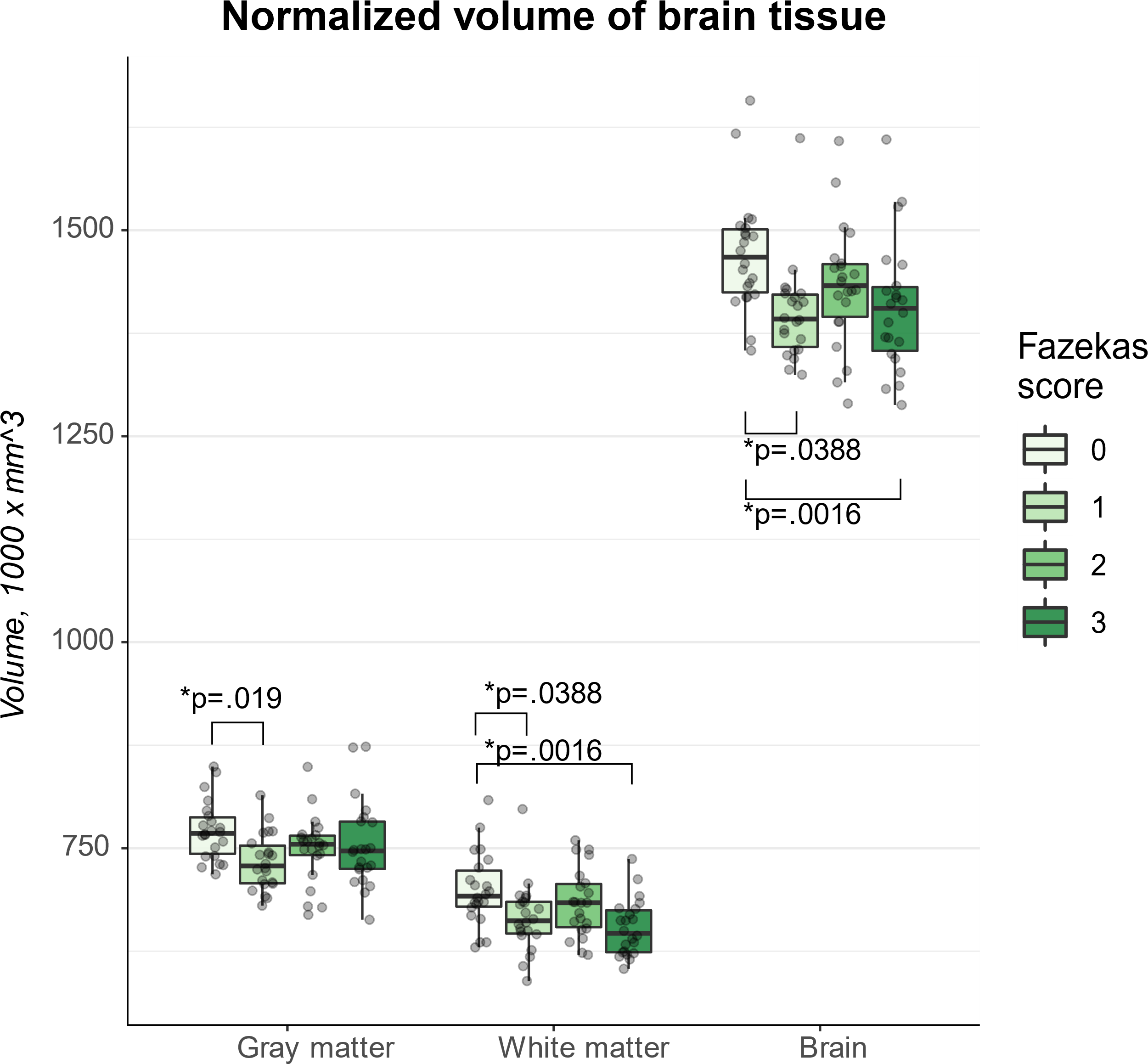


**Supplementary Figure S2** Normalized volume measurements of brain tissue. SIENAX from FSL was used to measure the normalized volume of gray matter, white matter and both combined.

|  | **Bihemispheric setup** | **Frontal-occipital setup** |
| --- | --- | --- |
| *M1 ROI left* | $\tau=-0.011, p=.891$ | $\tau=0.105, p=.197$ |
| *M1 ROI right* | $\tau=0.04, p=.625$ | $\tau=0.05, p=.521$ |
| *Electrode ROI left* | $\tau=-0.021, p=.801$ | $\tau=-0.024, p=.766$ |
| *Electrode ROI right* | $\tau=0.018, p=.825$ | $\tau=0.023, p=.778$ |
| *Wholebrain ROI* | $\tau=0.014, p=.861$ | $\tau=0.076, p=.348$ |
| *Hippocampus left* | $\tau=-0.004, p=.957$ | $\tau=0.018, p=.825$ |
| *Hippocampus right* | $\tau=-0.055, p=.497$ | $\tau=0.019, p=.819$ |
| *Thalamus left* | $\tau=-0.007, p=.927$ | $\tau=0.059, p=.468$ |
| *Thalamus right* | $\tau=-0.012, p=.885$ | $\tau=0.056, p=.492$ |

**Supplementary Table S1** Kendall-tau rank correlation coefficient and corresponding p-values between the total brain volume and the electric field magnitude in each region of interest and electrode setup.

# S4 Mid-layer interpolation

The sensitivity analysis was performed at the cortical mid-layer, which was considered the target site for tDCS. The mid-layer surface was created independently of the head volume mesh. It was, thus, not part of the geometry of the tetrahedral head volume meshes. This necessitated interpolating the cell-based result field of the numerical simulation, that is, the electric field magnitude, from the head volume mesh onto the nodes of the mid-layer surface. In our case, the vectorial electric field $\boldsymbol{E}$ as the gradient of the electric potential $\varphi$, $\boldsymbol{E}=-\nabla\varphi$, was first discretized at the center of each tetrahedral element of the computational grid using a least-squares gradient scheme by our FVM-based solver application. The subsequent interpolation onto the mid-layer involved a two-stage scheme. First, the magnitude of the electric field was computed and interpolated from the center of each cell of the head volume mesh to the vertices of that respective cell as follows. For each vertex of the mesh, the values from the centers of all adjacent cells were averaged. Large heterogeneity in the cell volumes of the adjacent cells may decrease the accuracy of this interpolation method. However, for the creation of our head volume meshes, no adaptive element size was used and element sizes were small, generally below $1 mm^{3}$ (mean volume: $0.72 mm^{3}$, sd: $0.155 mm^{3}$), diminishing this possible drawback. The described interpolation scheme is implemented in the VTK (Kitware Inc.) class vtkCellDataToPointData. Next, for each node of the mid-layer surface, the containing cell of the head mesh was determined. Finally, the electric field magnitude at the vertices of the containing cell were interpolated onto the mid-layer node by a weighted, linear interpolation. The weights were obtained from the barycentric coordinates of the mid-layer node within its containing cell of the head mesh. Locating the containing cell and determining barycentric coordinates were realized by the FindCell function of the VTK (Kitware Inc.) class vtkDataSet.

While this kind of linear interpolation scheme might introduce an unwanted smoothing of the simulations results^[[3]](#footnote-3)^, we expect, in our case, a comparably minor effect due to 1) the small element size of typically below$1 mm^{3}$ and 2) performing this operation in local mesh neighborhoods within the gray matter mesh compartment with homogenous electrical conductivity and without major jumps in the electric field magnitude in neighboring cells.

To provide an estimate of the smoothing of the electric field magnitude by the outlined interpolation scheme, it was performed for the cell centers (instead of the mid-layer nodes). The simulation-derived electric field magnitude was first interpolated (using vtkCellDataToPointData) to the vertices of the cells containing a mid-layer node as described before. From those vertices, the electric field magnitude was then interpolated back to the centers of the cells by the same weighted (by barycentric coordinates) linear interpolation as used for the mid-layer interpolation. The relative difference between the interpolated electric field magnitude at the cell centers and the original simulation-derived electric field magnitude at the cell centers was assessed for each node of the mid-layer as $\frac{\left| \left| E \right| \right|_{interpolated}-\left| \left| E \right| \right|_{orig}}{\left| \left| E \right| \right|_{orig}}$. The average unsigned relative difference was 3.44 % (SD: 4.5 %), with elevated differences peaking at the 99^th^ percentile of 21.13 % at sparse locations, where a mid-layer node was located within a tetrahedron at the outer or inner boundary of the gray matter mesh compartment (Supplementary Figure S3). We discovered such elevated levels of the relative interpolation error at the sulcal walls, where the gray matter mesh compartment was found to be particularly thin. As the electric field magnitude was commonly small at the sulcal walls the absolute error (as listed in Supplementary Table S2) was likewise small.
To confirm this estimate, the relative difference between the interpolated electric field magnitude at each node of the mid-layer and the value of the electric field magnitude at the centers of its containing cell was computed. The mean of the unsigned relative difference across all nodes of the mid-layer and the values at the center of their containing cells was well within the error bound with 3.43 % (SD: 4.34%). The 99^th^ percentile peak difference was 18.49 %.

| $\bar{\left\vert\left\vert\left\vert E \right\vert\right\vert_{interpolated}-\left\vert\left\vert E \right\vert\right\vert_{orig} \right\vert}$*in V/m* | | **Wholebrain** | **M1 left** | **M1 right** | **Electrode ROI left** | **Electrode ROI right** |
| --- | --- | --- | --- | --- | --- | --- |
| **C3-C4** | ***Fazekas 0*** | .0042  *sd*: 0.0059 | .0075  *sd*: 0.0089 | .0082  *sd*: 0.0104 | .0051  *sd*: 0.0071 | .0048  *sd*: 0.0065 |
|  | ***Fazekas 1*** | .003  *sd*: .0038 | .005  *sd*: .0057 | .0055  *sd*: .0063 | .0039  *sd*: .0048 | .0037  *sd*: .0046 |
|  | ***Fazekas 2*** | .0025  *sd*: .0036 | .0046  *sd*: .0057 | .0046  *sd*: .0057 | .0034  *sd*: .0048 | .0032  *sd*: .0044 |
|  | ***Fazekas 3*** | .002  *sd*: .0026 | .0028  *sd*: .0033 | .0031  *sd*: .004 | .0027  *sd*: .0038 | .0026  *sd*: .0035 |
| **OZ-FPZ** | ***Fazekas 0*** | .0047 *sd*: .0060 | .0064 *sd*: .0063 | .0062  *sd*: .0061 | .0045  *sd*: .006 | .0056  *sd*: .0075 |
|  | ***Fazekas 1*** | .0026  *sd*: .0037 | .0027  *sd*: .0031 | .0027  *sd*: .003 | .0025  *sd*: .0036 | .0037  *sd*: .0058 |
|  | ***Fazekas 2*** | .0027  *sd*: .0038 | .0032  *sd*: .0036 | .0033  *sd*: .0037 | .0027  *sd*: .0037 | .0039  *sd*: .006 |
|  | ***Fazekas 3*** | .0021  *sd*: .003 | .0017  *sd*: .0021 | .0015  *sd*: .0018 | .0021  *sd*: .0029 | .003  *sd*: .0045 |

**Supplementary Table** **S2** Group average of the absolute interpolation error in the mid-layer regions of interest per group and electrode setup.

We conclude from these analyses that the interpolated electric field values and the simulated electric field values are generally in close range. Therefore, we deem this error due to the interpolation negligible.


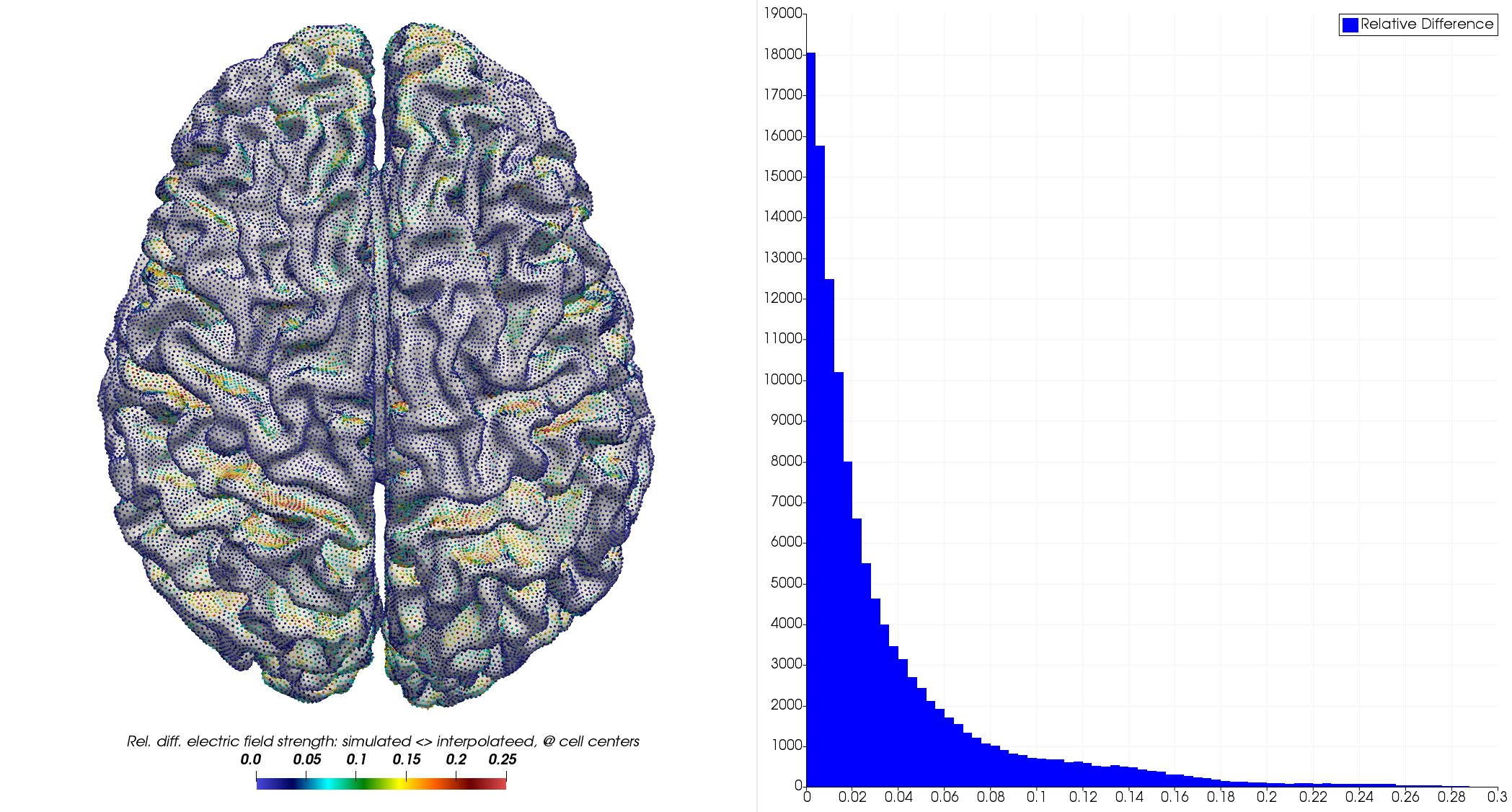


**Supplementary Figure S3** Estimation of the interpolation error depicted as equally sized spherical glyphs at the nodes of the mid-layer surface. *For the error estimation, the relative difference between the simulated electric field and the interpolated electric field at the centers of the cells of the head volume mesh containing a mid-layer node was computed. The unsigned relative difference is visualized as a spherical glyph positioned at the corresponding mid-layer node. Its color represents the absolute value of the relative difference.*

# S5 Region of interest analyses

Results were analyzed in four locations on the cortical mid-layer and in four deep structures as regions of interest. Their creation and the results obtained from these regions is described in this section.

## S5.1 Definition and creation of the regions of interest

To investigate the cortical area directly underneath the stimulating electrodes, the first pair of the mid-layer ROIs were defined as spheres of 5 cm diameter at the individual centers of the electrodes (“Electrode ROI left/right”, “Electrode ROI frontal/occipital”). The second pair of the mid-layer ROIs was located at the area of the upper extremity representation within the primary motor cortices on each hemisphere, representing the expected target stimulation sites for the bihemispheric montage (“M1 ROI left/right”). The thalamus (TH) and hippocampus (HPC) on both hemispheres constituted the four deep ROIs. Since the thalamus is positioned close to the ventricles, as are the periventricular white matter lesions, their increased influence in that region would be feasible. The hippocampus was selected as we found an association of specific locations of white matter hyperintensities and impaired memory function in previous work^[[4]](#footnote-4)^, possibly indicating an influence of WMLs on the hippocampus. Both deep structures were segmented by the employed brain segmentation algorithm MGDM. The data was extracted from the described ROIs using ParaView 5.6.3. The regions of interest are visualized in Supplementary Figure S4. Refer to Supplementary Table S3 for a summary of the extents of each ROI and their enclosed data points.


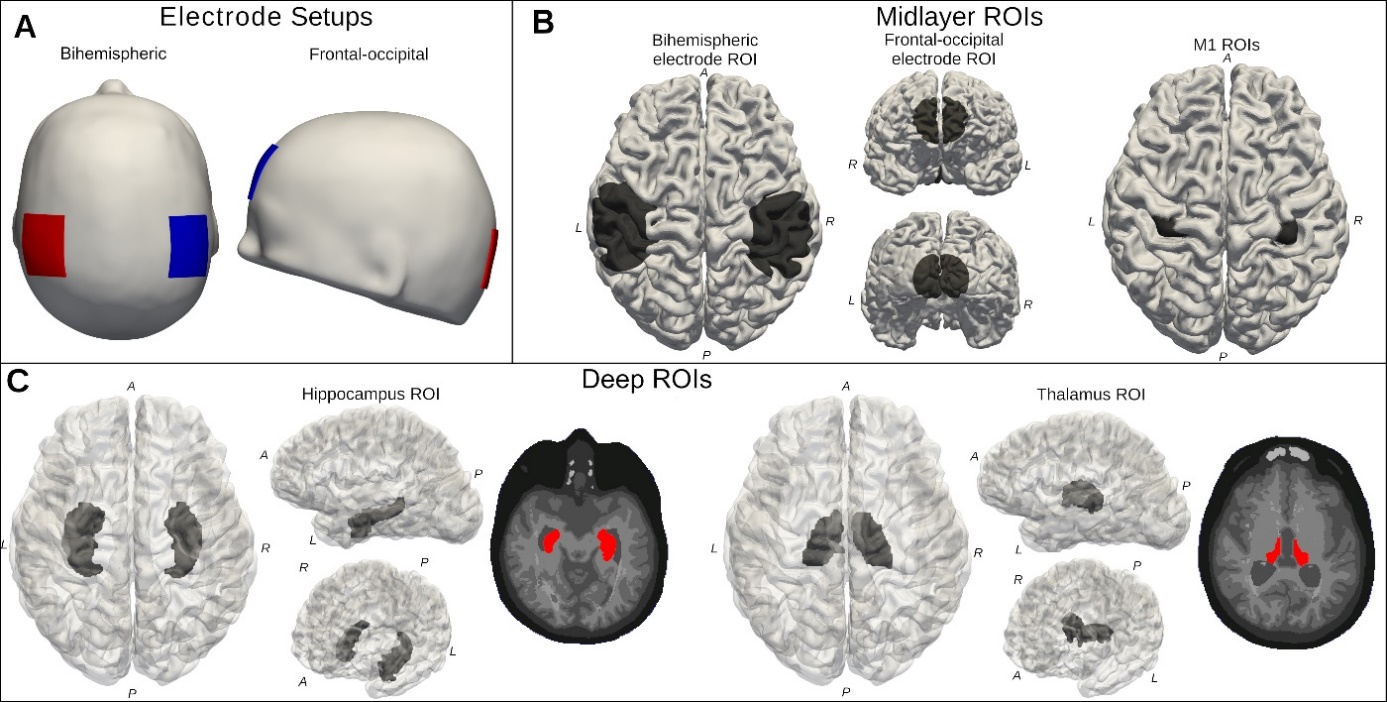


**Supplementary Figure S4** Illustration of the electrode setup and the regions of interest. (A) *All simulations were conducted using a bihemispheric electrode setup over the 10-20 coordinates C3 & C4 and a frontal-occipital setup over the coordinates FPZ & OZ. The regions of interest (ROIs) used for statistical analysis were defined on the cortical mid-layer (B) and in two deep regions (C). They are highlighted in dark gray in this illustration. The “Electrode ROI” was defined as the area underneath the electrode using a 5 cm sphere with the origin at the center of the electrode projected onto the mid-layer surface.* *A 3 cm sphere masked by the pre-central gyrus representation of the Brainnetome atlas (Fan et al., Cerebral Cortex, 2016) at the coordinate of the upper extremities representation within the primary motor cortex from the Human Motor Area Template (Mayka et al., NeuroImage, 2006) on the cortical mid-layer constituted the* “*M1 ROI*”*. The hippocampus and the thalamus (of both hemispheres) were selected as the deep ROIs. Their shape, location and volume were determined by the MGDM (Bogovic et al., Computer Vision and Image Understanding, 2013) brain segmentation algorithm.*

| **Region of interest type** | **Number of enclosed data points** | **Area/volume covered by region of interest** |
| --- | --- | --- |
| Electrode ROI frontal/occipital | 2978 +/- 409 | 4126 mm^2^ +/- 413 mm^2^ |
| Electrode ROI left/right | 4060 +/- 565 | 5465 mm^2^ +/- 734mm^2^ |
| M1 ROI left/right | 335 +/- 78 | 380 mm^2^ +/- 94 mm^2^ |
| Wholebrain ROI | 112,156 +/- 11067 | 1404 cm^2^ +/- 140 cm^2^ |
| HPC ROI | 7545 +/- 2641 | 4630 mm^3^ +/- 956 mm^3^ |
| TH ROI  **Supplementary Table S3** Characteristics of the regions of interest. *The number of enclosed data points, which were considered in the analysis, as well as the area (for the ROIs on the mid-layer surface) or the volume (for the volumetric, deep ROIs). Abbreviations: HPC = Hippocampus, TH = Thalamus* | 9015 +/- 2826 | 6153 mm^3^ +/- 1926 mm^3^ |

The electrode ROIs at the mid-layer were created individually for each subject according to the position of their electrodes separately for each electrode montage using a Python script for Paraview’s “Programmable Filter”. The center of gravity of the electrode was perpendicularly projected onto the cortex. For the frontal occipital setup, the projection occurred onto the convex hull of the cortex. Using the convex hull was necessary because of the central position of the electrodes at the longitudinal fissure. Without the convex hull, the center of gravity of the electrodes would have been projected deep into the brain through the longitudinal fissure onto the corpus callosum instead of directly underneath the electrode. The resulting coordinate constituted the center of a 5 cm diameter sphere used to clip the mid-layer surface. The values of the clipped mid-layer were further analyzed.

The M1 ROIs were first defined in MNI space. The respective coordinate of the upper extremity representation area within the primary motor cortex (M1) on the left hemisphere in MNI space (left M1 ROI, x = -37, y = -25, z = 64) was taken from the human motor area template^[[5]](#footnote-5)^ and symmetrically mirrored to the right hemisphere (right M1 ROI, x=37, y = -25, z = 64). A sphere of 3 cm diameter was created at these locations using a spherical kernel of 15 mm in fslmaths. The sphere was subsequently clipped with the representations of the precentral gyri of both hemispheres from the Brainnetome atlas^[[6]](#footnote-6)^ (labels: 53 – 64). Both ROIs were transformed into the subject space using the Advanced Normalization Tools (ANTS) v2.3.1^[[7]](#footnote-7)^ and the T1-weighted image of each subject. The “Resample With Dataset” filter of ParaView was used to transfer the ROIs in subject space onto the cortical mid-layer. Finally, values outside the ROI on the mid-layer were discarded, that is, multiplied by zero, and the remaining values were analyzed.
From the individual MGDM brain segmentation of each subject, the segments of both thalami and hippocampi were extracted and post-processed by image-morphological operations (opening and closing with a 3 mm spherical kernel each to smooth their boundary) in MIPAV. The resulting segmentation images were used to identify cells of the individual head models inside those deep regions in ParaView using the “ResampleWithDataset”-filter.

## S5.2 Region of interest-specific group results

In this section, the group-level result quantities of the uncertainty analysis are reported an plotted for each region of interest and each electrode setup (Supplementary Tables 1.1 & 1.2). Those result quantities are the mean electric field magnitude, mean( $\left\| \boldsymbol{E} \right\|$) (Supplementary Figs. S5 – S7), its variance, var( $\left\| \boldsymbol{E} \right\|$)) (Supplementary Figs. S8 – S10), due to the uncertainty in the electrical conductivity, and the decomposition of this variance into the contribution of each tissue type by means of so-called Sobol indices (Supplementary Figs. S11 – S13).

### S5.2.1 Mean electric field magnitude

The deep ROIs received a higher mean( $\left\| \boldsymbol{E} \right\|$) using the frontal-occipital setup than in the bihemispheric condition. The mean( $\left\| \boldsymbol{E} \right\|$) in all deep ROIs were similar to the mid-layer ROIs when using the frontal-occipital setup. For the deep ROIs under both electrode montages and the mid-layer ROIs in the bihemispheric condition, no significant differences in mean( $\left\| \boldsymbol{E} \right\|$) could be identified between Fazekas groups (Supplementary Tables S3.1 & S3.2). However, in the mid-layer ROIs under the frontal-occipital stimulation setup, there was a significant difference in the mean( $\left\| \boldsymbol{E} \right\|$) between groups on a whole-brain level and the M1 ROIs (Supplementary Table S3.1, Supplementary Figure S6, whole-brain: $p=.001, \eta^{2}=.152,$ M1 left: $p\ll.001, \eta^{2}=.198$, M1 right$: p\ll.001, \eta^{2}=.193$).


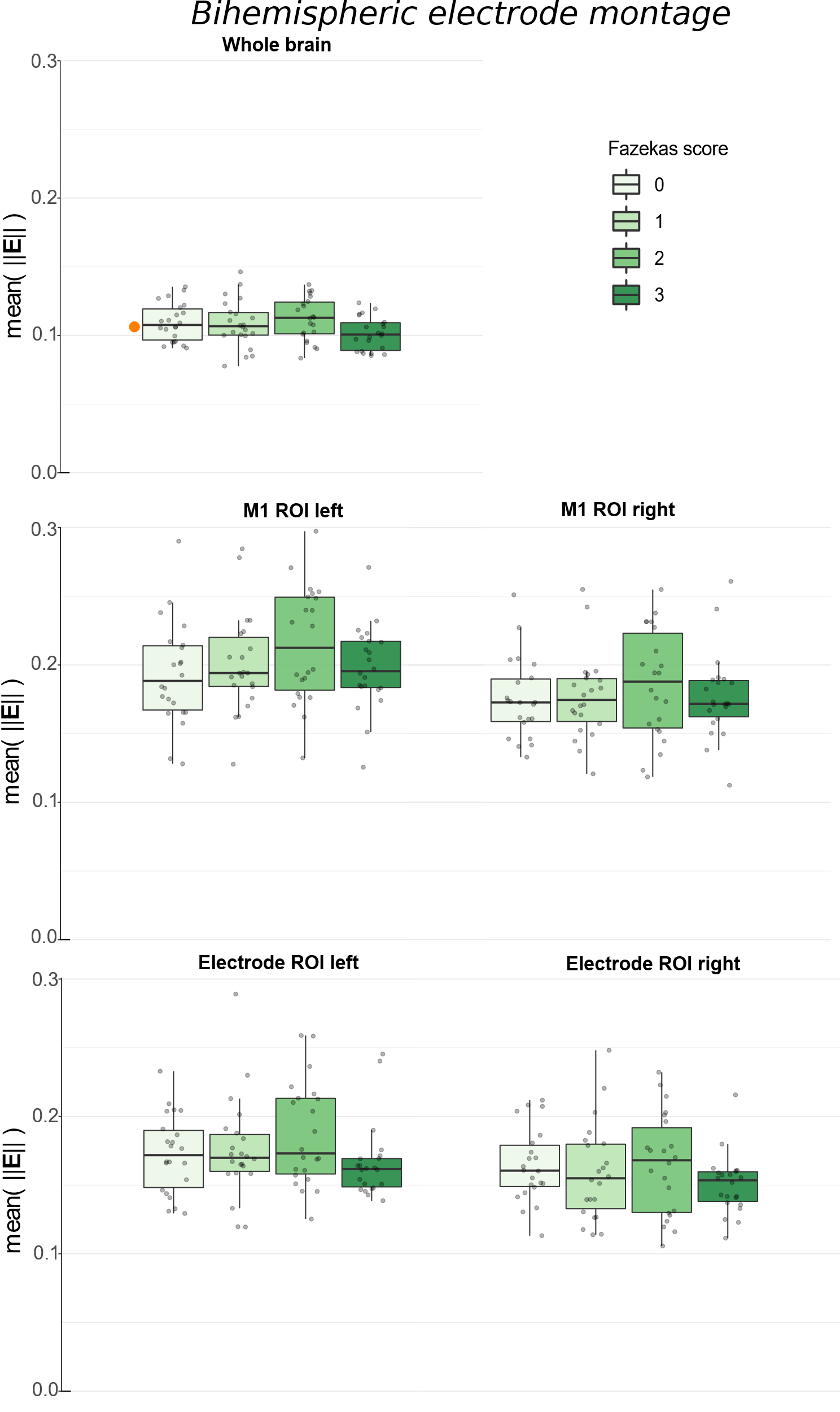


**Supplementary Figure S5** Group-wise boxplots of the mean electric field magnitude with the bihemispheric electrode montage. *Values were averaged within the mid-layer regions of interest and on a whole-brain level for every subject (represented as individual dots). Boxplots provide a group comparison. For comparison, the average* mean *electric field magnitude on a whole-brain level from the uncertainty analysis of a young adult* *(Saturnino et al., Neuroimage, 2019) was marked with an orange dot within the scatter-plot data of the Fazekas 0 group.*


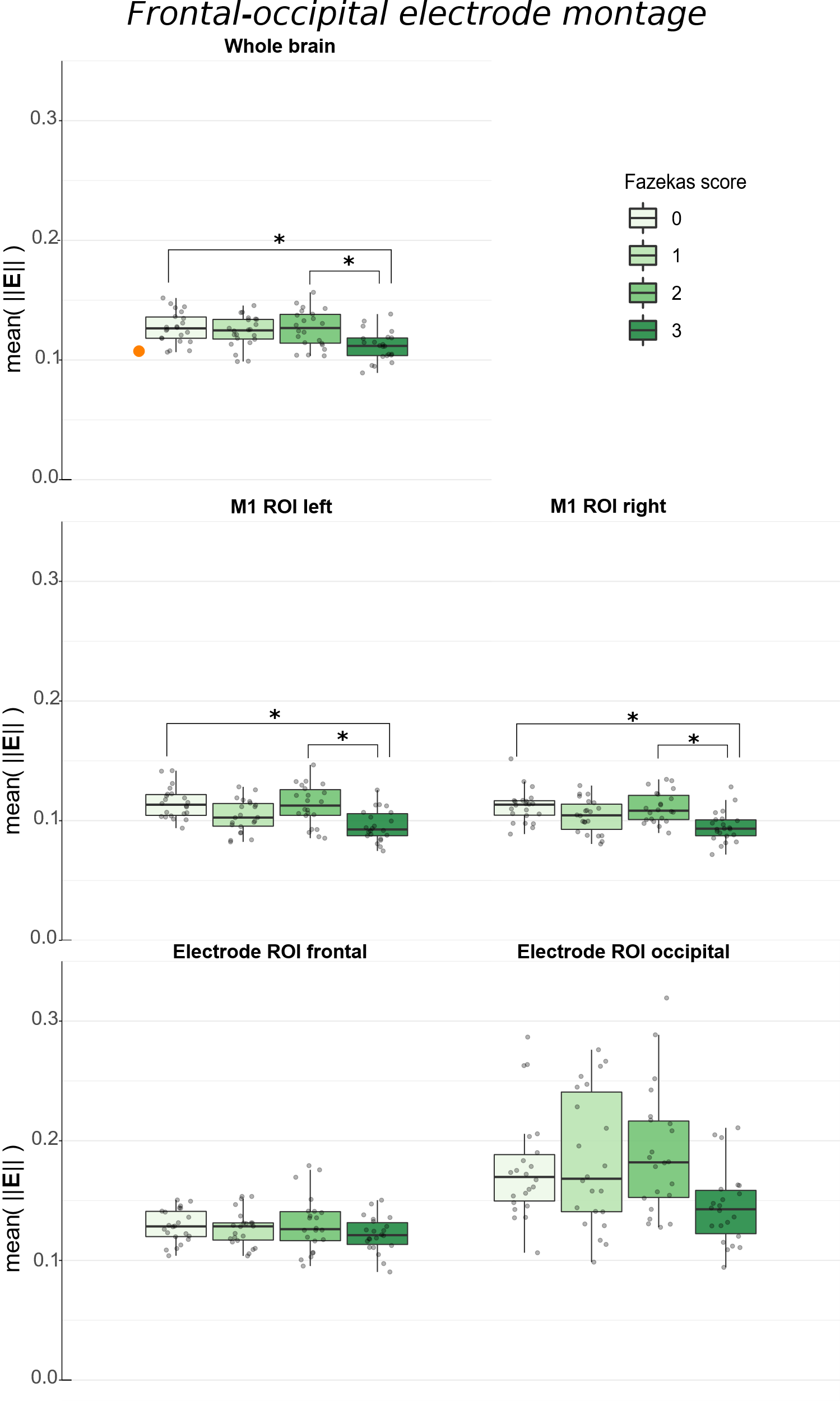


**Supplementary Figure S6** Group-wise boxplots of the mean electric field magnitude with the frontal (FPZ)-occipital (OZ) electrode montage. *Values were averaged within the mid-layer regions of interest and on a whole-brain level for every subject (represented as individual dots). Boxplots provide a group comparison. For comparison, the average* mean *electric field magnitude on a whole-brain level from the uncertainty analysis of a young adult (Saturnino et al., Neuroimage, 2019) was marked with an orange dot within the scatter-plot data of the Fazekas 0 group.*


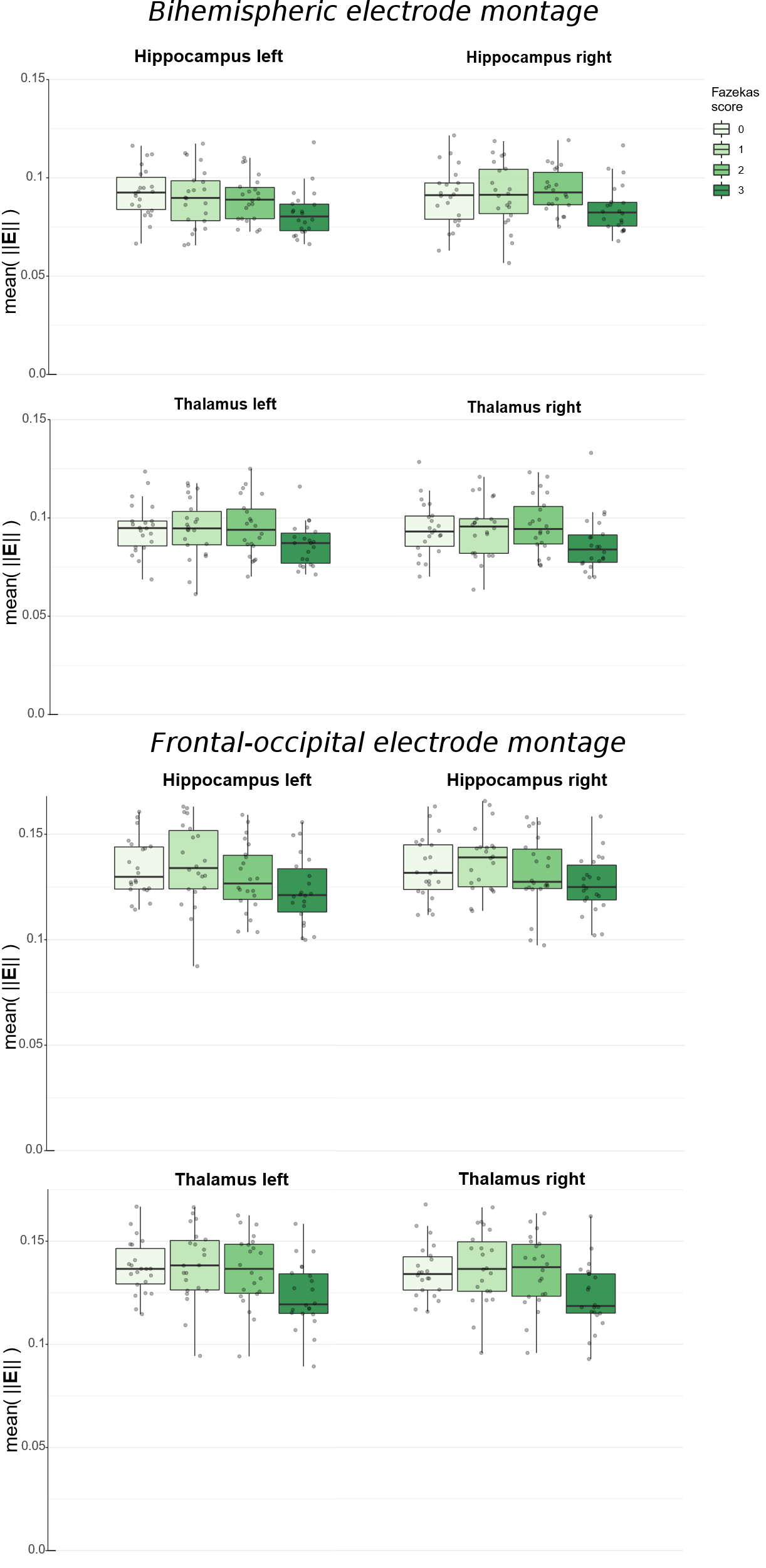


**Supplementary Figure S7** Group-wise boxplots of the mean electric field magnitude within the deep ROIs with both electrode montages. *Values were averaged within the deep regions of interest for every subject (represented as individual dots). Boxplots provide a group comparison.*

### S5.2.2 Variance of the mean electric field magnitude

The variance of the electric field magnitude differed significantly between Fazekas groups in the M1 ROIs when using the frontal-occipital electrode setup (Supplementary Table S3.1 and Supplementary Figure S9, M1 left: $p\ll.001, \eta^{2}=.27$, M1 right$: p\ll.001, \eta^{2}=.224$).


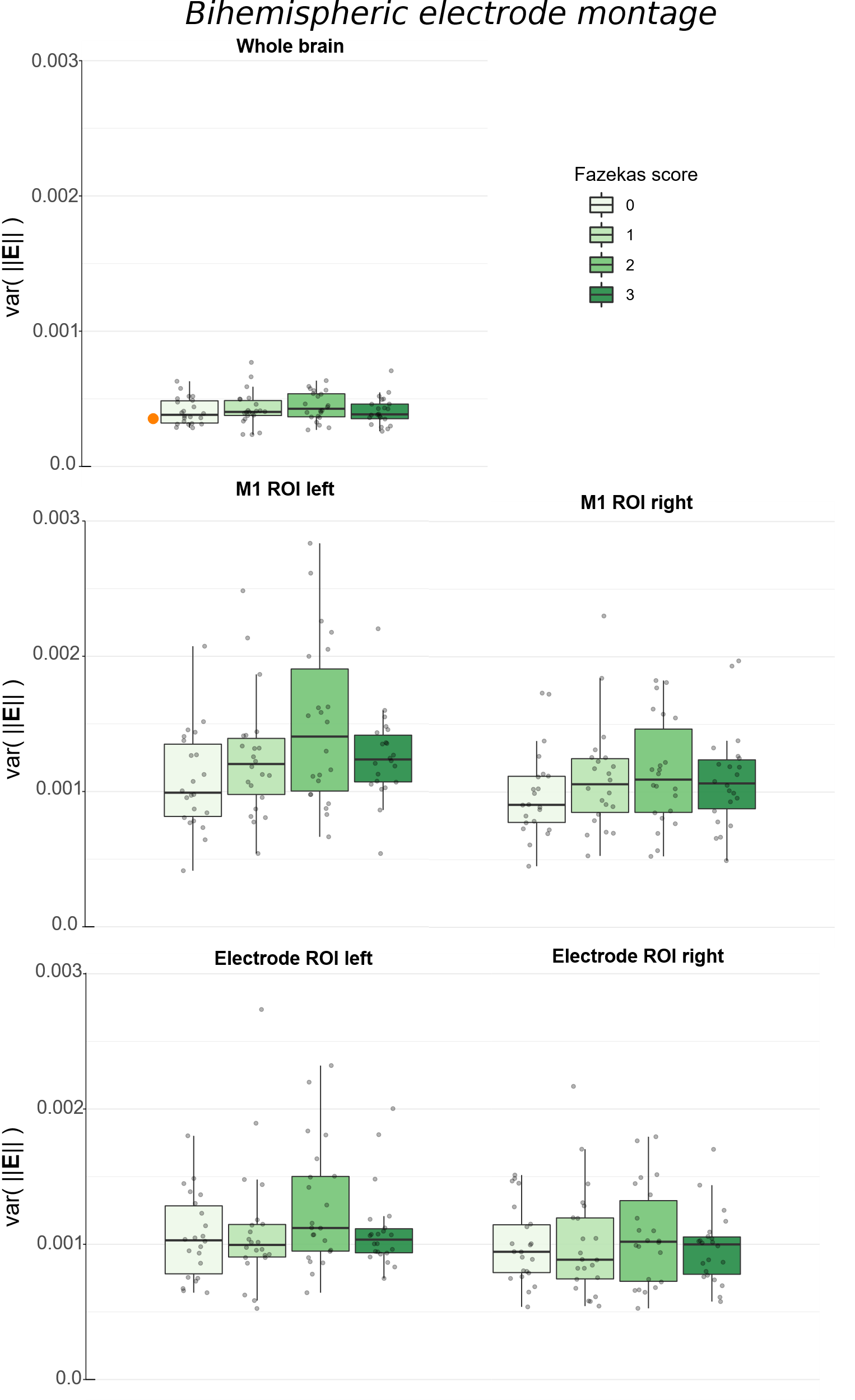


**Supplementary Figure S8** Group-wise boxplots of the spatially averaged variance of the electric field magnitude with the bihemispheric electrode montage. *Values were averaged within the mid-layer regions of interest and on a whole-brain level for every subject (represented as individual dots). Boxplots provide a group comparison. For comparison, the average total variance on a whole-brain level from the uncertainty analysis of our earlier study of a young adult (Saturnino et al., NeuroImage, 2019) was marked with an orange star within the scatter-plot data of the Fazekas 0 group.*


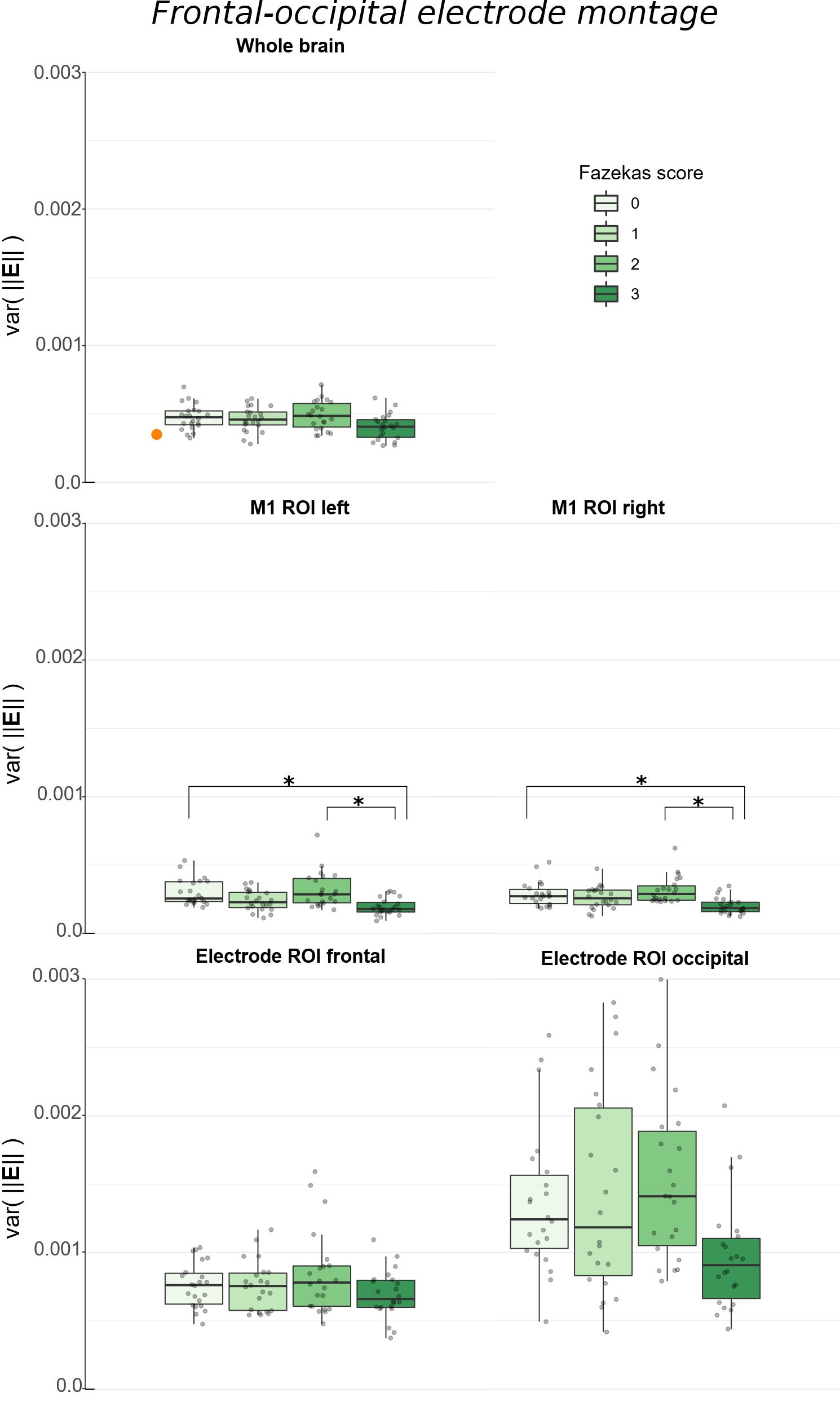


**Supplementary Figure S9** Group-wise boxplots of the spatially averaged variance of the electric field magnitude with the frontal (FPZ)-occipital (OZ) electrode montage. *Values were averaged within the mid-layer regions of interest and on a whole-brain level for every subject (represented as individual dots). Boxplots provide a group comparison. For comparison, the average total variance on a whole-brain level from the uncertainty analysis of our earlier study of a young adult (Saturnino et al., NeuroImage, 2019) was marked with an orange star within the scatter-plot data of the Fazekas 0 group.*


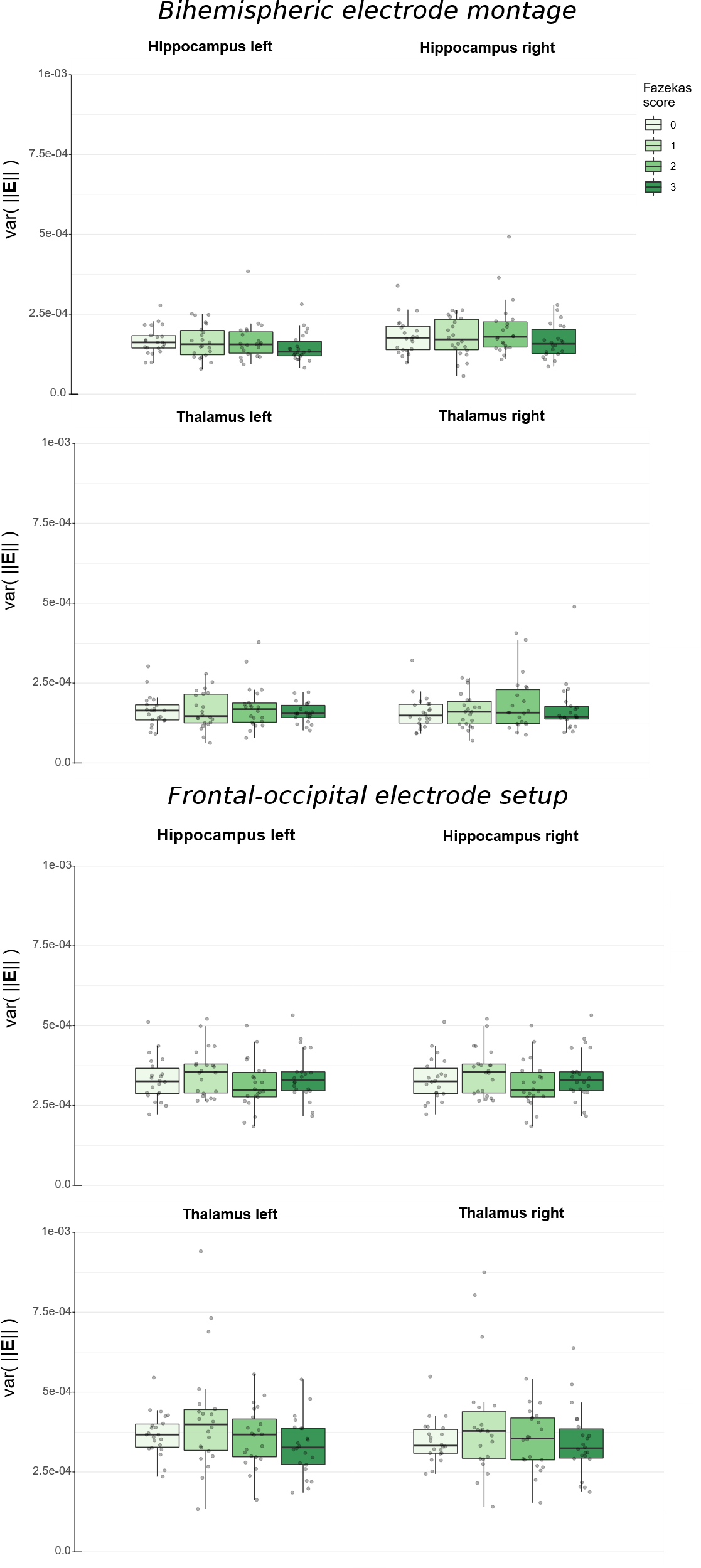


**Supplementary Figure S10** Group-wise boxplots of the spatially averaged variance of the electric field magnitude within the deep ROIs with both electrode montages. *Values were averaged within the deep regions of interest for every subject (represented as individual dots). Boxplots provide a group comparison.*

### S5.2.3 Sobol indices

The Sobol index of the white matter lesions was found to be parametrically increasing with increasing leasion load. However, the it was clearly below the Sobol indices of all other modeled structures (skin, skull, cerebrospinal fluid, gray matter, white matter). Only in the electrode ROIs in the Fazekas 3 group when using the bi-hemispheric electrode setup, the WML Sobol index was found to be in the same order of magnitude as the Sobol indices of CSF and healthy white matter tissue. In the frontal-occipital setup, the influence of white matter lesions on the electric field variance at the mid-layer was diminished and even lower than the influence of healthy white matter and CSF compared to the bihemispheric setup.

Opposing to the mid-layer ROIs, the group averaged Sobol index of healthy white matter was consistently the highest in all deep ROIs. Yet, the white matter lesions remained the lowest contributor to the electric field variance under both electrode configurations.

Significant differences in the group means of the Sobol index of WML (Supplementary Tables S3.1 & 3.2) were identified in all ROIs of the mid-layer and the deep ROIs except the right thalamus when using the bihemispheric electrode setup (Kruskal-Wallis rank test with $\eta^{2}$ as the effect size measure: electrode ROI left: $p\ll.001, \eta^{2}=.617$, electrode ROI right: $p\ll.001, \eta^{2}=.614$, M1 left:$p\ll.001, \eta^{2}=.553$, M1 right: $p\ll.001, \eta^{2}=.486$, whole-brain: $p\ll.001, \eta^{2}=.669$, HPC left: $p\ll.001, \eta^{2}=.180,$ HPC right: $p\ll.001, \eta^{2}=.263,$ TH left: $p=.0017, \eta^{2}=.170$). A likewise significant difference in the WML Sobol index was found for the frontal-occipital setup in all mid-layer ROIs except the frontal ROI with generally smaller effect sizes and for the deep ROIs except the right thalamus with similar effect sizes (Kruskal-Wallis rank test: electrode ROI occipital: $p\ll.001, \eta^{2}=.273$, M1 left:$p\ll.001, \eta^{2}=.423$, M1 right: $p\ll.001, \eta^{2}=.42$, whole-brain: $p\ll.001, \eta^{2}=.457,$ HPC left: $p=.001, \eta^{2}=.186$, HPC right: $p\ll.001, \eta^{2}=.264$, TH left: $p=.0013, \eta^{2}=.179$). This effect was mainly driven by a significantly greater influence of white matter lesions in the high lesion load group (Fazekas 3) than in both the low (Fazekas 1, Dunn’s test: $p\ll.001$ in all ROIs and both electrode setups) and medium lesion load group (Fazekas 2, Dunn’s test: $p\ll.001$ in all ROIs except the left M1 with $p=.0003$ of the bihemispheric setup; for the frontal-occipital setup on the whole-brain level $p\ll.001$, for the M1 ROIs $p=.003$ and the occipital electrode $p=.0011$), but not between the Fazekas 1 and 2 groups across all ROIs in both electrode montages (Supplementary Tables 4.1 & 4.2). In the deep ROIs, the differences in the WML Sobol indices in the bihemispheric electrode montage were primarily driven by a significantly higher contribution of the white matter lesions to the total electric field variance in the Fazekas 3 group as compared to the Fazekas 1 group (HPC left: $p=.0005,$ HPC right: $p\ll.001$, TH left: $p=.0006$) but not between Fazekas 3 and 2 or Fazekas 1 and 2 groups (Supplementary Table 4.3). In the frontal-occipital stimulation condition, the difference in the WML Sobol index was significant between the Fazekas 3 and both other groups in both hippocampi (HPC left: Fazekas 1 $p=.0007,$Fazekas 2 $p=.0084$, HPC right: Fazekas 1 $p=.0001$, Fazekas 2 $p=.0018$) but again only between the Fazekas 3 and Fazekas 1 group in the left thalamus ($p=.0007)$. Also, there was a significant difference in the group average of the Sobol index of healthy white matter in all mid-layer ROIs except the electrode ROI in the bihemispheric setup (Kruskal-Wallis rank test with $\eta^{2}$ as the effect size measure: M1 left:$p\ll.001, \eta^{2}=.291$, M1 right: $p=.0003 , \eta^{2}=.188$, whole-brain: $p=.0002, \eta^{2}=.195$) and in all mid-layer ROIs of the frontal-occipital setup with larger effect sizes (Kruskal-Wallis rank test with $\eta^{2}$ as the effect size measure: electrode ROI frontal: $p=.0014, \eta^{2}=.149$, electrode ROI occipital: $p\ll.001, \eta^{2}=.428$, M1 left:$p\ll.001, \eta^{2}=.462$, M1 right: $p\ll.001, \eta^{2}=.439$, whole-brain: $p\ll.001, \eta^{2}=.482$). This contribution of healthy white matter to the total field variance was significantly decreased in the Fazekas 3 group as compared to all other groups but not between the other groups in the whole brain of the bihemispheric setup (Fazekas 0: $p=.0002$, Fazekas 1: $p=.0049$ Fazekas 2 $p=.0016$) and in all ROIs except the frontal electrode ROI of the frontal-occipital electrode montage (whole brain: Fazekas 0$p\ll.001$, Fazekas 1$p=.0001$, Fazekas 2$p\ll.001$, left M1 ROI: Fazekas 0$p\ll.001$, Fazekas 1$p=.0056$, Fazekas 2$p=.0003$, right M1 ROI: Fazekas 0$p\ll.001$, Fazekas 1$p=.0023$, Fazekas 2$p\ll.001$, occipital electrode ROI: Fazekas 0$p\ll.001$, Fazekas 1$p\ll.001$, Fazekas 2$p\ll.001$). The M1 ROIs of the bihemispheric setups show a similar decrease in the contribution of healthy white matter between the groups Fazekas 3 and 0 (left M1: $p\ll.001$, right M1: $p=.0001$), and 3 and 2 (left M1:$p=.0004$, right M1$p=.0033$), similarly for the frontal electrode ROI of the frontal-occipital setup (Fazekas 3 vs. 2: $p=.0037$, Fazekas 3 vs. 0: $p=.0011,$ but Fazekas 3 vs. 1: $p=.0514$). There was no such difference in the white matter Sobol index in the deep ROIs.


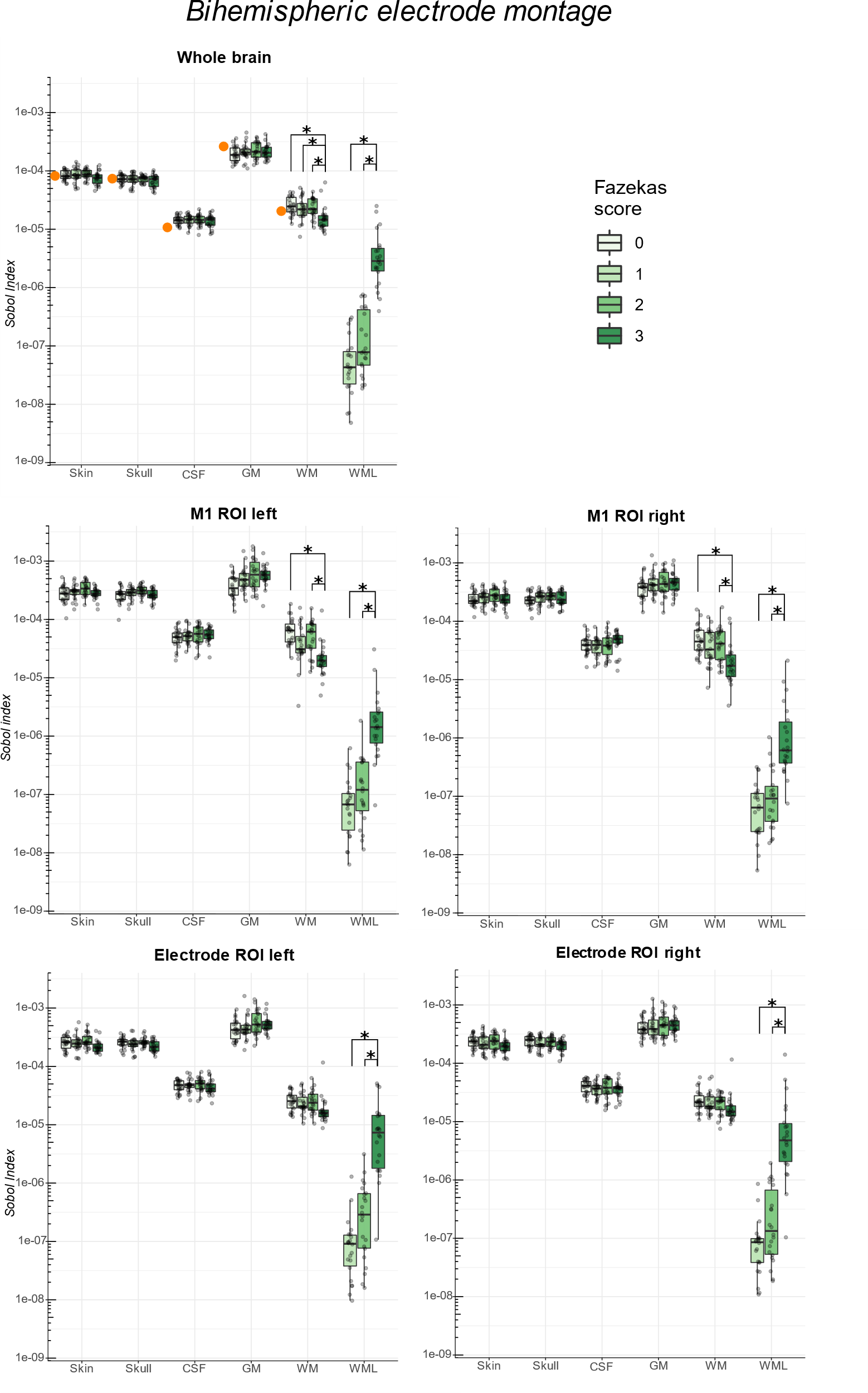


**Supplementary Figure S11** Group-wise boxplots of the Sobol indices of all tissue classes that were modeled uncertain with the bihemispheric electrode montage. *Note that the results are shown in log-scale. Values were averaged within the regions of interest and on a whole-brain level for every subject (represented as individual dots). The boxplots provide a group comparison. For comparison, the average Sobol index on a whole-brain level from the uncertainty analysis of our earlier study of a young adult (Saturnino et al., Neuroimage, 2019) was marked with an orange dot within the scatter plot data of the Fazekas 0 group.*


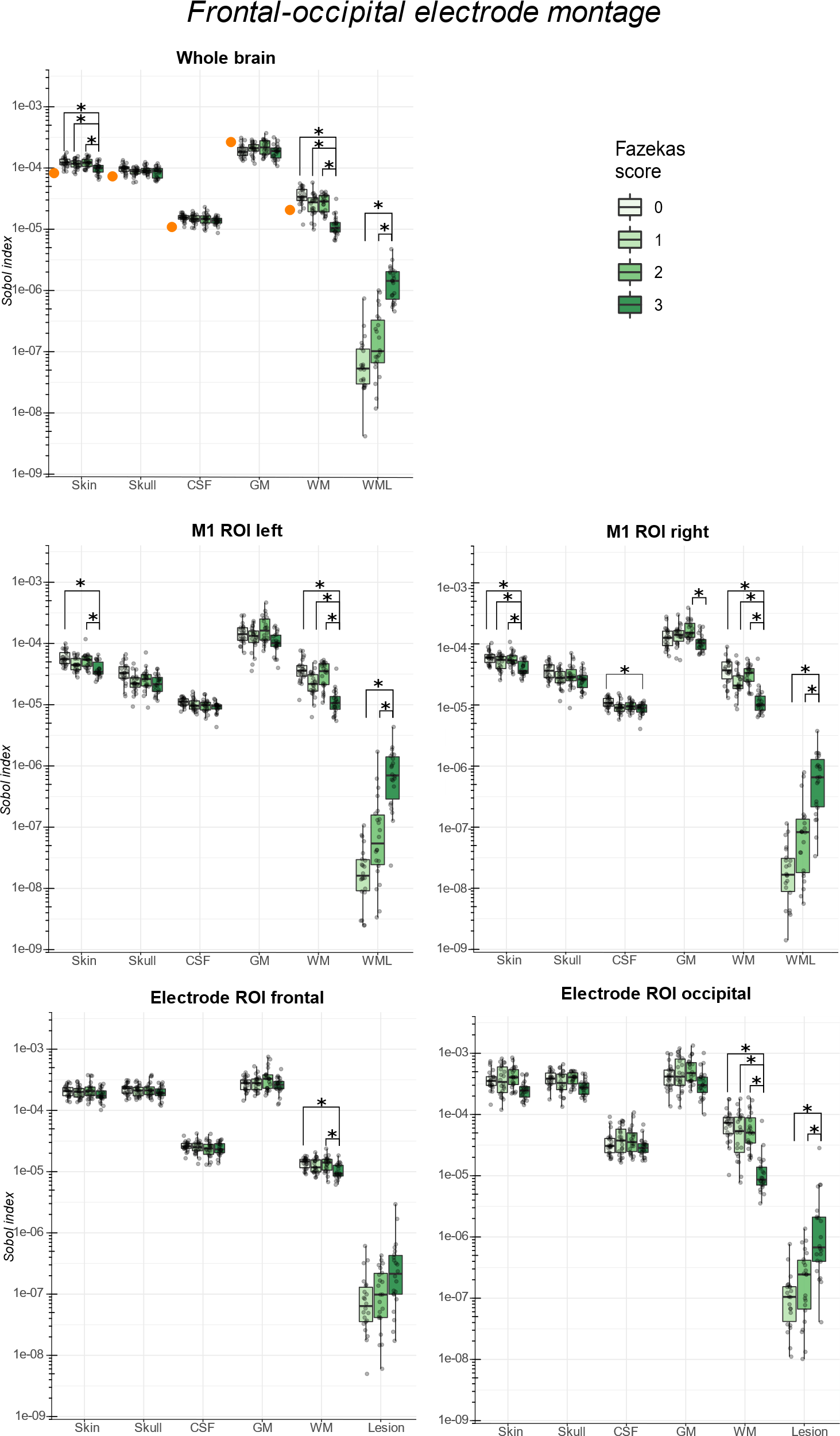


**Supplementary Figure S12** Group-wise boxplots of the Sobol indices of all tissue classes that were modeled uncertain with the frontal (FPZ)-occipital (OZ) electrode montage. *Note that the results are shown in log-scale. Values were averaged within the regions of interest and on a whole-brain level for every subject (represented as individual dots). The boxplots provide a group comparison. For comparison, the average Sobol index on a whole-brain level from the uncertainty analysis of our earlier study of a young adult (Saturnino et al., Neuroimage, 2019) was marked with an orange dot within the scatter plot data of the Fazekas 0 group.*


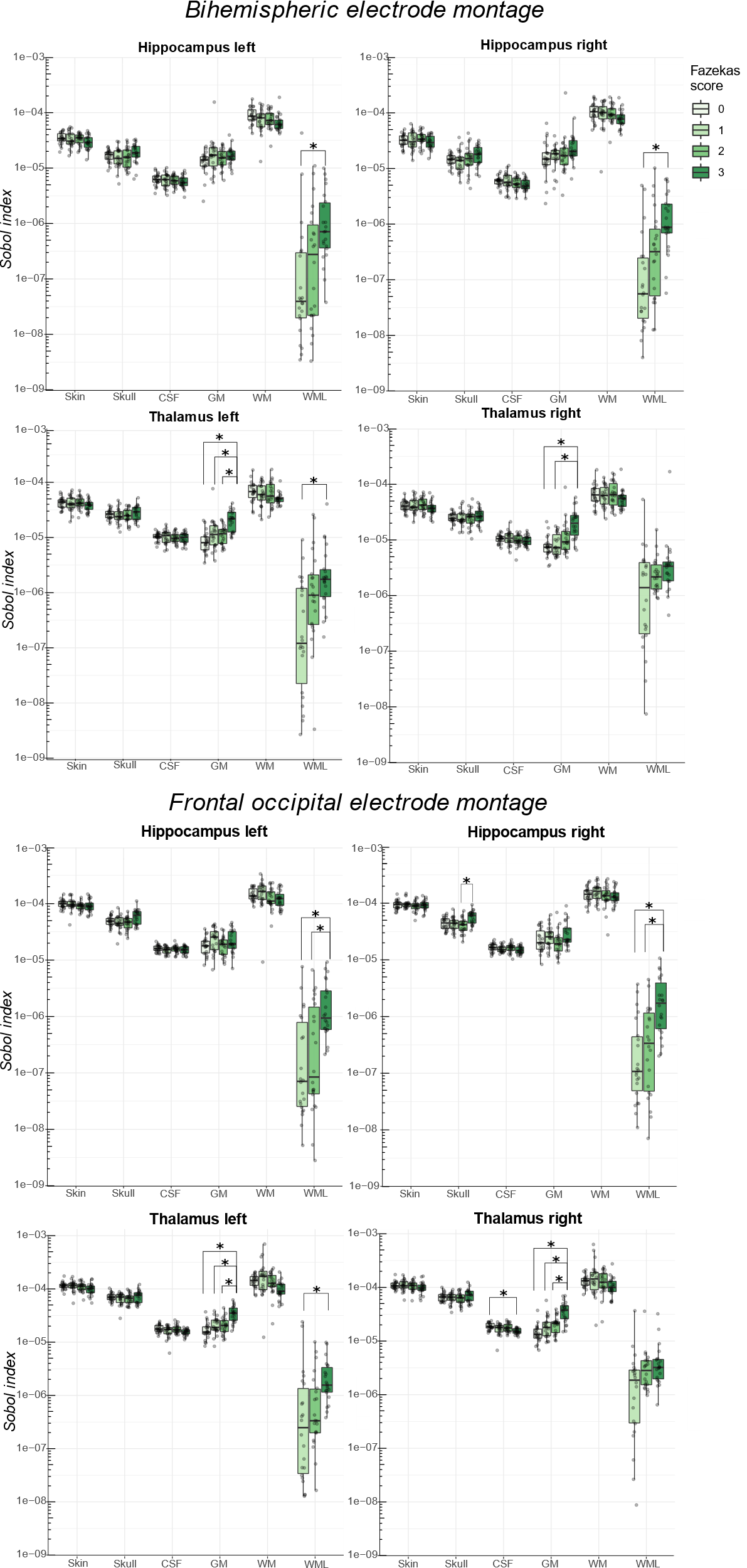


**Supplementary Figure S13** Group-wise boxplots of the Sobol indices of all tissue classes that were modeled uncertain within the subcortical ROIs with both electrode montages. *Note that the results are shown in log-scale. Values were averaged within the regions of interest. Values were averaged within the regions of interest for every subject (represented as individual dots). Boxplots provide a group comparison.*

# S6 Development of the analyzed result quantities across the head model volume, single-subject

Supplementary Figures S14 & S15 illustrate the result quantities of our analysis, that is, the mean electric field magnitude, its standard deviation and the Sobol indices in the intracranial compartments of the head model on a sampling line between the electrodes passing the lesioned tissue. The visualized result quantities were obtained from a single subject from each group.

An apparent, immediate reduction of the mean electric field magnitude in the area of white matter lesions can be observed in subjects from all groups and under all electrode setups. Despite this lowered mean electric field magnitude, its standard deviation remains comparably high. However, the magnitude of both quantities relaxes immediately after and before the lesioned area.

The Sobol indices for skin and skull both contribute a larger share to the total field variance across the whole sampling line, even in non-skin and non-skull regions. The WMLs contributed the most in their respective head model compartments and the immediate surroundings but not in other areas on the sampling line. Its contribution to the total variance rapidly declined outside non-WML regions for both electrode montages. Distance measuring on the sampling line revealed that only 4.3 mm from the boundary of the WML tissue area the WML Sobol index is elevated above ${10}^{-4}$ in the Fazekas 3 subject in both electrode setups.


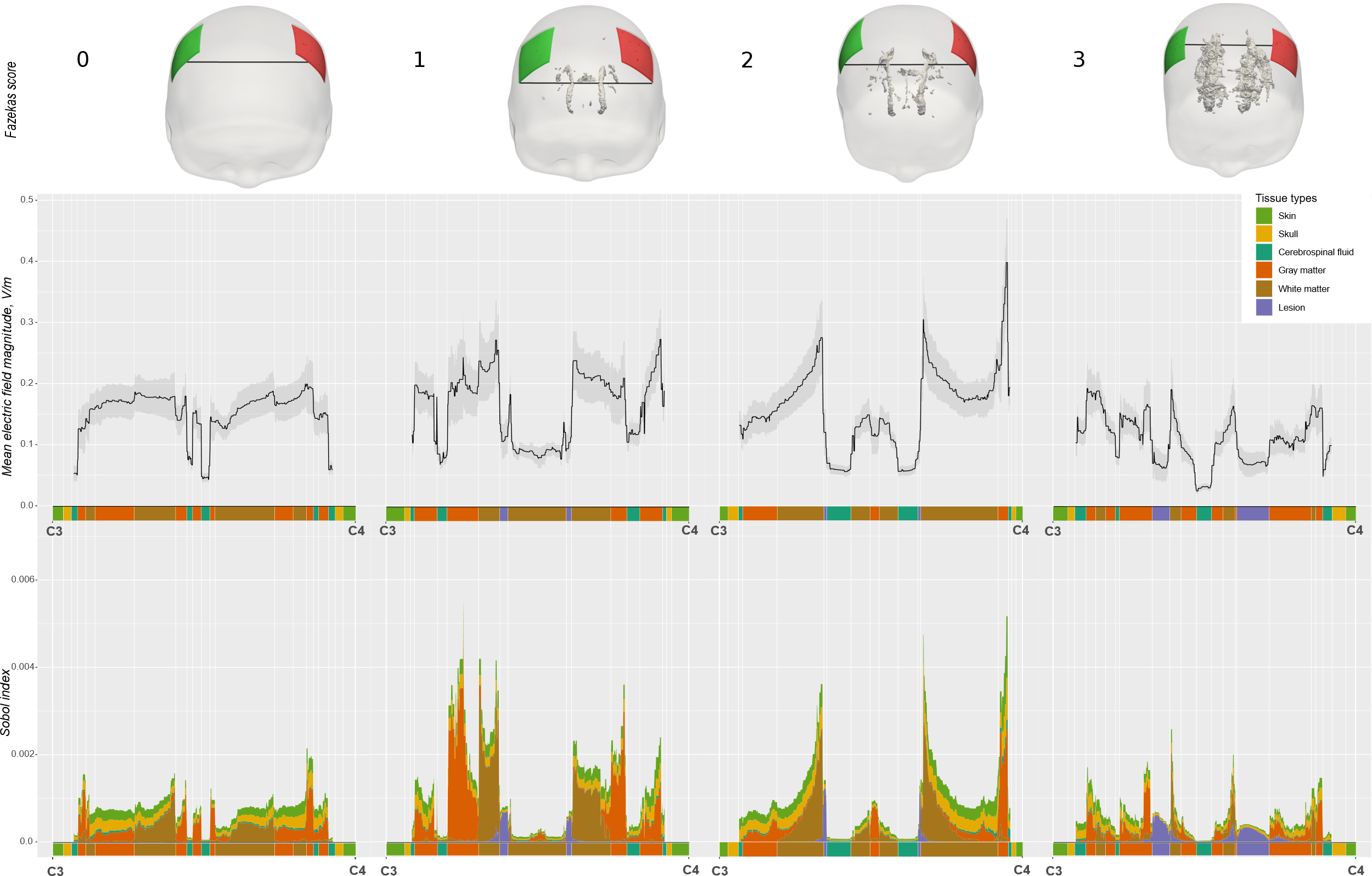


**Supplementary Figure S14** Measures of interest across a sampling line from electrode to electrode through the intracranial volume with the bihemispheric electrode montage. *A distinct sampling line crossing the intracranial volume and specifically the lesion volume was generated between both electrodes for one exemplary subject per group. Top) visualization of the subject, their lesion load and the sampling line. Center) Mean* *electric field magnitude with its standard deviation (shaded) across the sampling line. The bar graphs at the bottom indicate the tissue type present at the respective location on the sampling line. Transitions between tissues are visualized as vertical lines in the background of each chart. Bottom) Stacked area charts of the magnitudes of the Sobol indices. The color encodes the tissue type the respective area represents.*


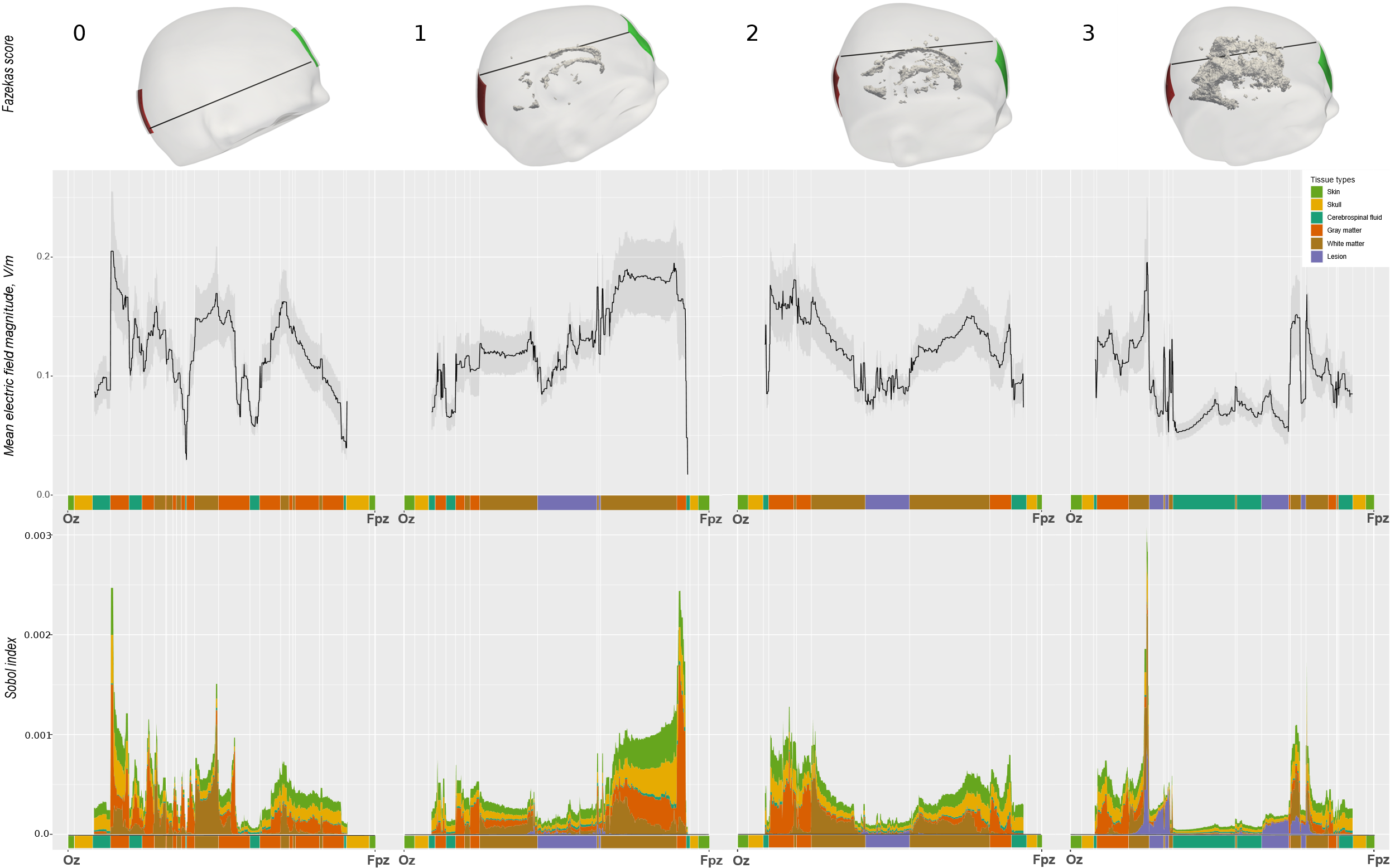


**Supplementary Figure S15** Measures of interest across a sampling line from electrode to electrode through the intracranial volume with the frontal (FPZ)-occipital (OZ) electrode montage. *A distinct sampling line crossing the intracranial volume and specifically the lesion volume was generated between both electrodes for one exemplary subject per group. Top) visualization of the subject, their lesion load and the sampling line. Center)* *Mean electric field magnitude with its standard deviation (shaded) across the sampling line. The bar graphs at the bottom indicate the tissue type present at the respective location on the sampling line. Transitions between tissues are visualized as vertical lines in the background of each chart. Bottom) Stacked area charts of the magnitudes of the Sobol indices. The color encodes the tissue type the respective area represents.*

# S7 Supplementary Tables

| Bihemispheric electrode setup | | | | | | | | |
| --- | --- | --- | --- | --- | --- | --- | --- | --- |
|  | mean( $\left\Vert\boldsymbol{E} \right\Vert$)(V/m) | | | | $Var(\vert\vert\boldsymbol{E\vert}\vert)$(V^2^/m^2^) | | | |
|  | ***F0*** | ***F1*** | ***F2*** | ***F3*** | ***F0*** | ***F1*** | ***F2*** | ***F3*** |
| Whole-brain | $.108$  $sd: .014$ | $.108$  $sd:.017$ | $.112$  $sd:.016$ | $.101$  $sd:.012$ | $.00041$  $sd: .0001$ | $.00043$  $sd:.0001$ | $.00044$  $sd:.0001$ | $.00041$ $sd:.0001$ |
| Left M1 ROI | $.193$  $sd:.038$ | $.201$  $sd:.036$ | $.215$  $sd:.042$ | $.198$  $sd:.03$ | $.0011$  $sd:.0004$ | $.0013$  $sd:.0004$ | $.0015$  $sd:.0006$ | $.0013$  $sd:.0003$ |
| Right M1 ROI | $.175$  $sd:.019$ | $.177$  $sd:.031$ | $.186$  $sd:.027$ | $.177$  $sd:.019$ | $.001$  $sd:.0003$ | $.0011$  $sd:.0004$ | $.0011$  $sd:.0004$ | $.0011$  $sd:.0004$ |
| Elecr. ROI left | $.172$  $sd:.028$ | $.176$  $sd:.036$ | $.187$  $sd:.038$ | $.166$  $sd:.028$ | $.001$  $sd:.0003$ | $.0011$  $sd:.0005$ | $.0013$  $sd:.0005$ | $.0011$  $sd:.0003$ |
| Electr. ROI right | $.164$  $sd:.027$ | $.159$  $sd:.035$ | $.165$  $sd:.037$ | $.15$  $sd:.021$ | $.001$  $sd:.0003$ | $.001$  $sd:.0004$ | $.0011$  $sd:.0004$ | $.001$  $sd:.0003$ |

| Frontal-occipital electrode setup | | | | | | | | |
| --- | --- | --- | --- | --- | --- | --- | --- | --- |
|  | $mean( \left\Vert\boldsymbol{E} \right\Vert)$(V/m) | | | | $Var(\vert\vert\boldsymbol{E\vert}\vert)$(V^2^/m^2^) | | | |
|  | ***F0*** | ***F1*** | ***F2*** | ***F3*** | ***F0*** | ***F1*** | ***F2*** | ***F3*** |
| Whole-brain | $.127$  $sd: .013$ | $.124$  $sd:.013$ | $.127$  $sd:.015$ | $.111$  $sd:.013$ | $.00047$  $sd: .0001$ | $.00046$  $sd:.0001$ | $.00049$  $sd:.0001$ | $.00041$ $sd:.0001$ |
| Left M1 ROI | $.115$  $sd:.013$ | $.104$  $sd:.014$ | $.113$  $sd:.017$ | $.095$  $sd:.013$ | $.0003$  $sd:.0001$ | $.00024$  $sd:.0001$ | $.00032$  $sd:.0001$ | $.00019$  $sd:.0001$ |
| Right M1 ROI | $.112$  $sd:.014$ | $.104$  $sd:.014$ | $.111$  $sd:.013$ | $.095$  $sd:.013$ | $.0003$  $sd:.0001$ | $.0003$  $sd:.0001$ | $.0003$  $sd:.0001$ | $.0002$  $sd:.0001$ |
| Elecr. ROI FPZ | $.129$  $sd:.014$ | $.127$  $sd:.015$ | $.131$  $sd:.023$ | $.122$  $sd:.015$ | $.0008$  $sd:.0002$ | $.0008$  $sd:.0002$ | $.0008$  $sd:.0003$ | $.0007$  $sd:.0002$ |
| Electr. ROI OZ | $.178$  $sd:.045$ | $.183$  $sd:.065$ | $.19$  $sd:.052$ | $.144$  $sd:.031$ | $.0014$  $sd:.0005$ | $.0014$  $sd:.0008$ | $.0015$  $sd:.0006$ | $.001$  $sd:.0004$ |

**Supplementary Table S1.1** Group means and standard deviations of the mean electric field magnitude ($\bar{||\boldsymbol{E|}|}$) and its variance ($Var(||\boldsymbol{E|}|)$) spatially averaged in the mid-layer ROIs with *both* electrode configurations. Values are listed for the five regions of interest at the cortical mid-layer of each group. Abbreviations: *F0/1/2/3 = Fazekas 0/1/2/3 group*, *sd = standard deviation, Electr. = electrode*

| Bihemispheric electrode setup | | | | | | | | |
| --- | --- | --- | --- | --- | --- | --- | --- | --- |
|  | mean( $\left\Vert\boldsymbol{E} \right\Vert$)(V/m) | | | | $Var(\vert\vert\boldsymbol{E\vert}\vert)$(V^2^/m^2^) | | | |
|  | ***F0*** | ***F1*** | ***F2*** | ***F3*** | ***F0*** | ***F1*** | ***F2*** | ***F3*** |
| Hippocamp. left | $.093$  $sd:.012$ | $.09$  $sd:.015$ | $.089$  $sd:.011$ | $.082$  $sd:.012$ | $.00017$  $sd:.00004$ | $.00016$  $sd:.00005$ | $.00017$  $sd:.00006$ | $.00015$  $sd:.00005$ |
| Hippocamp.  right | $.091$  $sd:.015$ | $.091$  $sd:.016$ | $.094$  $sd:.011$ | $.084$  $sd:.012$ | $.00018$  $sd:.00006$ | $.00018$  $sd:.00006$ | $.0002$  $sd:.00009$ | $.00017$  $sd:.00005$ |
| Thalamus  left | $.095$  $sd:.013$ | $.095$  $sd:.015$ | $.095$  $sd:.015$ | $.086$  $sd:.011$ | $.00016$  $sd:.00005$ | $.00016$  $sd:.00006$ | $.00017$  $sd:.00007$ | $.00016$  $sd:.00003$ |
| Thalamus  right | $.094$  $sd:.014$ | $.094$  $sd:.014$ | $.097$  $sd:.014$ | $.086$  $sd:.014$ | $.00016$  $sd:.00005$ | $.00016$  $sd:.00005$ | $.00018$  $sd:.00009$ | $.00017$  $sd:.00008$ |

| Frontal-occipital electrode setup | | | | | | | | |
| --- | --- | --- | --- | --- | --- | --- | --- | --- |
|  | mean( $\left\Vert\boldsymbol{E} \right\Vert$)(V/m) | | | | $Var(\vert\vert\boldsymbol{E\vert}\vert)$(V^2^/m^2^) | | | |
|  | ***F0*** | ***F1*** | ***F2*** | ***F3*** | ***F0*** | ***F1*** | ***F2*** | ***F3*** |
| Hippocamp. left | $.134$  $sd:.014$ | $.136$  $sd:.019$ | $.129$  $sd:.016$ | $.123$  $sd:.016$ | $.00034$  $sd:.00006$ | $.00036$  $sd:.0001$ | $.00031$  $sd:.00007$ | $.00032$  $sd:.00009$ |
| Hippocamp.  right | $.134$  $sd:.015$ | $.138$  $sd:.015$ | $.132$  $sd:.017$ | $.126$  $sd:.013$ | $.00033$  $sd:.00007$ | $.00036$  $sd:.00007$ | $.00031$  $sd:.00008$ | $.00034$  $sd:.00008$ |
| Thalamus  left | $.137$  $sd:.013$ | $.138$  $sd:.018$ | $.136$  $sd:.017$ | $.124$  $sd:.016$ | $.00037$  $sd:.00007$ | $.00042$  $sd:.00018$ | $.00036$  $sd:.00009$ | $.00033$  $sd:.00009$ |
| Thalamus  right | $.136$  $sd:.013$ | $.137$  $sd:.018$ | $.135$  $sd:.017$ | $.123$  $sd:.016$ | $.00035$  $sd:.00007$ | $.0004$  $sd:.00018$ | $.00035$  $sd:.00009$ | $.00034$  $sd:.00011$ |

**Supplementary Table S1.2** Group means and standard deviations of the mean electric field magnitude ($\bar{||\boldsymbol{E|}|}$) and its variance ($Var(||\boldsymbol{E|}|)$) spatially averaged in the mid-layer ROIs with *both* electrode configurations. Values are listed for the four subcortical regions of interest of each group. Abbreviations: *F0/1/2/3 = Fazekas 0/1/2/3 group*, *sd = standard deviation, Hippocamp. = Hippocampus*

| V^2^/m^2^ | *Sobol index skin* | | | | *Sobol index skull* | | | | |
| --- | --- | --- | --- | --- | --- | --- | --- | --- | --- |
|  | ***Fazekas 0*** | ***Fazekas 1*** | ***Fazekas 2*** | ***Fazekas 3*** | ***Fazekas 0*** | ***Fazekas 1*** | ***Fazekas 2*** | ***Fazekas 3*** | |
| Whole-brain | $8.54\cdot{10}^{-5}$  $sd:1.64\cdot{10}^{-5}$ | $8.75\cdot{10}^{-5}$  $sd:2.45\cdot{10}^{-5}$ | $8.92\cdot{10}^{-5}$  $sd:1.63\cdot{10}^{-5}$ | $7.65\cdot{10}^{-5}$  $sd:1.92\cdot{10}^{-5}$ | $7.42\cdot{10}^{-5}$  $sd:1.24\cdot{10}^{-5}$ | $7.35\cdot{10}^{-5}$  $sd:1.66\cdot{10}^{-5}$ | $7.37\cdot{10}^{-5}$  $sd:9.77\cdot{10}^{-6}$ | $6.89\cdot{10}^{-5}$  $sd:1.65\cdot{10}^{-5}$ | |
| M1 ROI left | $2.85\cdot{10}^{-4}$  $sd:9.18\cdot{10}^{-5}$ | $3.05\cdot{10}^{-4}$  $sd:8.74\cdot{10}^{-5}$ | $3.49\cdot{10}^{-4}$  $sd:9.71\cdot{10}^{-5}$ | $2.81\cdot{10}^{-4}$  $sd:5.95\cdot{10}^{-5}$ | $2.72\cdot{10}^{-4}$  $sd:7.71\cdot{10}^{-5}$ | $2.85\cdot{10}^{-4}$  $sd:6.42\cdot{10}^{-5}$ | $3.19\cdot{10}^{-4}$  $sd:6.2\cdot{10}^{-5}$ | $2.68\cdot{10}^{-4}$  $sd:5.86\cdot{10}^{-5}$ | |
| M1 ROI right | $2.46\cdot{10}^{-4}$  $sd:7.25\cdot{10}^{-5}$ | $2.61\cdot{10}^{-4}$  $sd:7.43\cdot{10}^{-5}$ | $2.79\cdot{10}^{-4}$  $sd:7.95\cdot{10}^{-5}$ | $2.52\cdot{10}^{-4}$  $sd:8.06\cdot{10}^{-5}$ | $2.42\cdot{10}^{-4}$  $sd:6.72\cdot{10}^{-5}$ | $2.6\cdot{10}^{-4}$  $sd:6.19\cdot{10}^{-5}$ | $2.7\cdot{10}^{-4}$  $sd:5.65\cdot{10}^{-5}$ | $2.56\cdot{10}^{-4}$  $sd:7.44\cdot{10}^{-5}$ | |
| Electr. ROI left | $2.59\cdot{10}^{-4}$  $sd:6.62\cdot{10}^{-5}$ | $2.6\cdot{10}^{-4}$  $sd:9.65\cdot{10}^{-5}$ | $2.89\cdot{10}^{-4}$  $sd:8.44\cdot{10}^{-5}$ | $2.26\cdot{10}^{-4}$  $sd:6.32\cdot{10}^{-5}$ | $2.59\cdot{10}^{-4}$  $sd:5.54\cdot{10}^{-5}$ | $2.47\cdot{10}^{-4}$  $sd:6.86\cdot{10}^{-5}$ | $2.66\cdot{10}^{-4}$  $sd:5.02\cdot{10}^{-5}$ | $2.26\cdot{10}^{-4}$  $sd:4.94\cdot{10}^{-5}$ | |
| Electr. ROI right | $2.43\cdot{10}^{-4}$  $sd:6.21\cdot{10}^{-5}$ | $4.09\cdot{10}^{-4}$  $sd:2.14\cdot{10}^{-4}$ | $2.43\cdot{10}^{-4}$  $sd:7.36\cdot{10}^{-5}$ | $1.97\cdot{10}^{-4}$  $sd:5.35\cdot{10}^{-5}$ | $2.46\cdot{10}^{-4}$  $sd:5.2\cdot{10}^{-5}$ | $3.63\cdot{10}^{-4}$  $sd:1.48\cdot{10}^{-4}$ | $2.36\cdot{10}^{-4}$  $sd:4.86\cdot{10}^{-5}$ | $2.05\cdot{10}^{-4}$  $sd:4.62\cdot{10}^{-5}$ | |
|  | **Sobol index gray matter** | | | | **Sobol index white matter** | | | |  |
| Whole-brain | $2.03\cdot{10}^{-4}$  $sd:6.32\cdot{10}^{-5}$ | $2.21\cdot{10}^{-4}$  $sd:7.87\cdot{10}^{-5}$ | $2.37\cdot{10}^{-4}$  $sd:7.57\cdot{10}^{-5}$ | $2.2\cdot{10}^{-4}$  $sd:6.84\cdot{10}^{-5}$ | $2.64\cdot{10}^{-5}$  $sd:9.13\cdot{10}^{-6}$ | $2.44\cdot{10}^{-5}$  $sd:1.1\cdot{10}^{-5}$ | $2.5\cdot{10}^{-5}$  $sd:8.79\cdot{10}^{-6}$ | $1.76\cdot{10}^{-5}$  $sd:1.28\cdot{10}^{-5}$ | |
| M1 ROI left | $3.92\cdot{10}^{-4}$  $sd:1.74\cdot{10}^{-4}$ | $5.46\cdot{10}^{-4}$  $sd:2.9\cdot{10}^{-4}$ | $6.92\cdot{10}^{-4}$  $sd:4.32\cdot{10}^{-4}$ | $6.05\cdot{10}^{-4}$  $sd:2.4\cdot{10}^{-4}$ | $7.03\cdot{10}^{-5}$  $sd:3.85\cdot{10}^{-5}$ | $4.66\cdot{10}^{-5}$  $sd:3.61\cdot{10}^{-5}$ | $5.88\cdot{10}^{-5}$  $sd:3.55\cdot{10}^{-5}$ | $2.61\cdot{10}^{-5}$  $sd:2.77\cdot{10}^{-5}$ | |
| M1 ROI right | $3.89\cdot{10}^{-4}$  $sd:1.65\cdot{10}^{-4}$ | $4.83\cdot{10}^{-4}$  $sd:2.58\cdot{10}^{-4}$ | $4.91\cdot{10}^{-4}$  $sd:2.38\cdot{10}^{-4}$ | $4.92\cdot{10}^{-4}$  $sd:2.15\cdot{10}^{-4}$ | $5.56\cdot{10}^{-5}$  $sd:3.54\cdot{10}^{-5}$ | $4.38\cdot{10}^{-5}$  $sd:2.9\cdot{10}^{-5}$ | $4.94\cdot{10}^{-5}$  $sd:3.56\cdot{10}^{-5}$ | $2.53\cdot{10}^{-5}$  $sd:2.64\cdot{10}^{-5}$ | |
| Electr. ROI left | $4.54\cdot{10}^{-4}$  $sd:1.84\cdot{10}^{-4}$ | $5.12\cdot{10}^{-4}$  $sd:2.99\cdot{10}^{-4}$ | $6.26\cdot{10}^{-4}$  $sd:3.12\cdot{10}^{-4}$ | $5.52\cdot{10}^{-4}$  $sd:2.01\cdot{10}^{-4}$ | $2.69\cdot{10}^{-5}$  $sd:9.73\cdot{10}^{-6}$ | $2.49\cdot{10}^{-5}$  $sd:1.04\cdot{10}^{-5}$ | $2.68\cdot{10}^{-5}$  $sd:1.28\cdot{10}^{-5}$ | $2.14\cdot{10}^{-5}$  $sd:2.19\cdot{10}^{-5}$ | |
| Electr. ROI right | $4.29\cdot{10}^{-4}$  $sd:1.64\cdot{10}^{-4}$ | $5.4\cdot{10}^{-4}$  $sd:3.26\cdot{10}^{-4}$ | $5.09\cdot{10}^{-4}$  $sd:2.41\cdot{10}^{-4}$ | $4.77\cdot{10}^{-4}$  $sd:1.72\cdot{10}^{-4}$ | $2.43\cdot{10}^{-5}$  $sd:1.14\cdot{10}^{-5}$ | $6.31\cdot{10}^{-5}$  $sd:4.68\cdot{10}^{-5}$ | $2.17\cdot{10}^{-5}$  $sd:7.77\cdot{10}^{-6}$ | $2.03\cdot{10}^{-5}$  $sd:2.2\cdot{10}^{-5}$ | |
|  | **Sobol index cerebrospinal fluid** | | | | **Sobol index white matter lesions** | | | | |
| Whole-brain | $1.46\cdot{10}^{-5}$  $sd:2.58\cdot{10}^{-6}$ | $1.47\cdot{10}^{-5}$  $sd:2.81\cdot{10}^{-6}$ | $1.46\cdot{10}^{-5}$  $sd:3.14\cdot{10}^{-6}$ | $1.4\cdot{10}^{-5}$  $sd:3.3\cdot{10}^{-6}$ | / | $7.84\cdot{10}^{-8}$  $sd:8.89\cdot{10}^{-8}$ | $2.24\cdot{10}^{-7}$  $sd:2.54\cdot{10}^{-7}$ | $5.17\cdot{10}^{-6}$  $sd:6.36\cdot{10}^{-6}$ | |
| M1 ROI left | $5.06\cdot{10}^{-5}$  $sd:1.79\cdot{10}^{-5}$ | $5.46\cdot{10}^{-5}$  $sd:1.74\cdot{10}^{-5}$ | $5.78\cdot{10}^{-5}$  $sd:2.13\cdot{10}^{-5}$ | $5.59\cdot{10}^{-5}$  $sd:1.45\cdot{10}^{-5}$ | / | $1.08\cdot{10}^{-7}$  $sd:1.41\cdot{10}^{-7}$ | $2.43\cdot{10}^{-7}$  $sd:3.84\cdot{10}^{-7}$ | $3.51\cdot{10}^{-6}$  $sd:6.7\cdot{10}^{-6}$ | |
| M1 ROI right | $4.11\cdot{10}^{-5}$  $sd:1.54\cdot{10}^{-5}$ | $3.91\cdot{10}^{-5}$  $sd:1.39\cdot{10}^{-6}$ | $4.2\cdot{10}^{-5}$  $sd:1.98\cdot{10}^{-5}$ | $4.71\cdot{10}^{-5}$  $sd:1.65\cdot{10}^{-6}$ | / | $9.27\cdot{10}^{-8}$  $sd:9.32\cdot{10}^{-8}$ | $1.67\cdot{10}^{-7}$  $sd:2.34\cdot{10}^{-7}$ | $2.5\cdot{10}^{-6}$  $sd:4.77\cdot{10}^{-6}$ | |
| Electr. ROI left | $4.67\cdot{10}^{-5}$  $sd:1.07\cdot{10}^{-5}$ | $4.9\cdot{10}^{-5}$  $sd:1.27\cdot{10}^{-5}$ | $5.0\cdot{10}^{-5}$  $sd:1.28\cdot{10}^{-5}$ | $4.63\cdot{10}^{-5}$  $sd:1.46\cdot{10}^{-5}$ | / | $1.53\cdot{10}^{-7}$  $sd:2.75\cdot{10}^{-7}$ | $5.3\cdot{10}^{-7}$  $sd:7.12\cdot{10}^{-7}$ | $1.21\cdot{10}^{-5}$  $sd:1.52\cdot{10}^{-5}$ | |
| Electr. ROI right | $4.15\cdot{10}^{-5}$  $sd:1.06\cdot{10}^{-5}$ | $4.23\cdot{10}^{-5}$  $sd:2.18\cdot{10}^{-5}$ | $3.93\cdot{10}^{-5}$  $sd:1.46\cdot{10}^{-5}$ | $3.68\cdot{10}^{-5}$  $sd:1.08\cdot{10}^{-5}$ | / | $1.41\cdot{10}^{-7}$  $sd:1.67\cdot{10}^{-7}$ | $4.02\cdot{10}^{-7}$  $sd:5.17\cdot{10}^{-7}$ | $1.51\cdot{10}^{-5}$  $sd:3.08\cdot{10}^{-5}$ | |

**Supplementary Table S2.1** Group means and standard deviations of the Sobol indices in the mid-layer ROIs for the ***bihemispheric*** electrode montage. The means and standard deviations of the Sobol indices of the tissue classes skin, skull, cerebrospinal fluid, gray matter, white matter and white matter lesions in the five regions of interest at the cortical mid-layer for each group are listed here.

| V^2^/m^2^ | Sobol index skin | | | | Sobol index skull | | | | |
| --- | --- | --- | --- | --- | --- | --- | --- | --- | --- |
|  | ***Fazekas 0*** | ***Fazekas 1*** | ***Fazekas 2*** | ***Fazekas 3*** | ***Fazekas 0*** | ***Fazekas 1*** | | ***Fazekas 2*** | ***Fazekas 3*** |
| Whole-brain | $1.25\cdot{10}^{-4}$  $sd:2.23\cdot{10}^{-5}$ | $1.18\cdot{10}^{-4}$  $sd:2.07\cdot{10}^{-5}$ | $1.24\cdot{10}^{-4}$  $sd:2.25\cdot{10}^{-5}$ | $9.98\cdot{10}^{-5}$  $sd:2.1\cdot{10}^{-5}$ | $9.89\cdot{10}^{-5}$  $sd:1.58\cdot{10}^{-5}$ | $8.65\cdot{10}^{-5}$  $sd:1.3\cdot{10}^{-5}$ | | $9.28\cdot{10}^{-5}$  $sd:1.36\cdot{10}^{-5}$ | $8.6\cdot{10}^{-5}$  $sd:1.74\cdot{10}^{-5}$ |
| M1 ROI left | $6.04\cdot{10}^{-5}$  $sd:1.68\cdot{10}^{-5}$ | $4.7\cdot{10}^{-5}$  $sd:1.2\cdot{10}^{-5}$ | $5.43\cdot{10}^{-5}$  $sd:1.75\cdot{10}^{-5}$ | $3.91\cdot{10}^{-5}$  $sd:1.18\cdot{10}^{-5}$ | $3.49\cdot{10}^{-5}$  $sd:1.33\cdot{10}^{-5}$ | $2.4\cdot{10}^{-5}$  $sd:8.67\cdot{10}^{-6}$ | | $2.73\cdot{10}^{-5}$  $sd:1.24\cdot{10}^{-5}$ | $2.3\cdot{10}^{-5}$  $sd:7.95\cdot{10}^{-6}$ |
| M1 ROI right | $6.16\cdot{10}^{-5}$  $sd:1.6\cdot{10}^{-5}$ | $5.15\cdot{10}^{-5}$  $sd:1.36\cdot{10}^{-5}$ | $5.76\cdot{10}^{-5}$  $sd:1.72\cdot{10}^{-5}$ | $4.13\cdot{10}^{-5}$  $sd:1.13\cdot{10}^{-5}$ | $3.78\cdot{10}^{-5}$  $sd:1.16\cdot{10}^{-5}$ | $2.86\cdot{10}^{-5}$  $sd:8.99\cdot{10}^{-6}$ | | $3.27\cdot{10}^{-5}$  $sd:1.47\cdot{10}^{-5}$ | $2.63\cdot{10}^{-5}$  $sd:8.95\cdot{10}^{-6}$ |
| Electr. ROI FPZ | $2.08\cdot{10}^{-4}$  $sd:4.41\cdot{10}^{-5}$ | $2.03\cdot{10}^{-4}$  $sd:4.7\cdot{10}^{-4}$ | $2.2\cdot{10}^{-4}$  $sd:7.24\cdot{10}^{-5}$ | $1.83\cdot{10}^{-4}$  $sd:4.55\cdot{10}^{-5}$ | $2.34\cdot{10}^{-4}$  $sd:1.06\cdot{10}^{-4}$ | $2.14\cdot{10}^{-4}$  $sd:4.5\cdot{10}^{-5}$ | | $2.27\cdot{10}^{-4}$  $sd:6.7\cdot{10}^{-5}$ | $1.99\cdot{10}^{-4}$  $sd:4.65\cdot{10}^{-5}$ |
| Electr. ROI OZ | $3.87\cdot{10}^{-4}$  $sd:1.52\cdot{10}^{-4}$ | $3.81\cdot{10}^{-4}$  $sd:1.98\cdot{10}^{-4}$ | $4.29\cdot{10}^{-4}$  $sd:1.73\cdot{10}^{-4}$ | $2.62\cdot{10}^{-4}$  $sd:9.3\cdot{10}^{-5}$ | $3.93\cdot{10}^{-4}$  $sd:1.08\cdot{10}^{-4}$ | $3.43\cdot{10}^{-4}$  $sd:1.35\cdot{10}^{-4}$ | | $3.92\cdot{10}^{-4}$  $sd:9.2\cdot{10}^{-5}$ | $2.87\cdot{10}^{-4}$  $sd:8.45\cdot{10}^{-5}$ |
|  | **Sobol index gray matter** | | | | **Sobol index white matter** | | | | |
| Whole-brain | $1.96\cdot{10}^{-4}$  $sd:4.89\cdot{10}^{-5}$ | $2.11\cdot{10}^{-4}$  $sd:4.66\cdot{10}^{-5}$ | $2.25\cdot{10}^{-4}$  $sd:6.22\cdot{10}^{-5}$ | $1.88\cdot{10}^{-4}$  $sd:5.23\cdot{10}^{-5}$ | $3.6\cdot{10}^{-5}$  $sd:1.16\cdot{10}^{-5}$ | $2.68\cdot{10}^{-5}$  $sd:1.1\cdot{10}^{-5}$ | | $2.77\cdot{10}^{-5}$  $sd:8.76\cdot{10}^{-6}$ | $1.21\cdot{10}^{-5}$  $sd:5.43\cdot{10}^{-6}$ |
| M1 ROI left | $1.52\cdot{10}^{-4}$  $sd:5.74\cdot{10}^{-5}$ | $1.32\cdot{10}^{-4}$  $sd:5.33\cdot{10}^{-5}$ | $1.88\cdot{10}^{-4}$  $sd:9.86\cdot{10}^{-5}$ | $1.09\cdot{10}^{-4}$  $sd:4.18\cdot{10}^{-5}$ | $3.97\cdot{10}^{-5}$  $sd:1.7\cdot{10}^{-5}$ | $2.35\cdot{10}^{-5}$  $sd:1.04\cdot{10}^{-5}$ | | $3.37\cdot{10}^{-5}$  $sd:1.41\cdot{10}^{-5}$ | $1.22\cdot{10}^{-5}$  $sd:7.08\cdot{10}^{-6}$ |
| M1 ROI right | $1.34\cdot{10}^{-4}$  $sd:5.51\cdot{10}^{-5}$ | $1.45\cdot{10}^{-4}$  $sd:5.48\cdot{10}^{-5}$ | $1.81\cdot{10}^{-4}$  $sd:6.84\cdot{10}^{-5}$ | $1.09\cdot{10}^{-4}$  $sd:3.77\cdot{10}^{-5}$ | $3.97\cdot{10}^{-5}$  $sd:1.8\cdot{10}^{-5}$ | $2.6\cdot{10}^{-5}$  $sd:1.37\cdot{10}^{-5}$ | | $3.27\cdot{10}^{-5}$  $sd:1.15\cdot{10}^{-5}$ | $1.31\cdot{10}^{-5}$  $sd:1.05\cdot{10}^{-5}$ |
| Electr. ROI FPZ | $2.74\cdot{10}^{-4}$  $sd:7.07\cdot{10}^{-5}$ | $2.89\cdot{10}^{-4}$  $sd:8.58\cdot{10}^{-5}$ | $3.45\cdot{10}^{-4}$  $sd:1.56\cdot{10}^{-4}$ | $2.65\cdot{10}^{-4}$  $sd:8.05\cdot{10}^{-5}$ | $1.4\cdot{10}^{-5}$  $sd:2.7\cdot{10}^{-6}$ | $1.29\cdot{10}^{-5}$  $sd:3.56\cdot{10}^{-6}$ | | $1.38\cdot{10}^{-5}$  $sd:3.88\cdot{10}^{-6}$ | $1.05\cdot{10}^{-5}$  $sd:3.0\cdot{10}^{-6}$ |
| Electr. ROI OZ | $4.58\cdot{10}^{-4}$  $sd:2.24\cdot{10}^{-4}$ | $5.04\cdot{10}^{-4}$  $sd:3.1\cdot{10}^{-4}$ | $5.73\cdot{10}^{-4}$  $sd:3.8\cdot{10}^{-4}$ | $3.59\cdot{10}^{-4}$  $sd:2.07\cdot{10}^{-4}$ | $7.74\cdot{10}^{-5}$  $sd:4.23\cdot{10}^{-5}$ | $5.57\cdot{10}^{-5}$  $sd:3.96\cdot{10}^{-5}$ | | $6.74\cdot{10}^{-5}$  $sd:4.98\cdot{10}^{-5}$ | $1.39\cdot{10}^{-5}$  $sd:1.58\cdot{10}^{-5}$ |
|  | **Sobol index cerebrospinal fluid** | | | | **Sobol index white matter lesions** | | | | |
| Whole-brain | $1.55\cdot{10}^{-5}$  $sd:2.04\cdot{10}^{-6}$ | $1.49\cdot{10}^{-5}$  $sd:2.38\cdot{10}^{-6}$ | $1.49\cdot{10}^{-5}$  $sd:3.26\cdot{10}^{-6}$ | $1.37\cdot{10}^{-5}$  $sd:1.95\cdot{10}^{-6}$ | / | | $1.02\cdot{10}^{-7}$  $sd:1.55\cdot{10}^{-7}$ | $2.52\cdot{10}^{-7}$  $sd:2.92\cdot{10}^{-7}$ | $1.56\cdot{10}^{-6}$  $sd:1.03\cdot{10}^{-6}$ |
| M1 ROI left | $1.11\cdot{10}^{-5}$  $sd:1.7\cdot{10}^{-6}$ | $1.02\cdot{10}^{-5}$  $sd:2.35\cdot{10}^{-6}$ | $9.89\cdot{10}^{-6}$  $sd:2.25\cdot{10}^{-6}$ | $9.19\cdot{10}^{-6}$  $sd:1.77\cdot{10}^{-6}$ | / | | $2.69\cdot{10}^{-8}$  $sd:2.73\cdot{10}^{-8}$ | $1.93\cdot{10}^{-7}$  $sd:3.73\cdot{10}^{-7}$ | $9.9\cdot{10}^{-7}$  $sd:9.49\cdot{10}^{-7}$ |
| M1 ROI right | $1.12\cdot{10}^{-5}$  $sd:2.06\cdot{10}^{-6}$ | $9.4\cdot{10}^{-6}$  $sd:2.31\cdot{10}^{-6}$ | $9.77\cdot{10}^{-6}$  $sd:1.74\cdot{10}^{-6}$ | $8.76\cdot{10}^{-6}$  $sd:1.8\cdot{10}^{-6}$ | / | | $2.77\cdot{10}^{-8}$  $sd:2.94\cdot{10}^{-8}$ | $1.44\cdot{10}^{-7}$  $sd:2.16\cdot{10}^{-7}$ | $8.64\cdot{10}^{-7}$  $sd:8.48\cdot{10}^{-7}$ |
| Electr. ROI FPZ | $2.64\cdot{10}^{-5}$  $sd:4.64\cdot{10}^{-6}$ | $2.55\cdot{10}^{-5}$  $sd:6.31\cdot{10}^{-6}$ | $2.44\cdot{10}^{-5}$  $sd:7.27\cdot{10}^{-6}$ | $2.44\cdot{10}^{-5}$  $sd:5.7\cdot{10}^{-6}$ | / | | $1.16\cdot{10}^{-7}$  $sd:1.43\cdot{10}^{-7}$ | $1.42\cdot{10}^{-7}$  $sd:1.25\cdot{10}^{-7}$ | $4.27\cdot{10}^{-7}$  $sd:6.66\cdot{10}^{-7}$ |
| Electr. ROI OZ | $3.58\cdot{10}^{-5}$  $sd:1.84\cdot{10}^{-5}$ | $4.01\cdot{10}^{-5}$  $sd:2.05\cdot{10}^{-5}$ | $4.23\cdot{10}^{-5}$  $sd:2.32\cdot{10}^{-5}$ | $3.06\cdot{10}^{-5}$  $sd:1.18\cdot{10}^{-5}$ | / | | $1.35\cdot{10}^{-7}$  $sd:1.69\cdot{10}^{-7}$ | $3.17\cdot{10}^{-7}$  $sd:3.47\cdot{10}^{-7}$ | $2.98\cdot{10}^{-6}$  $sd:6.09\cdot{10}^{-6}$ |

**Supplementary Table S2.2** Group means and standard deviations of the Sobol indices in the mid-layer ROIs for the ***frontal (FPZ)-occipital (OZ)*** electrode montage. The means and standard deviations of the Sobol indices of the tissue classes skin, skull, cerebrospinal fluid, gray matter, white matter and white matter lesions in the five regions of interest at the cortical mid-layer for each group are listed here.

| V^2^/m^2^ | *Sobol index skin* | | | | *Sobol index skull* | | | | |
| --- | --- | --- | --- | --- | --- | --- | --- | --- | --- |
|  | ***Fazekas 0*** | ***Fazekas 1*** | ***Fazekas 2*** | ***Fazekas 3*** | ***Fazekas 0*** | ***Fazekas 1*** | ***Fazekas 2*** | ***Fazekas 3*** | |
| Hippocamp. Left | $3.53\cdot{10}^{-5}$  $sd:8.11\cdot{10}^{-6}$ | $3.44\cdot{10}^{-5}$  $sd:1.11\cdot{10}^{-5}$ | $3.39\cdot{10}^{-5}$  $sd:7.43\cdot{10}^{-6}$ | $3.04\cdot{10}^{-5}$  $sd:8.52\cdot{10}^{-6}$ | $1.7\cdot{10}^{-5}$  $sd:4.13\cdot{10}^{-6}$ | $1.63\cdot{10}^{-5}$  $sd:6.56\cdot{10}^{-6}$ | $1.64\cdot{10}^{-5}$  $sd:6.81\cdot{10}^{-6}$ | $1.99\cdot{10}^{-5}$  $sd:7.33\cdot{10}^{-6}$ | |
| Hippocamp.  Right | $3.27\cdot{10}^{-5}$  $sd:8.41\cdot{10}^{-6}$ | $3.35\cdot{10}^{-5}$  $sd:1.25\cdot{10}^{-5}$ | $3.56\cdot{10}^{-5}$  $sd:1.02\cdot{10}^{-5}$ | $3.05\cdot{10}^{-5}$  $sd:8.35\cdot{10}^{-6}$ | $1.47\cdot{10}^{-5}$  $sd:3.35\cdot{10}^{-6}$ | $1.46\cdot{10}^{-5}$  $sd:7.25\cdot{10}^{-6}$ | $1.66\cdot{10}^{-5}$  $sd:9.4\cdot{10}^{-6}$ | $1.83\cdot{10}^{-5}$  $sd:6.85\cdot{10}^{-6}$ | |
| Thalamus  Left | $4.33\cdot{10}^{-5}$  $sd:9.64\cdot{10}^{-6}$ | $4.36\cdot{10}^{-5}$  $sd:1.34\cdot{10}^{-5}$ | $4.39\cdot{10}^{-5}$  $sd:1.1\cdot{10}^{-5}$ | $3.87\cdot{10}^{-5}$  $sd:9.1\cdot{10}^{-6}$ | $2.67\cdot{10}^{-5}$  $sd:5.52\cdot{10}^{-6}$ | $2.59\cdot{10}^{-5}$  $sd:8.35\cdot{10}^{-6}$ | $2.58\cdot{10}^{-5}$  $sd:7.29\cdot{10}^{-6}$ | $2.93\cdot{10}^{-5}$  $sd:8.88\cdot{10}^{-6}$ | |
| Thalamus  Right | $4.18\cdot{10}^{-5}$  $sd:9.82\cdot{10}^{-6}$ | $4.32\cdot{10}^{-5}$  $sd:1.39\cdot{10}^{-5}$ | $4.45\cdot{10}^{-5}$  $sd:1.28\cdot{10}^{-5}$ | $3.84\cdot{10}^{-5}$  $sd:1.18\cdot{10}^{-5}$ | $2.55\cdot{10}^{-5}$  $sd:5.09\cdot{10}^{-6}$ | $2.58\cdot{10}^{-5}$  $sd:8.94\cdot{10}^{-6}$ | $2.61\cdot{10}^{-5}$  $sd:9.96\cdot{10}^{-6}$ | $2.81\cdot{10}^{-5}$  $sd:8.5\cdot{10}^{-6}$ | |
|  | **Sobol index gray matter** | | | | **Sobol index white matter** | | | |  |
| Hippocamp. Left | $1.37\cdot{10}^{-5}$  $sd:5.12\cdot{10}^{-6}$ | $2.32\cdot{10}^{-5}$  $sd:3.02\cdot{10}^{-5}$ | $1.6\cdot{10}^{-5}$  $sd:6.09\cdot{10}^{-6}$ | $1.77\cdot{10}^{-5}$  $sd:7.14\cdot{10}^{-6}$ | $9.34\cdot{10}^{-5}$  $sd:3.18\cdot{10}^{-5}$ | $8.0\cdot{10}^{-5}$  $sd:3.32\cdot{10}^{-5}$ | $8.03\cdot{10}^{-5}$  $sd:2.88\cdot{10}^{-5}$ | $6.92\cdot{10}^{-5}$  $sd:3.09\cdot{10}^{-5}$ | |
| Hippocamp.  Right | $1.89\cdot{10}^{-5}$  $sd:1.43\cdot{10}^{-5}$ | $1.78\cdot{10}^{-5}$  $sd:7.78\cdot{10}^{-6}$ | $2.69\cdot{10}^{-5}$  $sd:4.57\cdot{10}^{-5}$ | $2.5\cdot{10}^{-5}$  $sd:1.51\cdot{10}^{-5}$ | $1.09\cdot{10}^{-4}$  $sd:4.06\cdot{10}^{-5}$ | $1.03\cdot{10}^{-4}$  $sd:4.23\cdot{10}^{-5}$ | $1.02\cdot{10}^{-4}$  $sd:3.69\cdot{10}^{-5}$ | $8.28\cdot{10}^{-5}$  $sd:3.35\cdot{10}^{-5}$ | |
| Thalamus  Left | $9.0\cdot{10}^{-6}$  $sd:4.27\cdot{10}^{-6}$ | $1.45\cdot{10}^{-5}$  $sd:1.47\cdot{10}^{-5}$ | $1.17\cdot{10}^{-5}$  $sd:4.56\cdot{10}^{-6}$ | $2.16\cdot{10}^{-5}$  $sd:9.16\cdot{10}^{-6}$ | $7.33\cdot{10}^{-5}$  $sd:3.16\cdot{10}^{-5}$ | $6.72\cdot{10}^{-5}$  $sd:2.94\cdot{10}^{-5}$ | $6.82\cdot{10}^{-5}$  $sd:3.61\cdot{10}^{-5}$ | $5.22\cdot{10}^{-5}$  $sd:1.47\cdot{10}^{-5}$ | |
| Thalamus  Right | $8.2\cdot{10}^{-6}$  $sd:4.11\cdot{10}^{-6}$ | $9.06\cdot{10}^{-6}$  $sd:4.26\cdot{10}^{-6}$ | $1.47\cdot{10}^{-5}$  $sd:1.75\cdot{10}^{-5}$ | $2.26\cdot{10}^{-5}$  $sd:1.36\cdot{10}^{-5}$ | $7.05\cdot{10}^{-5}$  $sd:3.39\cdot{10}^{-5}$ | $6.76\cdot{10}^{-5}$  $sd:2.68\cdot{10}^{-5}$ | $7.43\cdot{10}^{-5}$  $sd:3.88\cdot{10}^{-5}$ | $5.85\cdot{10}^{-5}$  $sd:3.21\cdot{10}^{-5}$ | |
|  | **Sobol index cerebrospinal fluid** | | | | **Sobol index white matter lesions** | | | | |
| Hippocamp. Left | $6.43\cdot{10}^{-6}$  $sd:1.51\cdot{10}^{-6}$ | $6.25\cdot{10}^{-6}$  $sd:1.79\cdot{10}^{-6}$ | $6.05\cdot{10}^{-6}$  $sd:1.29\cdot{10}^{-6}$ | $5.18\cdot{10}^{-6}$  $sd:1.29\cdot{10}^{-6}$ | / | $2.44\cdot{10}^{-6}$  $sd:6.19\cdot{10}^{-6}$ | $1.4\cdot{10}^{-6}$  $sd:2.83\cdot{10}^{-6}$ | $2.02\cdot{10}^{-6}$  $sd:2.79\cdot{10}^{-6}$ | |
| Hippocamp.  Right | $5.95\cdot{10}^{-6}$  $sd:1.23\cdot{10}^{-6}$ | $6.12\cdot{10}^{-6}$  $sd:2.23\cdot{10}^{-6}$ | $5.54\cdot{10}^{-6}$  $sd:1.42\cdot{10}^{-6}$ | $4.71\cdot{10}^{-5}$  $sd:1.65\cdot{10}^{-6}$ | / | $5.7\cdot{10}^{-7}$  $sd:1.34\cdot{10}^{-6}$ | $1.18\cdot{10}^{-6}$  $sd:2.34\cdot{10}^{-6}$ | $1.29\cdot{10}^{-6}$  $sd:2.03\cdot{10}^{-6}$ | |
| Thalamus  Left | $1.04\cdot{10}^{-5}$  $sd:1.75\cdot{10}^{-6}$ | $1.06\cdot{10}^{-5}$  $sd:2.95\cdot{10}^{-6}$ | $1.01\cdot{10}^{-5}$  $sd:2.21\cdot{10}^{-6}$ | $1.0\cdot{10}^{-5}$  $sd:2.23\cdot{10}^{-6}$ | / | $1.06\cdot{10}^{-6}$  $sd:2.07\cdot{10}^{-6}$ | $3.65\cdot{10}^{-6}$  $sd:7.01\cdot{10}^{-6}$ | $4.36\cdot{10}^{-6}$  $sd:8.6\cdot{10}^{-6}$ | |
| Thalamus  Right | $1.06\cdot{10}^{-5}$  $sd:1.83\cdot{10}^{-6}$ | $1.11\cdot{10}^{-5}$  $sd:3.35\cdot{10}^{-6}$ | $9.95\cdot{10}^{-6}$  $sd:2.29\cdot{10}^{-6}$ | $9.92\cdot{10}^{-6}$  $sd:2.12\cdot{10}^{-6}$ | / | $4.51\cdot{10}^{-6}$  $sd:1.12\cdot{10}^{-5}$ | $3.12\cdot{10}^{-6}$  $sd:3.19\cdot{10}^{-6}$ | $1.07\cdot{10}^{-5}$  $sd:3.54\cdot{10}^{-5}$ | |

**Supplementary Table S2.3** Group means and standard deviations of the Sobol indices in the deep ROIs for the ***bihemispheric*** electrode montage. The means and standard deviations of the Sobol indices of the tissue classes skin, skull, cerebrospinal fluid, gray matter, white matter and white matter lesions in the four subcortical regions of interest of each group are listed here.

| V^2^/m^2^ | *Sobol index skin* | | | | *Sobol index skull* | | | | |
| --- | --- | --- | --- | --- | --- | --- | --- | --- | --- |
|  | ***Fazekas 0*** | ***Fazekas 1*** | ***Fazekas 2*** | ***Fazekas 3*** | ***Fazekas 0*** | ***Fazekas 1*** | ***Fazekas 2*** | ***Fazekas 3*** | |
| Hippocamp. left | $9.98\cdot{10}^{-5}$  $sd:1.77\cdot{10}^{-5}$ | $9.98\cdot{10}^{-5}$  $sd:2.37\cdot{10}^{-5}$ | $9.09\cdot{10}^{-5}$  $sd:1.5\cdot{10}^{-5}$ | $9.37\cdot{10}^{-5}$  $sd:2.44\cdot{10}^{-5}$ | $4.99\cdot{10}^{-5}$  $sd:1.01\cdot{10}^{-5}$ | $4.73\cdot{10}^{-5}$  $sd:1.38\cdot{10}^{-5}$ | $4.61\cdot{10}^{-5}$  $sd:1.15\cdot{10}^{-5}$ | $6.05\cdot{10}^{-5}$  $sd:1.86\cdot{10}^{-5}$ | |
| Hippocamp.  Right | $9.55\cdot{10}^{-5}$  $sd:1.85\cdot{10}^{-5}$ | $9.87\cdot{10}^{-5}$  $sd:1.89\cdot{10}^{-5}$ | $9.0\cdot{10}^{-5}$  $sd:1.88\cdot{10}^{-5}$ | $9.37\cdot{10}^{-5}$  $sd:1.89\cdot{10}^{-5}$ | $4.5\cdot{10}^{-5}$  $sd:1.13\cdot{10}^{-5}$ | $4.45\cdot{10}^{-5}$  $sd:1.24\cdot{10}^{-5}$ | $4.16\cdot{10}^{-5}$  $sd:1.28\cdot{10}^{-5}$ | $5.8\cdot{10}^{-5}$  $sd:1.52\cdot{10}^{-5}$ | |
| Thalamus  Left | $1.16\cdot{10}^{-4}$  $sd:1.9\cdot{10}^{-5}$ | $1.15\cdot{10}^{-4}$  $sd:2.46\cdot{10}^{-5}$ | $1.1\cdot{10}^{-4}$  $sd:2.17\cdot{10}^{-5}$ | $1.01\cdot{10}^{-4}$  $sd:2.48\cdot{10}^{-5}$ | $7.09\cdot{10}^{-5}$  $sd:1.26\cdot{10}^{-5}$ | $6.68\cdot{10}^{-5}$  $sd:1.47\cdot{10}^{-5}$ | $6.68\cdot{10}^{-5}$  $sd:1.58\cdot{10}^{-5}$ | $7.35\cdot{10}^{-5}$  $sd:2.0\cdot{10}^{-5}$ | |
| Thalamus  Right | $1.11\cdot{10}^{-4}$  $sd:1.94\cdot{10}^{-5}$ | $1.12\cdot{10}^{-4}$  $sd:2.54\cdot{10}^{-5}$ | $1.07\cdot{10}^{-4}$  $sd:2.24\cdot{10}^{-5}$ | $1.0\cdot{10}^{-4}$  $sd:2.66\cdot{10}^{-5}$ | $6.71\cdot{10}^{-5}$  $sd:1.32\cdot{10}^{-5}$ | $6.41\cdot{10}^{-5}$  $sd:1.41\cdot{10}^{-5}$ | $6.31\cdot{10}^{-5}$  $sd:1.6\cdot{10}^{-5}$ | $7.21\cdot{10}^{-5}$  $sd:2.08\cdot{10}^{-5}$ | |
|  | **Sobol index gray matter** | | | | **Sobol index white matter** | | | |  |
| Hippocamp. left | $1.88\cdot{10}^{-5}$  $sd:6.85\cdot{10}^{-6}$ | $2.5\cdot{10}^{-5}$  $sd:1.1\cdot{10}^{-5}$ | $1.89\cdot{10}^{-5}$  $sd:7.35\cdot{10}^{-6}$ | $2.34\cdot{10}^{-5}$  $sd:1.07\cdot{10}^{-5}$ | $1.49\cdot{10}^{-4}$  $sd:3.73\cdot{10}^{-5}$ | $1.64\cdot{10}^{-4}$  $sd:7.01\cdot{10}^{-5}$ | $1.33\cdot{10}^{-4}$  $sd:5.33\cdot{10}^{-5}$ | $1.26\cdot{10}^{-4}$  $sd:4.63\cdot{10}^{-5}$ | |
| Hippocamp.  Right | $2.44\cdot{10}^{-5}$  $sd:1.15\cdot{10}^{-5}$ | $2.79\cdot{10}^{-5}$  $sd:1.24\cdot{10}^{-5}$ | $2.23\cdot{10}^{-5}$  $sd:1.33\cdot{10}^{-5}$ | $3.24\cdot{10}^{-5}$  $sd:1.91\cdot{10}^{-5}$ | $1.48\cdot{10}^{-4}$  $sd:4.63\cdot{10}^{-5}$ | $1.65\cdot{10}^{-4}$  $sd:5.56\cdot{10}^{-5}$ | $1.41\cdot{10}^{-4}$  $sd:5.56\cdot{10}^{-5}$ | $1.38\cdot{10}^{-4}$  $sd:4.46\cdot{10}^{-5}$ | |
| Thalamus  Left | $1.73\cdot{10}^{-5}$  $sd:6.72\cdot{10}^{-6}$ | $2.14\cdot{10}^{-5}$  $sd:7.39\cdot{10}^{-6}$ | $2.14\cdot{10}^{-5}$  $sd:8.09\cdot{10}^{-6}$ | $3.55\cdot{10}^{-5}$  $sd:1.22\cdot{10}^{-5}$ | $1.44\cdot{10}^{-4}$  $sd:3.76\cdot{10}^{-5}$ | $1.93\cdot{10}^{-4}$  $sd:1.5\cdot{10}^{-4}$ | $1.39\cdot{10}^{-4}$  $sd:5.65\cdot{10}^{-5}$ | $1.0\cdot{10}^{-4}$  $sd:3.83\cdot{10}^{-5}$ | |
| Thalamus  Right | $1.46\cdot{10}^{-5}$  $sd:6.36\cdot{10}^{-6}$ | $2.03\cdot{10}^{-5}$  $sd:1.09\cdot{10}^{-5}$ | $2.12\cdot{10}^{-5}$  $sd:7.77\cdot{10}^{-6}$ | $3.77\cdot{10}^{-5}$  $sd:1.6\cdot{10}^{-5}$ | $1.33\cdot{10}^{-4}$  $sd:3.48\cdot{10}^{-5}$ | $1.81\cdot{10}^{-4}$  $sd:1.44\cdot{10}^{-4}$ | $1.35\cdot{10}^{-4}$  $sd:5.84\cdot{10}^{-5}$ | $1.09\cdot{10}^{-4}$  $sd:4.97\cdot{10}^{-5}$ | |
|  | **Sobol index cerebrospinal fluid** | | | | **Sobol index white matter lesions** | | | | |
| Hippocamp. left | $1.61\cdot{10}^{-5}$  $sd:2.27\cdot{10}^{-6}$ | $1.51\cdot{10}^{-5}$  $sd:2.24\cdot{10}^{-6}$ | $1.57\cdot{10}^{-5}$  $sd:2.4\cdot{10}^{-6}$ | $1.52\cdot{10}^{-5}$  $sd:2.37\cdot{10}^{-6}$ | / | $7.89\cdot{10}^{-7}$  $sd:1.72\cdot{10}^{-6}$ | $1.01\cdot{10}^{-6}$  $sd:1.64\cdot{10}^{-6}$ | $2.25\cdot{10}^{-6}$  $sd:2.55\cdot{10}^{-6}$ | |
| Hippocamp.  Right | $1.66\cdot{10}^{-5}$  $sd:2.76\cdot{10}^{-6}$ | $1.57\cdot{10}^{-5}$  $sd:2.25\cdot{10}^{-6}$ | $1.63\cdot{10}^{-5}$  $sd:2.91\cdot{10}^{-6}$ | $1.5\cdot{10}^{-5}$  $sd:2.29\cdot{10}^{-6}$ | / | $5.61\cdot{10}^{-7}$  $sd:9.75\cdot{10}^{-7}$ | $8.03\cdot{10}^{-7}$  $sd:1.15\cdot{10}^{-6}$ | $2.63\cdot{10}^{-6}$  $sd:2.81\cdot{10}^{-6}$ | |
| Thalamus  Left | $1.83\cdot{10}^{-5}$  $sd:3.02\cdot{10}^{-6}$ | $1.63\cdot{10}^{-5}$  $sd:3.78\cdot{10}^{-6}$ | $1.77\cdot{10}^{-5}$  $sd:3.31\cdot{10}^{-6}$ | $1.55\cdot{10}^{-5}$  $sd:2.12\cdot{10}^{-6}$ | / | $2.54\cdot{10}^{-6}$  $sd:6.4\cdot{10}^{-6}$ | $1.53\cdot{10}^{-6}$  $sd:2.56\cdot{10}^{-6}$ | $2.63\cdot{10}^{-6}$  $sd:2.63\cdot{10}^{-6}$ | |
| Thalamus  Right | $1.89\cdot{10}^{-5}$  $sd:2.77\cdot{10}^{-6}$ | $1.7\cdot{10}^{-5}$  $sd:3.65\cdot{10}^{-6}$ | $1.8\cdot{10}^{-5}$  $sd:3.37\cdot{10}^{-6}$ | $1.53\cdot{10}^{-5}$  $sd:1.82\cdot{10}^{-6}$ | / | $3.55\cdot{10}^{-6}$  $sd:7.67\cdot{10}^{-6}$ | $4.39\cdot{10}^{-6}$  $sd:7.22\cdot{10}^{-6}$ | $5.24\cdot{10}^{-6}$  $sd:6.89\cdot{10}^{-6}$ | |

**Supplementary Table S2.4** Group means and standard deviations of the Sobol indices in the deep ROIs for the ***frontal (FPZ)-occipital (OZ)*** electrode montage. The means and standard deviations of the Sobol indices of the tissue classes skin, skull, cerebrospinal fluid, gray matter, white matter and white matter lesions in the four subcortical regions of interest of each group are listed here.

| Bihemispheric electrode setup | | | | | | | | |
| --- | --- | --- | --- | --- | --- | --- | --- | --- |
|  | mean( $\left\Vert\boldsymbol{E} \right\Vert$) | $Var(\vert\vert\boldsymbol{E\vert}\vert)$ | ***Sobol index skin*** | ***Sobol index skull*** | ***Sobol index cerebrospinal fluid*** | ***Sobol index gray matter*** | ***Sobol index white matter*** | ***Sobol index lesioned white matter*** |
| Whole-brain | $p=.098 \left( P \right)$ | $p=.537 (NP)$ | $p=.143 (P)$ | $p=.575 (P)$ | $p=.884 (P)$ | $p=.436 (NP)$ | $p=.0002 \left( NP \right)$*  $\eta^{2}=.195$ | $p\ll.001\left( NP \right)$*  $\eta^{2}=.669$ |
| M1 ROI left | $p=.237 \left( P \right)$ | $p= .065 (NP)$ | $p=.036 \left( P \right)$ | $p=.05 \left( P \right)$ | $p=.593 (P)$ | $p=.009 (NP)$ | $p\ll.001\left( NP \right)$*  $\eta^{2}=.291$ | $p\ll.001(NP)$*  $\eta^{2}=.553$ |
| M1 ROI right | $p=.706 \left( P \right)$ | $p=.448 (NP)$ | $p=.515 (P)$ | $p=.704 (P)$ | $p=.206 (NP)$ | $p=.259 (NP)$ | $p= .0003 \left( NP \right)$ $\eta^{2}=.189$ | $p\ll.001(NP)$*  $\eta^{2}=.486$ |
| Electr. ROI left | $p= .209 (NP)$ | $p=.348 (NP)$ | $p=.0029 (NP)$ | $p=.075 (NP)$ | $p=.736 (P)$ | $p= .1 (NP)$ | $p=.0035 (NP)$ | $p\ll.001(NP)$*  $\eta^{2}=.617$ |
| Electr. ROI right | $p=.357 (NP)$ | $p=.886 (NP)$ | $p=.078 (NP)$ | $p=.068 (P)$ | $p=.438 (P)$ | $p=.617 (NP)$ | $p=.0184 (NP)$ | $p\ll.001(NP)$*  $\eta^{2}=.614$ |

| Frontal-occipital electrode setup | | | | | | | | |
| --- | --- | --- | --- | --- | --- | --- | --- | --- |
|  | mean( $\left\Vert\boldsymbol{E} \right\Vert$) | $Var(\vert\vert\boldsymbol{E\vert}\vert)$ | ***Sobol index skin*** | ***Sobol index skull*** | ***Sobol index cerebrospinal fluid*** | ***Sobol index gray matter*** | ***Sobol index white matter*** | ***Sobol index lesioned white matter*** |
| Whole-brain | $p=.001 \left( P \right)$*  $\eta^{2}=.152$ | $p=.0024 \left( P \right)$ | $p=.001 \left( P \right)*$  $\eta^{2}=.14$ | $p=.017 \left( P \right)$ | $p=.094 \left( P \right)$ | $p=.113 \left( NP \right)$ | $p\ll.001\left( NP \right)$*  $\eta^{2}=.482$ | $p\ll.001\left( NP \right)$*  $\eta^{2}=.617$ |
| M1 ROI left | $p\ll.001\left( P \right)$* $\eta^{2}=.198$ | $p\ll.001\left( NP \right)$*$\eta^{2}=.27$ | $p\ll.001\left( NP \right)$*$\eta^{2}=.244$ | $p=.003 \left( NP \right)$ | $p=.021 \left( P \right)$ | $p=.008 \left( NP \right)$ | $p\ll.001\left( NP \right)$*  $\eta^{2}=.462$ | $p\ll.001(NP)$*  $\eta^{2}=.517$ |
| M1 ROI right | $p\ll.001\left( P \right)$* $\eta^{2}=.193$ | $p\ll.001\left( NP \right)$* $\eta^{2}=.224$ | $p=.0015 \left( NP \right)$* $\eta^{2}=.206$ | $p=.008 \left( NP \right)$ | $p=.001 \left( P \right)$*$\eta^{2}=.13$ | $p=.001 \left( NP \right)$* $\eta^{2}=.175$ | $p\ll.001\left( NP \right)$*  $\eta^{2}=.439$ | $p\ll.001\left( NP \right)$*  $\eta^{2}=.566$ |
| Electr. ROI frontal | $p= .371 (P)$ | $p=.491 (NP)$ | $p=.241 (NP)$ | $p=.344 (NP)$ | $p=.65 (P)$ | $p= .365 (NP)$ | $p=.0014 \left( NP \right)$*  $\eta^{2}=.149$ | $p=.012(NP)$ |
| Electr. ROI occipital | $p=.007 (NP)$ | $p=.006 (NP)$ | $p=.0024 (NP)$ | $p=.006 (P)$ | $p=.251 (NP)$ | $p=.03 (NP)$ | $p\ll.001 (NP)$*  $\eta^{2}=.428$ | $p\ll.001(NP)$*  $\eta^{2}=.396$ |

**Supplementary Table S3.1** Results of the statistical hypothesis tests of selected outcomes of the sensitivity analysis in the mid-layer ROIs between all groups with both electrode montages. *This table contains the p-values of the significance tests of the variables: mean electric field magnitude, its variance and the Sobol indices of skin, skull, cerebrospinal fluid, gray matter, white matter and lesioned white matter. Significant (significance threshold* $\alpha\leq0.002$*, corrected for the number of comparisons in 5 mid-layer ROIs from a base threshold of 0.01) deviations of group means are* *asterisked. Effect sizes of significant differences are reported as* $\eta^{2}$*measures based on the H-statistic of the Kruskal-Wallis test* [63]*. Abbreviations: NP = non-parametric test, Kruskal-Wallis; P = parametric test, one-way ANOVA.*

| Bihemispheric electode montage | | | | | | | | |
| --- | --- | --- | --- | --- | --- | --- | --- | --- |
|  | mean( $\left\Vert\boldsymbol{E} \right\Vert$) | $Var(\vert\vert\boldsymbol{E\vert}\vert)$ | ***Sobol index skin*** | ***Sobol index skull*** | ***Sobol index cerebrospinal fluid*** | ***Sobol index gray matter*** | ***Sobol index white matter*** | ***Sobol index lesioned white matter*** |
| Hippocamp. left | $p=.022\left( NP \right)$ | $p=.255\left( NP \right)$ | $p=.284\left( P \right)$ | $p=.195 \left( P \right)$ | $p=.519 \left( P \right)$ | $p=.008 \left( NP \right)$ | $p=.022\left( NP \right)$ | $p=.001\left( NP \right)$*  $\eta^{2}=.180$ |
| Hippocamp.  right | $p=.143\left( P \right)$ | $p=.456\left( NP \right)$ | $p=.412 \left( P \right)$ | $p=.174\left( NP \right)$ | $p=.231 \left( NP \right)$ | $p=.119 \left( NP \right)$ | $p=.056\left( NP \right)$ | $p\ll.001\left( NP \right)$*  $\eta^{2}=.263$ |
| Thalamus  left | $p= .09 (P)$ | $p=.98 (NP)$ | $p=.336 (P)$ | $p=.51 (NP)$ | $p=.847 (P)$ | $p\ll.001(NP)$*  $\eta^{2}=.299$ | $p=.084 \left( NP \right)$ | $p=.0017(NP)$*  $\eta^{2}=.170$ |
| Thalamus  right | $p=.1 (P)$ | $p=.884 (NP)$ | $p=.362 (NP)$ | $p=.67 (NP)$ | $p=.527 (NP)$ | $p\ll.001(NP)$*  $\eta^{2}=.293$ | $p=.358 (NP)$ | $p=.116(NP)$ |

| Frontal-occipital electrode setup | | | | | | | | |
| --- | --- | --- | --- | --- | --- | --- | --- | --- |
|  | $mean( \left\Vert\boldsymbol{E} \right\Vert)$ | $Var(\vert\vert\boldsymbol{E\vert}\vert)$ | ***Sobol index skin*** | ***Sobol index skull*** | ***Sobol index cerebrospinal fluid*** | ***Sobol index gray matter*** | ***Sobol index white matter*** | ***Sobol index lesioned white matter*** |
| Hippocamp. left | $p=.062\left( P \right)$ | $p=.249\left( NP \right)$ | $p=.236\left( NP \right)$ | $p=.035 \left( NP \right)$ | $p=.478 \left( P \right)$ | $p=.144 \left( NP \right)$ | $p=.063\left( NP \right)$ | $p=.001\left( NP \right)$*  $\eta^{2}=.186$ |
| Hippocamp.  right | $p=.088\left( P \right)$ | $p=.3\left( NP \right)$ | $p=.64 \left( NP \right)$ | $p=.002\left( NP \right)$*  $\eta^{2}=.147$ | $p=.199 \left( P \right)$ | $p=.089 \left( NP \right)$ | $p=.3\left( P \right)$ | $p\ll.001\left( NP \right)$*  $\eta^{2}=.264$ |
| Thalamus  left | $p= .011 (P)$ | $p=.172 (NP)$ | $p=.12 (P)$ | $p=.437 (P)$ | $p=.017 (P)$ | $p\ll.001(NP)$*  $\eta^{2}=.327$ | $p=.0035 \left( NP \right)$ | $p=.0013(NP)$*  $\eta^{2}=.179$ |
| Thalamus  right | $p=.023(P)$ | $p=.714 (NP)$ | $p=.353 (P)$ | $p=.262 (P)$ | $p=.001 \left( P \right)$*  $\eta^{2}=.174$ | $p\ll.001(NP)$*  $\eta^{2}=.374$ | $p=.078 (NP)$ | $p=.029(NP)$ |

**Supplementary Table S3.2** Results of the statistical hypothesis tests of selected outcomes of the sensitivity analysis in the deep ROIs between all groups with both electrode montages. *This table contains the p-values of the significance tests of the variables: mean electric field magnitude, its variance and the Sobol indices of skin, skull, cerebrospinal fluid, gray matter, white matter and lesioned white matter. Significant (significance threshold* $\alpha\leq0.0025$*, corrected for the number of comparisons in 4 deep ROIs from a base threshold of 0.01) deviations of group means are* *asterisked. Effect sizes of significant differences are reported as* $\eta^{2}$*measures based on the H-statistic of the Kruskal-Wallis test* [63]*. Abbreviations: NP = non-parametric test, Kruskal-Wallis; P = parametric test, one-way ANOVA.*

|  | Sobol index white matter | Sobol index lesion |
| --- | --- | --- |
| Whole-brain | \|  \| F0 \| F1 \| F2 \| \| --- \| --- \| --- \| --- \| \| F1 \| $1.0$ \|  \|  \| \| F2 \| $1.0$ \| $1.0$ \| $\times$ \| \| F3 \| $.0002$ \| $.0049$ \| $.0016$ \| | \|  \| F1 \| F2 \| \| --- \| --- \| --- \| \| F2 \| $.183$ \| $\times$ \| \| F3 \| $\ll.001$ \| $\ll.001$ \| |
| Left M1 ROI | \|  \| F0 \| F1 \| F2 \| \| --- \| --- \| --- \| --- \| \| F1 \| $0.071$ \|  \|  \| \| F2 \| $0.707$ \| $0.849$ \| $\times$ \| \| F3 \| $\ll.001$ \| $.0182$ \| $.0004$ \| | \|  \| F1 \| F2 \| \| --- \| --- \| --- \| \| F2 \| $.273$ \| $\times$ \| \| F3 \| $\ll.001$ \| $\ll.001$ \| |
| Right M1 ROI | \|  \| F0 \| F1 \| F2 \| \| --- \| --- \| --- \| --- \| \| F1 \| 0.577 \|  \|  \| \| F2 \| $1.0$ \| $1.0$ \| $\times$ \| \| F3 \| $.0001$ \| $.0158$ \| $.0033$ \| | \|  \| F1 \| F2 \| \| --- \| --- \| --- \| \| F2 \| $.501$ \| $\times$ \| \| F3 \| $\ll.001$ \| $\ll.001$ \| |
| Electrode ROI left |  | \|  \| F1 \| F2 \| \| --- \| --- \| --- \| \| F2 \| $.1219$ \| $\times$ \| \| F3 \| $\ll.001$ \| $\ll.001$ \| |
| Electrode ROI right |  | \|  \| F1 \| F2 \| \| --- \| --- \| --- \| \| F2 \| $.2097$ \| $\times$ \| \| F3 \| $\ll.001$ \| $\ll.001$ \| |

**Supplementary Table S4.1** Results of the post-hoc statistical hypothesis tests in the mid-layer ROIs with the **bihemispheric** electrode montage. *This table contains the p-values of the pos-hoc test following statistical hypothesis testing between all groups. Significant (significance threshold* $\alpha\leq0.01$*, familywise error corrected) deviations of group means are highlighted in red. Abbreviations: F0/1/2/3 = Fazekas 0 (absence of lesions)/ 1 (low lesion load)/2 (medium lesion load)/3 (high lesion load) group. Non-parametric post-hoc test: Dunn’s test.*

|  | $\mathbf{mean(}\left\Vert\boldsymbol{E} \right\Vert\mathbf{)}$ | $\boldsymbol{Var(\vert\vert E\vert\vert)}$ | Sobol index scalp | Sobol index cerebrospinal fluid |
| --- | --- | --- | --- | --- |
| Whole-brain | \|  \| F0 \| F1 \| F2 \| \| --- \| --- \| --- \| --- \| \| F1 \| $1.0$ \|  \|  \| \| F2 \| $1.0$ \| $1.0$ \| $\times$ \| \| F3 \| $.0015$ \| $.0218$ \| $.0027$ \| |  | \|  \| F0 \| F1 \| F2 \| \| --- \| --- \| --- \| --- \| \| F1 \| $1.0$ \|  \|  \| \| F2 \| $1.0$ \| $1.0$ \| $\times$ \| \| F3 \| $.0013$ \| $.0038$ \| $.0028$ \| |  |
| Left M1 ROI | \|  \| F0 \| F1 \| F2 \| \| --- \| --- \| --- \| --- \| \| F1 \| $.081$ \|  \|  \| \| F2 \| $1.0$ \| $.219$ \| $\times$ \| \| F3 \| $.0002$ \| $.343$ \| $.0007$ \| | \|  \| F0 \| F1 \| F2 \| \| --- \| --- \| --- \| --- \| \| F1 \| $.135$ \|  \|  \| \| F2 \| $1.0$ \| $.127$ \| $\times$ \| \| F3 \| $.0002$ \| $.1422$ \| $.0002$ \| | \|  \| F0 \| F1 \| F2 \| \| --- \| --- \| --- \| --- \| \| F1 \| $.049$ \|  \|  \| \| F2 \| $.849$ \| $.779$ \| $\times$ \| \| F3 \| $\ll.001$ \| $.0845$ \| $.0013$ \| |  |
| Right M1 ROI | \|  \| F0 \| F1 \| F2 \| \| --- \| --- \| --- \| --- \| \| F1 \| $.261$ \|  \|  \| \| F2 \| $1.0$ \| $0.445$ \| $\times$ \| \| F3 \| $.0003$ \| $.182$ \| $.0008$ \| | \|  \| F0 \| F1 \| F2 \| \| --- \| --- \| --- \| --- \| \| F1 \| $1.0$ \|  \|  \| \| F2 \| $.939$ \| $.202$ \| $\times$ \| \| F3 \| $.0017$ \| $.026$ \| $.\ll.001$ \| | \|  \| F0 \| F1 \| F2 \| \| --- \| --- \| --- \| --- \| \| F1 \| $.271$ \|  \|  \| \| F2 \| $1.0$ \| $1.0$ \| $\times$ \| \| F3 \| $.0001$ \| $.00297$ \| $.0023$ \| | \|  \| F0 \| F1 \| F2 \| \| --- \| --- \| --- \| --- \| \| F1 \| $.027$ \|  \|  \| \| F2 \| $.14$ \| $1.0$ \| $\times$ \| \| F3 \| $.0008$ \| $1.0$ \| $.578$ \| |
| Electrode ROI FPZ |  |  | $\times$ |  |
| Electrode ROI OZ |  |  | $\times$ |  |

|  | Sobol index gray matter | Sobol index white matter | Sobol index lesion |
| --- | --- | --- | --- |
| Whole-brain |  | \|  \| F0 \| F1 \| F2 \| \| --- \| --- \| --- \| --- \| \| F1 \| $.097$ \|  \|  \| \| F2 \| $.23$ \| $1.0$ \| $\times$ \| \| F3 \| $\ll.001$ \| $.0001$ \| $\ll.001$ \| | \|  \| F1 \| F2 \| \| --- \| --- \| --- \| \| F2 \| $.122$ \| $\times$ \| \| F3 \| $\ll.001$ \| $\ll.001$ \| |
| Left M1 ROI |  | \|  \| F0 \| F1 \| F2 \| \| --- \| --- \| --- \| --- \| \| F1 \| $.016$ \|  \|  \| \| F2 \| $1.0$ \| $0.125$ \| $\times$ \| \| F3 \| $\ll.001$ \| $.0056$ \| $.0003$ \| | \|  \| F1 \| F2 \| \| --- \| --- \| --- \| \| F2 \| $.027$ \| $\times$ \| \| F3 \| $\ll.001$ \| $.0003$ \| |
| Right M1 ROI | \|  \| F0 \| F1 \| F2 \| \| --- \| --- \| --- \| --- \| \| F1 \| $.965$ \|  \|  \| \| F2 \| $.031$ \| $.35$ \| $\times$ \| \| F3 \| $.384$ \| $.036$ \| $\ll.001$ \| | \|  \| F0 \| F1 \| F2 \| \| --- \| --- \| --- \| --- \| \| F1 \| $.043$ \|  \|  \| \| F2 \| $1.0$ \| $.295$ \| $\times$ \| \| F3 \| $\ll.001$ \| $.0023$ \| $\ll.001$ \| | \|  \| F1 \| F2 \| \| --- \| --- \| --- \| \| F2 \| $.0283$ \| $\times$ \| \| F3 \| $\ll.001$ \| $.0003$ \| |
| Electrode ROI FPZ |  | \|  \| F0 \| F1 \| F2 \| \| --- \| --- \| --- \| --- \| \| F1 \| $.707$ \|  \|  \| \| F2 \| $1.0$ \| $1.0$ \| $\times$ \| \| F3 \| $.0011$ \| $.0514$ \| $.0037$ \| |  |
| Electrode ROI OZ |  | \|  \| F0 \| F1 \| F2 \| \| --- \| --- \| --- \| --- \| \| F1 \| $.757$ \|  \|  \| \| F2 \| $1.0$ \| $1.0$ \| $\times$ \| \| F3 \| $\ll.001$ \| $\ll.001$ \| $\ll.001$ \| | \|  \| F1 \| F2 \| \| --- \| --- \| --- \| \| F2 \| $.191$ \| $\times$ \| \| F3 \| $\ll.001$ \| $.0011$ \| |

**Supplementary Table S4.2** Results of the post-hoc statistical hypothesis tests in the mid-layer ROIs with the ***frontal (FPZ)-occipital (OZ)*** electrode montage. *This table contains the p-values of the pos-hoc test following statistical hypothesis testing between all groups. Significant (significance threshold* $\alpha\leq0.01$*, familywise error corrected) deviations of group means are highlighted in red. Abbreviations: F0/1/2/3 = Fazekas 0 (absence of lesions)/ 1 (low lesion load)/2 (medium lesion load)/3 (high lesion load) group. Non-parametric post-hoc test: Dunn’s test.*

|  | Sobol index gray matter | Sobol index lesion |
| --- | --- | --- |
| Hippocampus  left |  | \|  \| F1 \| F2 \| \| --- \| --- \| --- \| \| F2 \| $.261$ \| $\times$ \| \| F3 \| $.0005$ \| $.036$ \| |
| Hippocampus  right |  | \|  \| F1 \| F2 \| \| --- \| --- \| --- \| \| F2 \| $.083$ \| $\times$ \| \| F3 \| $\ll.001$ \| $.025$ \| |
| Thalamus  left | \|  \| F0 \| F1 \| F2 \| \| --- \| --- \| --- \| --- \| \| F1 \| $.194$ \|  \|  \| \| F2 \| $.21$ \| $1.0$ \| $\times$ \| \| F3 \| $\ll.001$ \| $.0025$ \| $.0022$ \| | \|  \| F1 \| F2 \| \| --- \| --- \| --- \| \| F2 \| $.049$ \| $\times$ \| \| F3 \| $.0006$ \| $.24$ \| |
| Thalamus  right | \|  \| F0 \| F1 \| F2 \| \| --- \| --- \| --- \| --- \| \| F1 \| $1.0$ \|  \|  \| \| F2 \| $.172$ \| $.388$ \| $\times$ \| \| F3 \| $\ll.001$ \| $\ll.001$ \| $.0141$ \| |  |

**Supplementary Table S4.3** Results of the post-hoc statistical hypothesis tests in the deep ROIs with the **bihemispheric** electrode montage. *This table contains the p-values of the pos-hoc test following statistical hypothesis testing between all groups. Significant (significance threshold* $\alpha\leq0.01$*, familywise error corrected) deviations of group means are highlighted in red. Abbreviations: F0/1/2/3 = Fazekas 0 (absence of lesions)/ 1 (low lesion load)/2 (medium lesion load)/3 (high lesion load) group. Non-parametric post-hoc test: Dunn’s test.*

|  | Sobol index skull | Sobol index cerebrospinal fluid | Sobol index gray matter | Sobol index lesion |
| --- | --- | --- | --- | --- |
| Hippocampus  left |  |  |  | \|  \| F1 \| F2 \| \| --- \| --- \| --- \| \| F2 \| $.683$ \| $\times$ \| \| F3 \| $.0007$ \| $.0084$ \| |
| Hippocampus  right | \|  \| F0 \| F1 \| F2 \| \| --- \| --- \| --- \| --- \| \| F1 \| $1.0$ \|  \|  \| \| F2 \| $.881$ \| $1.0$ \| $\times$ \| \| F3 \| $.025$ \| $.012$ \| $.0007$ \| |  |  | \|  \| F1 \| F2 \| \| --- \| --- \| --- \| \| F2 \| $.588$ \| $\times$ \| \| F3 \| $.0001$ \| $.0018$ \| |
| Thalamus  left |  |  | \|  \| F0 \| F1 \| F2 \| \| --- \| --- \| --- \| --- \| \| F1 \| $.216$ \|  \|  \| \| F2 \| $.285$ \| $1.0$ \| $\times$ \| \| F3 \| $\ll.001$ \| $.001$ \| $.0007$ \| | \|  \| F1 \| F2 \| \| --- \| --- \| --- \| \| F2 \| $.614$ \| $\times$ \| \| F3 \| $.0007$ \| $.012$ \| |
| Thalamus  right |  | \|  \| F0 \| F1 \| F2 \| \| --- \| --- \| --- \| --- \| \| F1 \| $.224$ \|  \|  \| \| F2 \| $1.0$ \| $1.0$ \| $\times$ \| \| F3 \| $.0008$ \| $.382$ \| $.019$ \| | \|  \| F0 \| F1 \| F2 \| \| --- \| --- \| --- \| --- \| \| F1 \| $.213$ \|  \|  \| \| F2 \| $.02$ \| $1.0$ \| $\times$ \| \| F3 \| $\ll.001$ \| $.0003$ \| $.0076$ \| |  |

**Supplementary Table S4.3** Results of the post-hoc statistical hypothesis tests in the subcortical ROIs with the **frontal-occipital** electrode montage. *This table contains the p-values of the pos-hoc test following statistical hypothesis testing between all groups. Significant (significance threshold* $\alpha\leq0.01$*, familywise error corrected) deviations of group means are highlighted in red. Abbreviations: F0/1/2/3 = Fazekas 0 (absence of lesions)/ 1 (low lesion load)/2 (medium lesion load)/3 (high lesion load) group. Non-parametric post-hoc test: Dunn’s test.*

1. S.M. Smith, Y. Zhang, M. Jenkinson, J. Chen, P.M. Matthews, A. Federico, and N. De Stefano. “Accurate, robust and automated longitudinal and cross-sectional brain change analysis”, NeuroImage, 17(1):479-489, 2002. [↑](#footnote-ref-1)
2. S.M. Smith, M. Jenkinson, M.W. Woolrich, C.F. Beckmann, T.E.J. Behrens, H. Johansen-Berg, P.R. Bannister, M. De Luca, I. Drobnjak, D.E. Flitney, R. Niazy, J. Saunders, J. Vickers, Y. Zhang, N. De Stefano, J.M. Brady, and P.M. Matthews. “Advances in functional and structural MR image analysis and implementation as FSL”, NeuroImage, 23(S1):208-219, 2004. [↑](#footnote-ref-2)
3. „Comparing and Validating Automated Tools for Individualized Electric Field Simulations in the Human Head“, Puonti et al. 2019, bioRxiv, https://doi.org/10.1101/611962 [↑](#footnote-ref-3)
4. L. Lampe, S. Kharabian-Masouleh, J. Kynast, K. Arelin, C. J. Steele, M. Löffler, A. V. Witte, M. L. Schroeter, A. Villringer und P.-L. Bazin, „Lesion location matters: the relationships between white matter hyperintensities on cognition in the healthy elderly“, *Journal of Cerebral Blood Flow & Metabolism*, vol. 39, p. 36–43, 2019. [↑](#footnote-ref-4)
5. M. A. Mayka, D. M. Corcos, S. E. Leurgans und D. E. Vaillancourt, „Three-dimensional locations and boundaries of motor and premotor cortices as defined by functional brain imaging: a meta-analysis“, *NeuroImage*, vol. 31, p. 1453–1474, 2006 [↑](#footnote-ref-5)
6. L. Fan, H. Li, J. Zhuo, Y. Zhang, J. Wang, L. Chen, Z. Yang, C. Chu, S. Xie, A. R. Laird und others, „The human brainnetome atlas: a new brain atlas based on connectional architecture“, *Cerebral cortex*, vol. 26, p. 3508–3526, 2016. [↑](#footnote-ref-6)
7. B. B. Avants, C. L. Epstein, M. Grossman und J. C. Gee, „Symmetric diffeomorphic image registration with cross-correlation: evaluating automated labeling of elderly and neurodegenerative brain“, *Medical image analysis*, Bd. 12, p. 26–41, 2008. [↑](#footnote-ref-7)
